# A global dataset on phosphorus in agricultural soils

**DOI:** 10.1038/s41597-023-02751-6

**Published:** 2024-01-02

**Authors:** Bruno Ringeval, Josephine Demay, Daniel S. Goll, Xianjin He, Ying-Ping Wang, Enqing Hou, Sarah Matej, Karl-Heinz Erb, Rong Wang, Laurent Augusto, Fei Lun, Thomas Nesme, Pasquale Borrelli, Julian Helfenstein, Richard W. McDowell, Peter Pletnyakov, Sylvain Pellerin

**Affiliations:** 1 ISPA, Bordeaux Sciences Agro, INRAE, 33140 Villenave d’Ornon, France; 2 Université Paris Saclay, CEA-CNRS-UVSQ, LSCE/IPSL, Gif-sur-Yvette, France; 3 CSIRO Oceans and Atmosphere, Aspendale, Vic. Australia; 4 Key Laboratory of Vegetation Restoration and Management of Degraded Ecosystems, South China Botanical Garden, Chinese Academy of Sciences, Guangzhou, China; 5 Institute of Social Ecology, University of Natural Resources and Life Sciences, Vienna, Austria; 6 Department of Environmental Science and Engineering, Fudan University, Shanghai, 200438 China; 7 College of Land Science and Technology, China Agricultural University, Beijing, 100193 China; 8 Department of Science, Roma Tre University, 00146 Rome, Italy; 9 Department of Biological Environment, Kangwon National University, Chuncheon, 24341 Republic of Korea; 10 Soil Geography and Landscape Group, University of Wageningen, Wageningen, 6700AA The Netherlands; 11 AgResearch, Lincoln Science Centre, Private Bag 4749, Christchurch, 8140 New Zealand; 12 Faculty of Agriculture and Life Sciences, Lincoln University, Lincoln, PO Box 84, 7647 Christchurch, New Zealand

**Keywords:** Element cycles, Environmental sciences

## Abstract

Numerous drivers such as farming practices, erosion, land-use change, and soil biogeochemical background, determine the global spatial distribution of phosphorus (P) in agricultural soils. Here, we revised an approach published earlier (called here GPASOIL-v0), in which several global datasets describing these drivers were combined with a process model for soil P dynamics to reconstruct the past and current distribution of P in cropland and grassland soils. The objective of the present update, called GPASOIL-v1, is to incorporate recent advances in process understanding about soil inorganic P dynamics, in datasets to describe the different drivers, and in regional soil P measurements for benchmarking. We trace the impact of the update on the reconstructed soil P. After the update we estimate a global averaged inorganic labile P of 187 kgP ha^−1^ for cropland and 91 kgP ha^−1^ for grassland in 2018 for the top 0–0.3 m soil layer, but these values are sensitive to the mineralization rates chosen for the organic P pools. Uncertainty in the driver estimates lead to coefficients of variation of 0.22 and 0.54 for cropland and grassland, respectively. This work makes the methods for simulating the agricultural soil P maps more transparent and reproducible than previous estimates, and increases the confidence in the new estimates, while the evaluation against regional dataset still suggests rooms for further improvement.

## Background & Summary

Soil phosphorus (P) availability limits crop yields in many regions of the World^1,2^, while anthropogenic application of P in excess triggers aquatic eutrophication in other regions^3^. While P in unmanaged soils can be predicted to some degree from local soil properties and climate^4–6^, this approach cannot be used for agricultural systems because humans have altered the P cycle in these ecosystems. E.g. the change in soil P input/output related to farming practices has modified the spatial distribution of P in soils^7–9^ with long-lasting legacy effects of past management^10^. Nevertheless, an analysis of the relationships between crop productivity and soil P availability or between P losses and soil P on regional to global scale requires a spatially explicit agricultural soil P dataset. At the global scale, such a dataset has only recently been developed by Ringeval *et al*.^11^ and Zhang *et al*.^12^.

Both Ringeval *et al*.^11^ and Zhang *et al*.^12^ combined datasets describing the different drivers of the global spatial distribution of soil P with a process-based model of soil P dynamics to reconstruct the past and present distributions of P in agricultural soils. Hereafter, the term “drivers” encompasses: variables related to soil P input and output (farming practices, deposition, erosion, sludge), land-use change, variables that have an effect on soil P dynamics (soil properties, climate), and biogeochemical background (i.e. the initial soil P content at the time of conversion to cropland). The soil P dynamics model was used to reconstruct how the different soil P pools characterized by different bio-availabilities evolved in time from the biogeochemical background following i) soil P input/output directly (e.g. farming practices) or indirectly (land-use change) derived from some drivers and ii) as function of some drivers that modulate the fluxes between soil P pools (e.g. climate).

Refs. ^11,12^ used similar datasets to describe the main drivers. In particular, both used the estimates by Bouwman *et al*.^13^ for soil P input/output corresponding to farming practices, and unmanaged soil P data by Yang *et al*.^4^ to approach the biogeochemical background. These dataset have some limitations: e.g. the soil P input/output in grassland provided by Bouwman *et al*.^13^ was poorly described with the plant uptake forced to be equal to a constant fraction of soil P input. In the work of Yang *et al*.^4^ how the total soil P is distributed among different soil pools was only a function of soil order while it is now known that the soil order is a poor predictor^14^. The representation of soil P pools and fluxes between these pools differed between Ringeval *et al*.^11^ and Zhang *et al*.^12^, reflecting the gaps in our understanding of the soil P dynamics and data to constrain process-based models. Zhang *et al*.^12^ simulated only two soil pools (one labile and one stable pool) with poor representation of soil P dynamics, whereas Ringeval *et al*.^11^ used a 6-pool P model based on Hedley fractionation method. Despite a more apparent mechanistic representation of soil P transformation, the model used byRingeval *et al*.^11^ was based on a very limited knowledge about soil P dynamics: e.g. the soil P buffering capacity was represented as function of soil order only and was poorly constrained by measurements^15^.

Because of the above-mentioned limitations, soil P maps simulated by both Ringeval *et al*.^11^ and Zhang *et al*.^12^ had low confidence and none of these studies really investigated the realism of the spatial distribution of the soil P simulated: Zhang *et al*.^12^ focused on the P plant uptake while Ringeval *et al*.^11^ assessed the contribution of each driver to the spatial variation of soil P. The simulated soil P maps were not evaluated and this was in partly explained by missing large-scale datasets of P measurements at that time. Since the initial release in 2017, the knowledge about soil P dynamics has improved, datasets representing some drivers of soil P have been updated, and data on measured soil labile P in agricultural soils started to be available, offering the opportunities to generate more reliable global soil P maps. In particular, the comparison between different measurements (isotopic exchange kinetics and Hedley fractionation) improves our understanding of the forms extracted^16,17^ and our ability to represent the fluxes between these pools^18^. Also, parameterizations of exchanges between inorganic P pools have been improved by considering the effects of soil properties on these fluxes^18^. Dataset used to describe some drivers has been also improved. In particular, both total soil P and its fractions within different pools have been improved by including more predictors and more sites to train the machine learning algorithms in these studies, improving our understanding of variables driving soil P distributions in unmanaged soils^6^. Finally, regional datasets on measured soil available P based on soil monitoring networks are now available (e.g. LUCAS database^19^).

Here we updated the approach described in Ringeval *et al*.^11^ with the aim to improve the global soil P dataset in five ways: i) use new theoretical understanding of inorganic soil P dynamics, ii) use new and better constrained datasets to describe the different drivers, iii) benchmark the new soil P maps with available available global and regional estimates, iv) extend the period of simulations over time (simulations ends in 2005 in Ringeval *et al*.^11^), v) clarify the different assumptions used throughout all steps of our approach and v) make the whole approach (i.e. scripts to download the datasets, scripts to generate the input from these datasets, soil P dynamic model) available to the community. Points iv) and v) would make further updates easier.

The update of Ringeval *et al*.^11^ described in this article provides the simulated size of different soil P pools for the period 1900–2018, with a spatial resolution of 0.5° for both croplands and grasslands. We also provided estimates of the uncertainty related to the description of the different drivers. Spatially gridded estimates of soil P pools will be useful to quantify the current limitation of agricultural production by P and to inform assessments of strategies aiming at reduced fertilizer input by making better use of P present in soils.

## Method

### General approach

Similar to Ringeval *et al*.^11^, the approach used here modelled the global distribution of soil P in croplands and grasslands, with a 0.5° × 0.5° spatial resolution. No vertical discretization was considered as the approach considers only one soil layer, which is the top 0–0.3 m soil layer. For each grid-cell, soil P was distinguished into four land-cover type fractions: (i) cropland (*crop*), (ii) grassland (*grass*) defined as managed pasture + rangeland in Chini *et al*.^20^, (iii) non-agricultural vegetation (*nonagri*), and (iv) urban land (*urban*). While the soil P of the last two land-cover fractions were not explicitly simulated (see below), their consideration was necessary to account for the effect of the land-use and cover change on the cropland/grassland soil P. Land-use and cover change and soil P input/output were prescribed at yearly time-step while soil P dynamics work at daily time-step. Simulations cover the 1900–2018 period.

Our modelling approach combined several global datasets describing the drivers of agricultural soil P with a soil P dynamics model to simulate the temporal evolution of the agricultural soil P from the beginning of the 20th century to the present time period. Drivers are named with four capital letters hereafter. Starting from the soil biogeochemical background used as initial conditions for the year 1900 (BIOG), our modelling approach compute the temporal evolution of soil P pools for cropland and grassland by, i) computing annually the effect of land-use and land-cover change (LUCC), ii) considering the annual soil P input/output corresponding to different drivers (chemical fertilizer, manure, plant uptake and plant residues, encompassed under the term “farming practices”, FARM; input from atmospheric deposition, DEPO; P losses through water erosion, LOSS, P input from sludges, SLUD), iii) simulating at daily time-step the soil P dynamics (i.e. how the different soil P pools exchanges together). Fluxes between soil P forms depends on two drivers: near-surface air temperature and soil temperature/water content (CLIM), and soil properties (SPRO) In total, eight drivers were considered (Tables 1–8). The datasets used to represent the different drivers resulted from the combination of different measurements (satellites, on site measurements, etc.) and/or model simulations. All data sets were regridded to a half-degree resolution.

**Table 1:** FARM driver and its representation in (data = GPASOIL-v0) and (data = GPASOIL-v1).

Driver name	FARM (farming practices)
Description of its effect on the soil P	Soil P input/output related to farming practices
Corresponding variables in our approach	Soil P input/output corresponding to: chemical fertilizer ($f{P}_{chem}^{out\to i-lab}$), manure reaching cropland/grassland soils ($f{P}_{manu}^{out\to m}$ with *m* in *{i-lab, o-lab, o-sta}*), crop/grass residues ($f{P}_{resi}^{out\to m}$ with *m* in *{i-lab, o-lab, o-sta}*), and plant uptake ($f{P}_{upta}^{i-lab\to out}$). Here, ‘residues’ refer to plant biomass that remains on/within the soil after the harvest (for cropland) or harvest/grazing (for grassland). It includes root biomass.
Dataset ref used in (data = GPASOIL-v0)	Chemical fertilizer and manure were taken from^ Bouwman *et al*.13^. Residues and plant uptake were derived from P harvest/withdrawal provided by Bouwman *et al*.^13^ following basic assumptions.
Issues related to the use of this dataset in GPASOIL-v0	The approach used in Bouwman *et al*.^13^ (based on IMAGE^67^,) excluded ~20% of cropland area (due to some boolean treatment about land-use). This lead to some inconsistency with LUCC in GPASOIL-v0. In Bouwman *et al*.^13^, the treatment in grassland was simple: P withdrawal was estimated as ~90% of the total applied fertilizer. In GPASOIL-v0, we used few global numbers, without crop distinction, to derive uptake and residues from withdrawal/harvest (see Supp.Inf of Ringeval *et al*.^11^).
Representation in (data = GPASOIL-v1)	For both cropland and grassland, P in manure reaching the soil was derived from a combination between N in manure reaching grassland soil given by Xu *et al*.^31^, a global P:N ratio (0.20), and P manure produced at country-scale based on livestock category population and manure production rate^9^. Grassland: P in chemical fertilizer was derived from N in chemical fertilizer applied to grassland given by Xu *et al*.^31^ and a global P:N ratio (0.22). P plant uptake was derived from the actual NPP (including above and belowground NPP) in grassland and some spatially constant parameters (P concentration, etc.). P in residues was derived from P plant uptake and (1-GI) with GI: grazing intensity given by Kastner *et al*.^41^ and corresponding to the ratio of the Human appropriation of net primary production through harvest and the actual NPP. The spatial distribution of NPP provided by Kastner *et al*.^41^ was used but global average NPP was made varying between the values of Kastner *et al*.^41^ and Wang *et al*.^42^. Cropland: P in chemical fertilizer was provided by Lu and Tian^34^. The latter ref. ^34^ assumed that all chemical fertilizer is applied to cropland and here, we corrected the chemical fertilizer applied to cropland by subtracting the chemical fertilizer applied on grassland described above. P uptake and P in residues were computed as functions of yield and crop specific parameters. Crop-dependent, spatially explicit and temporal varying yields are get by combining spatially-explicit yield per crop for year 2000 by Monfreda *et al*.^39^ and country-averaged yield (but temporal-varying) given by FAOSTAT. The yield of FAOSTAT at country-scale is extended for years before 1961 by scaling the 1961 yield with the country-sale human population provided by HYDE 3.2^23^. Yield is then combined with different crop dependent parameters (concentration of P in different organs -root, harvest, aboveground biomass excluding the harvest, root:shoot ratio and harvest index) and fraction of total residus remaining on/within soil (equal to 1/2 or 0, following Smil^44^).
Characteristics of the spatially explicit dataset used in (data = GPASOIL-v1)	Xu *et al*.^31^ provided for grassland, chemical N fertilizer for years between 1961 and 2016, and N in manure for years between 1860 and 2016, both in kgN.km^−2^.yr^−1^, and at half-degree spatial resolution^68^ (10.1594/PANGAEA.892940). Demay *et al*.^9^ provided P manure produced at country-scale per livestock category for years between 1950 and 2017 (^69^10.57745/LEPJCS). Kastner *et al*.^41^ provided NPP (in gC m^−2^ yr^−1^) and GI (no unit) for grassland for some years (1910, 1930, 1950, 1960, 1970, 1980, 1990, 2000, 2010) at 0.083° spatial resolution(^70^10.5281/zenodo.7313791). ^Lu and Tian34^ provided chemical P fertilizer applied on cropland for any years between 1900 and 2013, in gP m^−2^ yr^−1^, at half-degree spatial resolution, (^71^10.1594/PANGAEA.863323). Zhang *et al*.^30^ provided N in manure applied on cropland for any years between 1860 and 2014, in in kgN.km^−2^.yr^−1^ at half-degree spatial resolution (^72^10.1594/PANGAEA.871980). Monfreda *et al*.^39^ provided crop-dependent and spatially explicit (half-degree resolution) yield for years ~2000 (Earthstat dataset, http://www.earthstat.org/harvested-area-yield-175-crops/). FAOSTAT provided country-averaged and crop-dependent yield for any years over 1961–2019 (FAOSTAT. License: CC BY-NC-SA 3.0 IGO. Extracted from: https://www.fao.org/faostat/en/#data/QCL and https://www.fao.org/faostat/en/#definitions Data of Access: 20-01-2023.). Klein Goldewijk^23^ provided the country-sale human population (HYDE 3.2^73^, 10.17026/dans-25g-gez3).

**Table 2:** DEPO driver and its representation in (data = GPASOIL-v0) and (data = GPASOIL-v1).

Driver name	DEPO (P in atmospheric deposition)
Description of its effect on the soil P	Soil P input resulting from deposition of atmospheric P
Corresponding variables in our approach	P deposition falling within labile ($f{P}_{depo}^{out\to i-lab}$) and apatite ($f{P}_{depo}^{out\to i-prim}$) soil P pools
Dataset ref used in (data = GPASOIL-v0)	P deposition falling within labile and apatite soil P pools were derived from the Wang *et al*.^49^ dataset that provided the atmospheric P deposition resulting from mineral dust, primary biogenic aerosol particles, sea salt, natural combustion and anthropogenic combustion, averaged over different time-periods. Year-to-year variability of P deposition from anthropogenic combustion was computed for information about emissions. Following Mahowald *et al*.^50^, we considered that 10% of P deposition from dust and 50% of deposition from other sources falls within P_i-lab_ while rest falls within P_i-prim_.
Issues related to the use of this dataset in GPASOIL-v0	None
Representation in (data = GPASOIL-v1)	P deposition falling within labile and apatite soil P pools were derived from the combination of the dataset of Wang *et al*.^49^ and the one provided by Wang *et al*.^51^. For year before 2007, the same approach as in GPASOIL-v0 was used while the dataset of Wang *et al*.^51^ was used to compute the year-to-year variability of P deposition from anthropogenic combustion after the year 2007. The estimates of Wang *et al*.^51^ were corrected to ensure equality with Wang *et al*.^49^ for the years in common (1997–2007). See Eqs. 54, 55 and Text S4.
Characteristics of the spatially explicit dataset used in (data = GPASOIL-v1)	Estimates corresponding to Wang *et al*.^49,51^ were provided by Rong Wang, pers.comm (2020). The monthly estimates of Wang *et al*.^49^ were representative to different time-periods between 1960 and 2011, depending on the variables considered (dust deposition, etc.). Total deposition at monthly time-scale was provided for any years between 1997 and 2013 in Wang *et al*.^51^. The different estimates of deposition are spatially explicit without land-use (cropland, grassland, other) distinction. Emissions related to natural and anthropogenic combustion were provided at annual time-scale for any years between 1960 and 2007 for big World regions in Wang *et al*.^49^.

**Table 3:** SLUD driver and its representation in (data = GPASOIL-v0) and (data = GPASOIL-v1).

Driver name	SLUD (P in sludges)
Description of its effect on the soil P	P in sludges from sewage treatment that are spread on cropland soils
Corresponding variables in our approach	$f{P}_{slud}^{out\to m}$ with *m* in *{i-lab, o-lab, o-sta}*),
Dataset ref used in (data = GPASOIL-v0)	Not considered in Ringeval *et al*.^11^
Issues related to the use of this dataset in GPASOIL-v0	
Representation in (data = GPASOIL-v1)	$f{P}_{slud}^{out\to x-tot}$ was estimated by combining human P excretions with the fraction of sewage sludge that is treated and the P removal efficiency of treatments plants following van Puijenbroek *et al*.^52^ and Demay *et al.*^9^. Human excretions were computed by using excretion rates and human population. Computations were performed at country-scale then all crop within the same country receive the same P (in kgP ha^−1^). For the composition of P in sludges, we used the same labile vs stable contribution as for manure. See Eq. 56 and Text S5.
Characteristics of the spatially explicit dataset used in (data = GPASOIL-v1)	Human P excretion rates for big World regions, as well as fraction of sewage sludge that is treated, both at country scale, were provided by van Puijenbroek *et al*.^52^. Human P excretion rates were available for the years 1970 and 2010 while the fraction of sewage sludge that is treated was available for years 1990, 2000, 2010. Human population was provided by HYDE 3.2^23^ and *Area* at country scale was computed from the dataset used in LUCC (Table 5). The corresponding between big World regions and iso country code was provided by the IMAGE framework region classification (https://models.pbl.nl/image/index.php/Region_classification_map).

**Table 4:** LOSS driver and its representation in (data = GPASOIL-v0) and (data = GPASOIL-v1).

Driver name	LOSS (P losses through soil erosion)
Description of its effect on the soil P	Losses of P due to water erosion and runoff processes
Corresponding variables in our approach	Losses of P for each soil P pool ($f{P}_{loss}^{m\to out}$ with *m* in {i-lab, o-lab, o-sta, i-prim, i-sec, x-occ}).
Dataset ref used in (data = GPASOIL-v0)	Losses were computed using the soil P content simulated in GPASOIL-v0, the sediment gross fluxes resulting from erosion provided by van Oost *et al*.^74^ and the weight of top 0–0.3 m soil computed from bulk density of the same horizon provided by Soilgrids database (ISRIC – World Soil Information, 2016)
Issues related to the use of this dataset in GPASOIL-v0	We did not consider temporal change in the sediment gross fluxes resulting from erosion (but we considered historical changes in agricultural land area, see LUCC)). Also, gross (and not net) erosion rates were considered. Finally, we considered that the erosion rates concerns exclusively the top soil layer (top 0–0.3 m).
Representation in (data = GPASOIL-v1)	The same approach as used in (data = GPASOIL-v0) was used. Sediment gross fluxes resulting from erosion with a cropland/grassland distinction were computed following the approach described in Borrelli *et al*.^54^ Losses through erosion of P in soil solution (P_i-solu_) was set to 0. We made the same assumptions as done in GPASOIL-v0: no temporal change in the gross rate of erosion, focus on gross (instead of net) erosion rate, effect of erosion exclusively on the top layer considered in our study. See Eqs. 57, 58.
Characteristics of the spatially explicit dataset used in (data = GPASOIL-v1)	Dataset were provided by Pasquale Borrelli, pers.comm, (2020) and corresponded to the use of the approach described in Borrelli *et al*.^54^ here applied to the land-use dataset described in LUCC for year 2000 to simulate the gross soil losses (in (kg of soil) yr^−1^ ha^−1^) through erosion with a cropland/grassland distinction at 250mx250m resolution. Bulk density and volumetric fraction of coarse fragment were provided by Soilgrids 2.0^21^ at half-degree resolution.

**Table 5:** LUCC driver and its representation in (data = GPASOIL-v0) and (data = GPASOIL-v1).

Driver name	LUCC (land use and land cover change)
Description of its effect on the soil P	Decrease/increase of agricultural soil P resulting from land conversion within the same grid-cell (see Eq. 18).
Corresponding variables in our approach	Fractions of cropland ($frac\left(y,crop,g\right)$) and of grassland ($frac\left(y,grass,g\right)$) for any year y and grid-cell g as well as transitions between the 4 land-use categories considered in our study (e.g. ${\Delta }_{i}\left(y,j,g\right)$ is the conversion from *j* to *i* with both *i* and *j* in {crop, grass, nonagri, urban}).
Dataset ref used in (data = GPASOIL-v0)	Fractions and transitions were provided by the Land Use Harmonization dataset^75^
Issues related to the use of this dataset in GPASOIL-v0	
Representation in (data = GPASOIL-v1)	Fractions and transitions were provided by Chini *et al*.^20^, the update of the Land Use Harmonization dataset (the so-called LUH2-GCB2019 dataset). We made a corresponding between our land-use categories and Chini *et al*.^20^ categories. See Eqs. 59–62
Characteristics of the spatially explicit dataset used in (data = GPASOIL-v1)	Chini *et al*.^20^ provided LUH2-GCB2019 (10.3334/ORNLDAAC/1851^76^), an update of the Land Use Harmonization dataset used in GPASOIL-v0. The LUH2-GCB2019 dataset provided fractions and transitions between crops (C3/C4, annual/perennial, C3-nitrogen fixing), grassland (managed pasture, rangeland), forest and urban land, at 0.25°x0.25° spatial resolution for any years between 850 and 2018.

**Table 6:** CLIM driver and its representation in (data = GPASOIL-v0) and (data = GPASOIL-v1).

Driver name	CLIM (near-surface air temperature, soil temperature and soil water content)
Description of its effect on the soil P	Few effects can be distinguished: - Effect of soil temperature and relative soil water content on P weathering and P mineralization in both (model = GPASOIL-v0) and (model = GPASOIL-v1) (See Eqs. 9–11) - Near-surface atmospheric temperature and soil water content involved in parameterizations of (model = GPASOIL-v1) (see Table 10) - Soil water content used to translate P_i-sol_ into soil solution P concentration in (model = GPASOIL-v1) (see e.g. Equations 3 and 6)
Corresponding variables in our approach	Relative soil liquid water content (W_rel_, in fraction of field capacity) or soil liquid water content (W_abs_, in L(kg of soil)^−1^) soil temperature (T_soil_, in °C), T_a_: near-surface atmospheric temperature (in °C). W_rel_, W_abs_, T_soil_ are representative to the 0–0.3 m soil layer.
Dataset ref used in (data = GPASOIL-v0)	The variables were computed by averaging corresponding variables simulated by two Dynamic Global Vegetation Models (ISBA^77^ and ORCHIDEE^24^). The annual average of the climatology computed for the 1979–2010 period (i.e. no year-to-year variability) was used.
Issues related to the use of this dataset in GPASOIL-v0	Only two Dynamic Global Vegetation Models were considered. No temporal change was considered (the 1970–2010 climatology was used instead).
Representation in (data = GPASOIL-v1)	We used the average among 9 simulations (combination between 4 land-surface models and 3 climate data used as input of the land-surface models for the historical period) performed for the CMIP-6 exercise. These simulations have been considered as they provide the variables at the basis of our computation of W_rel_, W_abs_ and T_soil_. For each variable, we computed the annual averages over the 1850–2012 period. Variables used are representative to the top 0–0.3 m soil layer. Only liquid content was considered to compute W_rel_ and W_abs_. T_a_ is given by forcing files used as input of the land-surface models.
Characteristics of the spatially explicit dataset used in (data = GPASOIL-v1)	Original resolution and time-period considered varied among the 9 simulations considered. The link (with doi) of each simulation is provided below: CNRM-ESM2-1 x land-hist: 10.22033/ESGF/CMIP6.9599 CNRM-ESM2-1 x land-hist-cruNcep: 10.22033/ESGF/CMIP6.9600 CNRM-ESM2-1 x land-hist-princeton: 10.22033/ESGF/CMIP6.9601 CNRM-CM6-1 x land-hist: 10.22033/ESGF/CMIP6.4095 CNRM-CM6-1 x land-hist-cruNcep: 10.22033/ESGF/CMIP6.4100 CNRM-CM6-1 x land-hist-princeton: 10.22033/ESGF/CMIP6.4101 IPSL-CM6A-LR x land-hist: 10.22033/ESGF/CMIP6.5205 MIROC6 x land-hist-cruNcep: 10.22033/ESGF/CMIP6.5627 MIROC6 x land-hist-princeton: 10.22033/ESGF/CMIP6.5628

**Table 7:** SPRO driver and its representation in (data = GPASOIL-v0) and (data = GPASOIL-v1).

Driver name	SPRO (soil properties)
Description of its effect on the soil P	Soil properties involved in the computation of the soil P dynamics
Corresponding variables in our approach	Variables considered within the SPRO drivers vary between GPASOIL-v0 and GPASOIL-v1: - in (model = GPASOIL-v0), SPRO encompasses parameters involved in the Langmuir equation used to describe the equilibrium between P_i-lab_ and P_i-sec_ (so-called K_S_ and S_max_ parameters) - in (model = GPASOIL-v1), SPRO encompasses soil properties involved in parameterizations for soil P dynamics: soil texture (sand, clay, silt percentages: s_s_, s_c_, s_i_ respectively, in %), soil water pH (pH, no unit), C is soil carbon concentration (in gC.(kg of soil)^−1^)
Dataset ref used in (data = GPASOIL-v0)	A coupled of values for (K_S_, S_max_) was used for each soil order. These values were provided by Wang *et al*.^15^. The global distribution of soil orders was similar to the one used in Yang *et al*.^4^.
Issues related to the use of this dataset in GPASOIL-v0	Soil orders are likely a poor predictor of soil P buffering capacity in (model = GPASOIL-v0). Other properties (texture, pH, carbon) were not used in (data = GPASOIL-v0).
Representation in (data = GPASOIL-v1)	K_S_, S_max_ are not used any more in (model = GPASOIL-v1). Soil texture, soil water pH, and soil carbon concentration for top 0.3 m were get from Soilgrids 2.0^21^. We assumed that that soil properties at half-degree resolution could be applied to the cropland/grassland (soils) fraction. Soil properties were involved in equations described in Table 10.
Characteristics of the spatially explicit dataset used in (data = GPASOIL-v1)	Soil texture, soil water pH, and soil carbon concentration for top 0.3 m were computed by averaging values for 0–0.05, 0.05–0.15 and 0.15–0.30 m soil layers provided by Soilgrids 2.0^21^. The Soilgrids procedure allows to download the data at the resolution needed here (half degree spatial resolution).

**Table 8:** BIOG driver and its representation in (data = GPASOIL-v0) and (data = GPASOIL-v1).

Driver name	BIOG (natural soil biogeochemical background)
Description of its effect on the soil P	P inherited from natural soils at the time of conversion to agriculture (Eq. 20). P in natural soils was also used to approach P in agricultural soils at the beginning of the simulation (initial conditions, Eqs. 22, 23) and soil P pools at steady-state used to compute the parameter describing the exchanges between pools (Eq. 17).
Corresponding variables in our approach	P content of natural soils for any soil P pools considered, i.e. ${P}_{i-sol}^{NA}$, ${P}_{i-lab}^{NA}$, ${P}_{o-lab}^{NA}$, ${P}_{o-sta}^{NA}$, ${P}_{i-prim}^{NA}$, ${P}_{i-sec}^{NA}$, ${P}_{i-occ}^{NA}$, in kgP ha^−1^ for top 0–0.3 m.
Dataset ref used in (data = GPASOIL-v0)	Natural soils P pools for top 0–0.3 m were approached by estimates of current P in unmanaged soils for top 0–0.5 m provided by Yang *et al*.^4^. P_i-sol_ was not considered in GPASOIL-v0.
Issues related to the use of this dataset in GPASOIL-v0	Soil orders used to compute how total P is held in different fractions in Yang *et al*.^4^ is likely a poor predictor of soil P pools. In our approach, we assumed that P concentration provided for top 0–0.5 m was representative to the considered top 0–0.3 m soil layer.
Representation in (data = GPASOIL-v1)	We used the dataset of He *et al*.^14^ that provides the current soil P distribution for ${P}_{i-lab}^{NA}$, ${P}_{i-sec}^{NA}$, ${P}_{i-prim}^{NA}$, ${P}_{x-occ}^{NA}$, ${P}_{o-sta}^{NA}$, ${P}_{o-sta}^{NA}$. The values we used are representative to the top 0 - 0.3 m soil layer. ${P}_{i-sol}^{NA}$ is derived from ${P}_{c,\infty }$ that was prescribed to 0.1 mgP L^−1^ (but sensitivity to this value was tested).
Characteristics of the spatially explicit dataset used in (data = GPASOIL-v1)	^He *et al*.14^ provided the current soil P pools distribution (in mgP (kg of soil)^−1^) for different soil horizons at half-degree spatial resolution for natural ecosystems (^78^, 10.6084/m9.figshare.16988029.v2).

Independent of any driver, three other soil properties were used in our approach to perform change in unit, namely the bulk density of the fine earth fraction, the volumetric fraction of coarse fragments ( > 2 mm) and the soil depth. These three variables were either used to convert concentration (i.e. per kg of soil) into quantity per soil surface for both P or water, or to compute soil eroded in fraction of soil mass lost per year. In particular, soil P input/output were mainly given in the different dataset in kgP ha^−1^ yr^−1^ and soil P dynamics worked with P concentration in mgP (kg soil)^−1^. Given the scarcity of dataset about the thickness of the plough layer at the global scale, we considered a globally uniform thickness of 0.3 m as in Ringeval *et al*.^11^. This soil layer was also considered as encompassing a major proportion of crop roots. The soil bulk density at 0.5° latitude and longitude resolution from Soilgrids 2.0^21^ was used to approach the density of cropland/grassland of each grid-cell given the unavailability of global datasets focusing on agricultural soils, even though it is known that soil treatment has an effect on soil physical properties^22^. Same reasoning applied to the fraction of coarse fragments, also provided by Soilgrids 2.0. The uncertainty associated to the bulk density and fraction of coarse fragments was not considered in our study as we restricted the uncertainty analysis to the one of the drivers. In the present study, all P pools were finally expressed in kgP ha^−1^ for top 0–0.3 m soil layer. Large-scale soil P pools are computed by using cropland and grassland fraction (see LUCC driver below) and grid-cell land area (computed from regriding the land fraction provided by HYDE 3.2^23^, 10.17026/dans-25g-gez3).

The new estimates of the soil P distribution in cropland and grasslands are called GPASOIL-v1 and we used the name GPASOIL-v0 to describe the estimates calculated in the previous study of Ringeval *et al*.^11^. Estimates result from a coupling between dataset describing drivers (called “data” in the following) and a soil P dynamics model (called “model” hereafter), and v0 or v1 can be attributed to each component (data or model) as each one has been updated from the study of Ringeval *et al*.^11^ to the current one.

### Soil P dynamics model

#### Pools and fluxes design

**Fig. 1:**
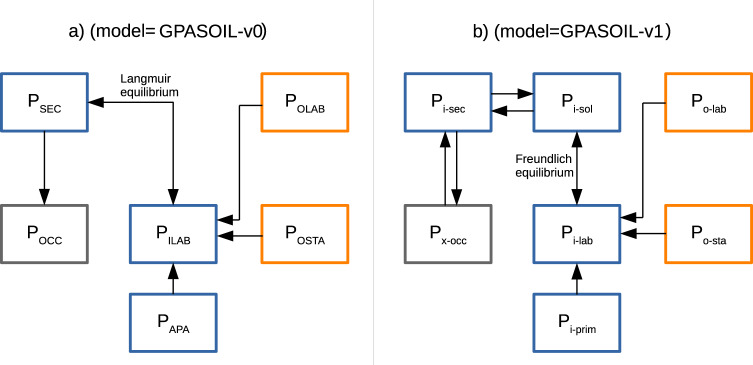
Difference in design between soil P pool dynamics model used in this study (model = GPASOIL-v1, panel (**b**)) and used in Ringeval *et al*.^11^ (model = GPASOIL-v0, panel (**a**)). Inorganic pools are in blue, organic ones are in orange and grey pools correspond to pools which encompass both inorganic and organic P forms. Double arrows means that an equilibrium is considered. Nomenclature used to name the pools changed between (model = GPASOIL-v0) and (model = GPASOIL-v1).

The soil P pools considered in (model = GPASOIL-v1) were named with the following nomenclature: P_a-b_ with *a* in {*i,o,x*} (*i* for inorganic, *o* for organic and *x* for inorganic + organifc (i + o)); and *b* referring to different types of soil P pools. In total, 7 pools were considered following the merging of Hedley fractions (Table 9, Fig. 1b): P_i-sol_ (inorganic P in soil solution), P_i-lab_ (labile inorganic P), P_i-sec_ (moderately labile inorganic P), P_i-prim_ (primary inorganic P), P_o-lab_ (labile organic P), P_o-sta_ (stable organic P), P_x-occ_ (occluded P). P_x-tot_ is the sum of all pools considered.

**Table 9:** Corresponding between pools name and Hedley fractions in (model = GPASOIL-v0) and (model = GPASOIL-v1).

Pools name used in (model = GPASOIL-v0)	Pools definition used in (model = GPASOIL-v0)	Hedley fractions considered on sites used in Ringeval *et al*.^11^	Pools name used in (model = GPASOIL-v1)	Pools definition used in (model = GPASOIL-v1) (following He *et al*.^14^)	Corresponding with Hedley fraction used in He *et al*.^14^
P_ILAB_	Labile inorganic P	[H_2_O Pi/Resin Pi+] Bicarbonate Pi	P_i-solu_	Inorganic P in soil solution	H_2_O Pi/Resin Pi + Bicarbonate Pi
			P_i-lab_	Labile inorganic P	
P_SEC_	Inorganic P bound on secondary minerals	Hydroxide Pi [+Sonic Pi]	P_i-sec_	Moderately inorganic P	Hydroxide Pi
P_OLAB_	Labile organic P	[H_2_O Po + Resin Po+] Bicarbonate Po	P_o-lab_	Labile organic P	Bicarbonate Po
P_OSTA_	Stable organic P	Hydroxide Po [+Sonic Po + HCl Po]	P_o-sta_	Moderately organic P	Hydroxide Po
P_APA_	Apatite	HCl Pi (HCl diluated or not)	P_i-prim_	Primary inorganic P	HCl Pi (HCl diluated only)
P_OCC_	Occluded inorganic P	Residual P [+Hot HCl]	P_x-occ_	Occluded P (inorganic + organic)	Residual P [+Hot HCl P + Sonic P]

Squared brackets in columns 3 and 6 are used for Hedley fractions which are not systematically quantified. Columns 2 and 3 were adapted from the Table 2 of Yang and Post^79^. Please, note that all occluded P was considered as inorganic P in Ringeval *et al*.^11^ while this was not stated in Yang and Post^79^.

Hereafter, the following nomenclature was used to name the flux mediated by the process *“proc”* from pool m*1* to m*2*: $f{P}_{proc}^{m1\to m2}$. The superscripts m1 and m2 correspond to soil P pools in case of soil P dynamics or could be used to describe soil P input/output (« out » for outside is used in that case). The name “proc” was used to describe the process that lead to the flux considered and either referred to a soil process (“occl” for occlusion, “deoccl” for deocclusion, “sorp” for sorption, “desorp” for desorption, “weat” for weathering, “mine” for mineralization), or to a soil P input/output (“chem” for chemical fertilizer, “manu” for manure, “resi” for plant residues, “upta” for plant uptake, “loss” for losses through water erosion, “depo” for atmospheric deposition, “slud” for sludges from waste treatment). As examples, $f{\,P}_{mine}^{o-sta\to i-lab}$ corresponded to the mineralization of P_o-sta_ to P_i-lab_ while $f{P}_{chem}^{out\to i-lab}$ corresponded to soil P input of chemical fertilizer to P_i-lab_. $f{P}_{lucc}$ was used to name the effect of land-use change on soil P (section “Effect of land-use and land-cover change (LUCC) on cropland/grassland soil P”).

In (model = GPASOIL-v1), the equations describing soil inorganic P dynamics between P_i-sec_, P_i-lab_, P_x-occ_, P_i-sol_ were given by Wang *et al*.^18^. Weathering and mineralization were simulated as in (model = GPASOIL-v0) but other mineralization rates values were here tested (see section “Parameter estimates”). In the present section, fP was expressed in mgP (kg soil)^−1^ day^−1^, soil P pools (P_i-sol_, P_i-sec_, etc.) in mgP (kg soil)^−1^ and soil water content (W_abs_) in L (kg of soil)^−1^ to make parameters unit consistent to the units used in Wang *et al*.^18^ (Table 10). The different unit conversions were done by using the bulk density (in kg m^−3^), volumetric fraction of coarse fragments (no unit) and spatially constant soil depth (0.3 m). Fluxes and pools varied as functions of year (y), land-cover (lu, either equal to crop or grass) and space (g, for grid-cell) (i.e. fP = fP(y,lu,g)). Parameters involved in Eqs. 1–8 (e.g. ${k}^{i-sec\to x-occ}$ and *b*) varied as a function of grid-cell only and W_abs_ as a function of year and grid-cell; but for the sake of readability, we omit these in the below equations.

**Table 10:** Original parametrizations provided by Wang *et al*.^18^ and modified parametrizations used in this study after i) exclusion of oxalate concentrations from explanatory variablex and ii) setting *k* parameters as constant in time.

Parameters	Name in Wang *et al*.^18^	unit		Equation	r²
${P}_{c,\infty }$	${C}_{\infty }$	mgP L^−1^	^18^	${P}_{c,\infty }=1{0}^{\left[-1.7425+1.7782\left(1-exp\left(-1.6653{C}_{X}\right)\right)\right]}$	0.54
			This study	${P}_{c,\infty }=0.1$	
b	b	dimensionless	^18^	$b=exp\left(0.532-0.13{O}_{x}-37.49{f}_{i-sol}+0.81{f}_{i-lab}+0.003{s}_{s}\right)$	0.43
			This study	$b=exp\left(-0.628-36.702{f}_{i-sol}+1.102{f}_{i-lab}+0.0024{s}_{s}\right)$	0.23
${k}^{i-lab\to i-sol}$	k_WL_	day^−1^	^18^	${k}^{i-lab\to i-sol}=-4.82+209{f}_{i-sol}+14.64{f}_{x-occ}+9.26{f}_{i-sec}-0.008C-0.0003{P}_{i-tot{\rm{\backslash prim}}}-0.018{s}_{{\rm{i}}}$	0.28
			This study	Same as^18^ but use of ${P}_{i-tot{\rm{\backslash prim}},\infty }$ instead of ${P}_{i-tot{\rm{\backslash prim}}}$	
${k}^{i-sol\to i-sec}$	k_SW_	mgP (kg soil)^−1^ day^−1^ (mg P/L)^−b^	^18^	${k}^{i-sol\to i-sec}=exp\left(0.002+4.0{f}_{i-sec}+0.0008{P}_{o-tot}+0.012C+0.108{T}_{a}-0.0002{P}_{i-tot{\rm{\backslash prim}}}\right)$	0.64
			This study	Same as^18^ but use of ${P}_{o-tot,\infty }$ and ${P}_{i-tot{\rm{\backslash prim}},\infty }$ instead of P_*o-tot*_ and ${P}_{i-tot{\rm{\backslash prim}}}$, respectively	
${k}^{i-sec\to x-occ}$	k_OS_	day^−1^	^18^	${k}^{i-sec\to x-occ}=1.6{e}^{-5}+0.0001{f}_{x-occ}-0.0001{f}_{i-sec}+1.3{e}^{-5}pH-9.0{e}^{-6}Ox-4.48{e}^{-7}{s}_{c}+1.1{e}^{-7}C$	0.90
			This study	${k}^{i-sec\to x-occ}=3.68{e}^{-5}+9.60{e}^{-5}{f}_{x-occ}-1.47{e}^{-4}{f}_{i-sec}+1.22{e}^{-5}pH-4.07{e}^{-7}{s}_{c}+5.16{e}^{-8}C$	0.90

All parameterizations are built from a stepwise multivariate linear regression against the database used in Wang *et al*.^18^. r² provided in the last column characterize either the original parametrizations (and thus they are equal to values given in Table 2 of Wang *et al*.^18^) or parametrizations after excluding oxalate from explanatory variables (this study). The parameters *k* were kept constant in time through i) the use of a constant-in-time value for *f*_*m*_ for any soil pool *m* and, ii) the use of ${P}_{o-tot,\infty }$ and ${P}_{i-tot\mathrm{\backslash prim},\infty }$ in the parametrizations instead of ${P}_{o-tot}$ and ${P}_{i-tot\mathrm{\backslash prim}}$, respectively.

Note: C is soil carbon concentration in gC (kg of soil)^−1^, O_x_ is oxalate metal oxide (Al and Fe) concentration in mmol(kg of soil)^−1^, C_x_ is the ratio of total soil C and oxalate metal oxide concentrations in g/mmol (C_x_ = 0.001 C/O_x_), s_s_, s_c_ and s_i_ are sand, clay and silt percentages, respectively, T_a_ is the mean annual near-surface air temperature in °C, pH is soil pH measured in water (no unit), P_o-tot_ is the total soil organic P in mgP (kg of soil)^−1^ and ${P}_{i-tot\mathrm{\backslash prim}}$ is the total inorganic P minus primary P in in mgP (kg of soil)^−1^, f_m_ denotes the ratio (no unit) at steady-state of pool m and ${P}_{i-tot\mathrm{\backslash prim},\infty }$. Subscript ∞ denotes pools at the steady-state.

As in Wang *et al*.^18^, occlusion/deocclusion are given by Eqs. 1, 2 and sorption/desorption between P_*i-sec*_ and P_*i-sol*_ are computed with Eqs. 3, 4:

1 $$f{P}_{occl}^{i-sec\to x-occ}={k}^{i-sec\to x-occ}.{P}_{i-sec}$$

2 $$f{P}_{deoccl}^{x-occ\to i-sec}={k}^{x-occ\to i-sec}.{P}_{x-occ}$$

3 $$f{P}_{sorp}^{i-sol\to i-sec}={k}^{i-sol\to i-sec}.{\left({P}_{i-sol}/{W}_{{\rm{abs}}}\right)}^{b}$$

4 $$f{P}_{desorp}^{i-sec\to i-sol}={k}^{i-sec\to i-sol}.{P}_{i-sec}$$

where P_i-sol_/W_abs_ corresponds to the P concentration in the soil solution (in mgP L^−1^), *k* (in day^−1^) and *b* (unitless) are parameters. Sorption follows a Freundlich equation.

In Wang *et al*.^18^, sorption/desorption fluxes between P_i-sol_ and P_i-lab_ are computed as follows:

5 $$f{P}_{desorp}^{i-lab\to i-sol}={k}^{i-lab\to i-sol}.{P}_{i-lab}$$

6 $$f{P}_{sorp}^{i-sol\to i-lab}={k}^{i-sol\to i-lab}{\left({P}_{i-sol}/W\right)}^{b}$$

To simplify the application of the^18^ model at the global scale in the current study, an equilibrium between P_i-sol_ and P_i-lab_ is assumed at daily time-step, following Wang *et al*.^15^. Thus, ${k}^{i-lab\to i-sol}{P}_{i-lab}={k}^{i-sol\to i-lab}{\left({P}_{i-sol}/{W}_{{\rm{abs}}}\right)}^{b}$ i.e.:

7 $${P}_{i-lab}=\frac{{k}^{i-sol\to i-lab}}{{k}^{i-lab\to i-sol}}{\left({P}_{i-sol}/{W}_{{\rm{abs}}}\right)}^{b}$$

Numerical resolution of Eq. 7 was done by substituting P_i-sol,lab_ = P_i-sol_ + P_i-lab_ in Eq. 7 and rearranging to get:

8 $${k}^{i-sol\to i-lab}{\left({P}_{i-sol}\right)}^{b}+{k}^{i-lab\to i-sol}{\left({W}_{{\rm{abs}}}\right)}^{b}{P}_{i-sol}-{k}^{i-lab\to i-sol}{\left({W}_{{\rm{abs}}}\right)}^{b}{P}_{i-sol,lab}=0$$

where the unique unknown is P_i-sol_. Equation 8 was used to redistribute P_i-sol,lab_ between P_i-sol_ and P_i-lab_ by assuming a steady-state between P_i-sol_ and P_i-lab_. Equation 8 was solved using scipy.optimize.root solver in python3.6.

The Wang *et al*.^18^ model excludes inorganic primary P and organic pools, and their exchanges with other inorganic pools. To model weathering and mineralization in (model = GPASOIL-v1), the same equations as in (model = GPASOIL-v0) were used. The weathering is computed as follows:

9 $$f{P}_{weat}^{i-prim\to i-lab}={k}^{i-prim\to i-lab}\ast {g}_{1}\left({T}_{soil}\right)\ast {g}_{2}\left({W}_{rel}\right)\ast {P}_{i-prim}$$

where g_1_ and g_2_ (unitless) described the sensitivity to soil temperature (T_soil_, in °C) and relative soil water content (W_rel_, unitless), respectively. As detailed in Ringeval *et al*.^11^, g_1_(T) is described with a Q_10_ of 2.4 and a reference temperature of 15 °C and g_2_(W_rel_) = W_rel_. The relative soil water content is defined by:

${W}_{rel}={W}_{{\rm{abs}}}/{W}_{fc}$ with W_fc_ representing the field water capacity (in L (kg of soil)^−1^). Dataset used to get values of W_abs_, W_fc_, T_soil_ are described in the CLIM driver.

Fluxes from organic to inorganic pools (mainly assimilated to mineralization) are given by:

10 $$f{P}_{mine}^{o-sta\to i-lab}={k}^{o-sta\to i-lab}\ast {h}_{1}\left({T}_{soil}\right)\ast {h}_{2}\left({W}_{rel}\right)\ast {P}_{o-sta}$$

11 $$f{P}_{mine}^{o-lab\to i-lab}={k}^{o-lab\to i-lab}\ast {h}_{1}\left({T}_{soil}\right)\ast {h}_{2}\left({W}_{rel}\right)\ast {P}_{o-lab}$$

where h_1_ and h_2_ represent the sensitivity to soil temperature and soil water content and ${k}^{o-sta\to i-lab}$ and ${k}^{o-lab\to i-lab}$ are turnover rates for stable and labile pools (in day^−1^), respectively. The same functions as the ones used in the ORCHIDEE^24^ dynamic global vegetation model to characterize the carbon mineralization were prescribed to h_1_(T_soil_) and h_2_(W_rel_) (i.e. Q_10_ = 2 and reference temperature = 30 °C for h_1_; ${h}_{2}\left({W}_{rel}\right)=-1.1{\left({W}_{rel}\right)}^{2}+2.4{W}_{rel}-0.29$).

At the 1^st^ day of each year, soil P input/output (chemical fertilizer, manure, plant residues, plant uptake, atmospheric deposition, sludges, losses by water erosion) were added/removed to/from the different soil P pools. Each soil input/output had a given composition that defines which pools were enriched or depleted, as provided later. The only exception to this rule was P_i-sol_ which was neither enriched nor depleted by soil P input/output in the 1^st^ day. Any P fluxes that should have reached or leave P_i-sol_ was set to occur with the P_i-lab_ pool instead. For instance, while it is well known that plant only takes up P in soil solution, our modeling approach did not allow to simulate daily plant growth and related P uptake from P in solution. Instead, our methodological choice was to remove annual plant uptake from P_i-lab_ at the 1^st^ day of the year, then to make P_i-lab_ and other soil pools interact for each following day of the rest of the year. Within each time-step (day), the following order of processes was considered: exchanges between pools/forms were first computed, then pools were updated according to these changes and finally, the equilibrium between P_i-sol_ and P_i-lab_ was computed.

To avoid over depletion of a given soil P pool, i.e. the net output flux at the model time step exceeding the pool size, we restricted this flux to the pool size. The effect of this restriction will be evaluated in the section « Technical validation ». After removing $f{P}_{upta}^{i-lab\to out}$ from P_i-lab_ and before computing the equilibrium between P_i-sol_ and P_i-lab_, if (P_i-sol + _P_i-lab_) was negative, (P_i-sol + _P_i-lab_) was set to 0 and the uptake not satisfied by the soil P pools simulated was saved to a variable called $f{P}_{upns}$. This variable corresponded to the part of the uptake which was prescribed to the soil P dynamics model but that the model was not able to sustain. In that case, $f{P}_{upta}^{i-lab\to out}$ was corrected to the maximum uptake that was determined by the size of (P_i-sol + _P_i-lab_).

#### Parameter estimates

Equations 1–8 are based on Wang *et al*.^18^. To estimate the flux parameters *b*, ${k}^{i-lab\to i-sol}$, ${k}^{i-sol\to i-sec}$, and ${k}^{i-sec\to x-occ}$, Wang *et al*.^18^ proceed in two steps by first calibrating these parameters against isotopic exchange kinetics experiments from 147 sites. Then, they regressed such optimized parameters against pedo-climatic variables. They found that *b*, ${k}^{i-lab\to i-sol}$, ${k}^{i-sol\to i-sec}$, ${k}^{i-sec\to x-occ}$ correlate with soil P fractions of different inorganic pools (the term “fraction” is used here to define the ratio between a given pool and the sum of all pools considered in Wang *et al*.^18^, see the variables called *f* in Eqs. 12–14 and definition in Eq. 15), soil organic carbon and oxalate extractable metal oxide concentrations. Parametrizations found in Wang *et al*.^18^ are given in Table 10. Here, in order to estimate these parameters at the global scale at which oxalate extractable metal oxide concentrations is not available, we re-compute the regressions after excluding oxide concentrations from explanatory variables (Table 10).

Wang *et al*.^18^ derived other parameters (${k}^{i-sol\to i-lab}$, ${k}^{i-sec\to i-sol}$, ${k}^{x-occ\to i-sec}$) from relationships with *b*, ${k}^{i-lab\to i-sol}$, ${k}^{i-sol\to i-sec}$, and ${k}^{i-sec\to x-occ}$. These relationships were also used in our study:

12 $$\frac{{k}^{i-sol\to i-lab}}{{k}^{i-lab\to i-sol}}=\frac{{f}_{i-lab}\overline{{W}_{{\rm{abs}}}}}{b{\left({P}_{c,\infty }\right)}^{b-1}{f}_{i-sol}}$$

13 $$\frac{{k}^{i-sol\to i-sec}}{{k}^{i-sec\to i-sol}}=\frac{{f}_{i-sec}\overline{{W}_{{\rm{abs}}}}}{b{\left({P}_{c,\infty }\right)}^{b-1}{f}_{i-sol}}$$

14 $$\frac{{k}^{x-occ\to i-sec}}{{k}^{i-sec\to x-occ}}=\frac{{f}_{i-sec}}{{f}_{x-occ}}$$

where ${P}_{c,\infty }$ is the concentration of P in soil solution at steady-state, overlined W_abs_ means temporal average of W_abs_, f_m_ denotes the ratio (no unit) at steady-state of pool *m* and the sum of inorganic pools considered in Wang *et al*.^18^ called here P_i-tot\prim_, e.g. for m = i-sec,

15 $${f}_{i-sec}=\frac{{P}_{i-sec;\infty }}{{P}_{i-tot\backslash {\rm{prim}};\infty }}=\frac{{P}_{i-sec;\infty }}{{P}_{i-sec;\infty }+{P}_{i-sol;\infty }+{P}_{x-occ;\infty }+{P}_{i-lab;\infty }}$$

with subscript ∞ denotes pools at the steady-state. Note that Wang et al.^18^ made distinctions between measured (f*) and modeled (f) fractions but this distinction does not make sense in this study where fractions are only simulated. Wang *et al*.^18^ also considered W_abs_ equal to 10 L (kg of soil)^−1^ representative to water content of isotopic dilution while we used here the averaged over the whole simulation (1900–2018) of W_abs_. The parameter ${P}_{c,\infty }$ that describes the concentration of P in soil solution at steady-state depends only on oxalate in Wang *et al.*^18^ and we prescribed here an arbitrary constant value:

16 $${P}_{c,\infty }=0.1\;{{\rm{mgPL}}}^{-1}$$

which is equal to the median value of Helfenstein *et al*.^25^. The sensitivity to the value chosen for ${P}_{c,\infty }$ was assessed in the “Technical validation” section. Dataset for calculating soil parameters using the relationships in Table 10 are given in the SPRO driver description (Table 7).

The rate parameters (different *k*) and parameter *b* are only grid-cell dependent and do not differ between cropland and grassland. Following parameterizations found in Wang *et al*.^18^, these parameters could theoretically vary in time with temporal variations of the different soil P fractions at equilibrium (f), organic P (P_*o-tot*_) and the sum of total inorganic pools considered in Wang *et al*.^18^ (i.e. ${P}_{i-tot{\rm{\backslash prim}}}$) (see Table 10). We choose here to keep them constant through i) the use of time-invariant value for ${P}_{m,\infty }$ (and thus for *f*_*m*_) for any soil pool *m* and, ii) the use of ${P}_{o-tot,\infty }$ and ${P}_{i-tot{\rm{\backslash prim}},\infty }$ in Table 10 instead of ${P}_{o-tot}$ and ${P}_{i-tot{\rm{\backslash prim}}}$, respectively. ${P}_{m,\infty }$ is assumed to have the current value of unmanaged soil P pools ${P}_{m}^{NA}$ given by He *et al*.^14^ for the same grid-cell *g*, as follows:

17 $${P}_{m,\infty }\left(g\right)={P}_{m}^{NA}\left(g\right)$$

in a similar way to the computation of initial conditions (see the section about LUCC).

To summarize, the main differences between the original Wang *et al*.^18^ model and the model used here for inorganic soil P dynamics are the following:

- we assumed an equilibrium between P_i-sol_ and P_i-lab_ (Eq. 8) instead of (Eqs. 5-6)

- our parametrization excluded oxalate as explanatory variables (thus simplified equations for *b*, ${k}^{i-lab\to i-sol}$, ${k}^{i-sol\to i-sec}$, and ${k}^{i-sec\to x-occ}$, as well as a constant in space ${P}_{c,\infty }$; Table 10) and we considered that all *k* parameters as constant in time.

The weathering rate (${k}^{i-prim\to i-lab}$_)_ was set to 2.7e-7 day^−1^ following Buendia *et al*.^26^ as in (model = GPASOIL-v0). Regarding mineralization rates, ${k}^{o-sta\to i-lab}$ = 2.7e^−5^day^−1^ and ${k}^{o-lab\to i-lab}$ = 2.7e^−4^day^−1^ were prescribed for organic stable and organic labile pools, i.e. a residence time of 100 and 10 years, respectively, as in (model = GPASOIL-v0). Given the uncertainty related to such rates^27^, another values were tested: ${k}^{o-sta\to i-lab}$ = 1.8e^−4^day^−1^ and ${k}^{o-lab\to i-lab}$ = 1.4e^−3^day^−1^; i.e. a residence time of respectively 15 and 2 yr. Such smaller residence time of those two parameters allow the simulated soil P pool for grassland to become stable during the 1^st^ half of the 20^th^ century (see the “Technical validation” section). (model = GPASOIL-v1.0) was used to name the model with ${k}^{o-sta\to i-lab}$ = 2.7e^−5^day^−1^ and ${k}^{o-lab\to i-lab}$ = 2.7e^−4^day^−1^ while (model = GPASOIL-v1.1) was used for the model with ${k}^{o-sta\to i-lab}$ = 1.8e^−4^day^−1^ and ${k}^{o-lab\to i-lab}$ = 1.4e^−3^day^−1^ (Table 11).

**Table 11:** Name of the soil P estimates used in this study.

Name of the soil P estimates	data	model
GPASOIL-v0^11^	GPASOIL-v0	GPASOIL-v0
Not named in the manuscript	GPASOIL-v1	GPASOIL-v0
GPASOIL-v1.0	GPASOIL-v1	GPASOIL-v1
GPASOIL-v1.1	GPASOIL-v1	GPASOIL-v1.1 corresponds to GPASOIL-v1 with increased mineralization rates

The soil P estimates results from a coupling between dataset describing drivers (“data”) and a soil P dynamics model (“model”), and different combinations (data x model) were tested in the manuscript. “v0” refers to Ringeval *et al*.^11^ while “v1” refers to the update presented in the current draft.

#### Summary of the main differences with (model = GPASOIL-v0)

Soil P pools considered in (model = GPASOIL-v0) were listed in Table 9 and both pools and fluxes considered were plotted in Fig. 1a. Main differences with (model = GPASOIL-v1) were represented in Fig. 1 and listed hereafter:

(i). (model = GPASOIL-v1) considered an additional inorganic P in soil solution (P_*i-sol*_) that exchanges with both P_*i-lab*_ and P_i-sec_. P_*i-sol*_ is an intermediary pool between P_i-sec_ and P_*i-lab*_ in Wang *et al*.^18^, and thus is key to represent processes of sorption/desorption. P_*i-sol*_ would also allow a better coupling with P uptake model in further studies.
(ii). a Freundlich equation was used to describe the fluxes leaving P_*i-sol*_ to either P_*i-lab*_ or P_i-sec_ (vs. a Langmuir equilibrium between P_ISEC_ and P_ILAB_ in (model = GPASOIL-v0)). Freundlich equations were assumed to be better (see the introduction of Wang *et al*.^18^).
(iii). in (model = GPASOIL-v0), all parameters describing soil P fluxes – except those involve in the Langmuir equilibrium, which vary as function of soil orders- were constant in space (Text S1). In (model = GPASOIL-v1), parameters involved in the soil inorganic P dynamics varied in space following Wang *et al*.^18^.
(iv). the flux from P_x-occ_ to P_i-sec_ was omitted in (model = GPASOIL-v0) but is resolved in (model = GPASOIL-v1)
(v). (model = GPASOIL-v0) was based on yearly time-step, while (model = GPASOIL-v1) was based on daily one, and
(vi). while soil organic P dynamics was similar between the two model versions, one alternative couple of residence time were tested in (model = GPASOIL-v1) (same residence time as in model = GPASOIL-v0 for GPASOIL-v1.0 while another one for GPASOIL-v1.1, Table 11).

### Effect of land-use and land-cover change (LUCC) on cropland/grassland soil P

Within a given grid-cell, the change in the different land-cover fractions from one year to the other modified the soil P of cropland (or grassland), if there were some gross conversions from other land-cover to cropland (grassland respectively). Thus, the effect of LUCC on cropland soil P pools for year *y* and grid-cell *g* (called ${P}_{m}\left(y,crop,g\right)$ with subscript *m* referring to a given soil P pool) was simulated using Eq. 18 as follows:

18 $$\begin{array}{lll}frac(y,crop,g)\ast {P}_{m}(y,crop,g) & = & [frac(y-1,crop,g)\\  &  & -{\sum }_{i\in lu\backslash \{crop\}}{\Delta }_{i}(y-1,crop,g)]\\  &  & \ast {P}_{m}(y-1,crop,g)\\  &  & +{\sum }_{j\in lu\backslash \{crop\}}({\Delta }_{crop}(y-1,j,g)\\  &  & \ast {P}_{m}(y-1,j,g))\end{array}$$

with *frac(y,crop,g)* was the fraction of the grid-cell *g* covered by cropland, Δ_i_(y,j,g) was the change in cover area from land-use *j* to land-use *i*, *lu* is the list of land-use considered (i.e. *lu* = {*crop, grass, nonagri, urban*}), *lu\{n}* means the list of land-use *lu* after removing {*n*}. A similar equation where replacing *crop* by *grass* described the effect of LUCC on grassland soil P pools. The increase/decrease in a given soil pool P_m_ of a given land-cover (cropland or grassland) through LUCC was called $f{P}_{lucc}$ and was positive when P_m_ increases from one year to the other.

The definition of *frac* was the following:

19 $$frac\left(y,lu,g\right)=Area\left(y,lu,g\right)/land\left(g\right)$$

with *Area* for the area of the grid-cell covered by *lu* and *land* the total land area in the grid-cell *g*.

The soil P content of *nonagri* land-cover fraction was not explicitly simulated and was defined as follows:

20 $${P}_{m}\left(y,nonagri,g\right)={P}_{m}^{NA}\left(g\right)$$

where ${P}_{m}^{NA}\left(g\right)$ is the current soil P pool *m* of natural (unmanaged) soils and grid-cell *g*. ${P}_{m}^{NA}\left(g\right)$ was provided by He *et al*.^14^ for *m* within {P_i-lab_, P_i-sec_, P_i-prim_, P_o-lab_, P_o-sta_, P_x-occ_}. ${P}_{i-sol}^{NA}\left(g\right)$ was not provided by He *et al*.^14^ and for this pool, we used the following equation:

21 $${P}_{i-sol}^{NA}\left(g\right)={P}_{c,\infty }.\overline{{W}_{{\rm{abs}}}\left(g\right)}$$

with ${P}_{c,\infty }$ defined in (Eq. 16) and $\overline{{W}_{{\rm{abs}}}}$ the soil water content, as defined earlier.

Through Eq. 20, we assumed that the P inherited from natural soils at the conversion to agriculture (the so-called biogeochemical background, BIOG) could be represented by prescribing the current P in unmanaged soils to all soils converted to agricultural soils over the last ~120 years. As explained in Ringeval *et al*.^11^, this could be limiting, in particular in regions where shifting cultivation occurred, leading to modification of the P content of soils covered by natural vegetation. We also neglected soil P input corresponding to forest biomass left on soil at the time of conversion^28^. All these simplifications had to be taken unless we explicitly simulated the change of soil P in non-agricultural areas, which would require the use of global vegetation models that rely on their own assumptions (e.g. Sun *et al*^.29^).

In addition, P in natural soils was also used as initial conditions, i.e.:

22 $${P}_{m}\left({y}_{0},crop,g\right)={P}_{m}^{NA}\left(g\right)$$

23 $${P}_{m}\left({y}_{0},grass,g\right)={P}_{m}^{NA}\left(g\right)$$

with *y*_*0*_ corresponds to the 1^st^ year of the simulation, chosen here equal to 1900.

The soil P content of *urban* land-cover fraction was not explicitly simulated and was defined by:

24 $${P}_{m}\left(y,urban,g\right)=0$$

Assumption of neglected soil P pools for urban land-cover was likely wrong especially for urban green areas but this assumption had no effect on cropland/grassland soil P as conversion from urban areas to agricultural areas was extremely rare.

### Drivers of agricultural soil P distribution: datasets and computation

As explained earlier, the term “drivers” encompasses variables related to soil P input and output (farming practices - FARM, atmospheric deposition -DEPO, sludges - SLUD, losses through water erosion - LOSS), land-use change (LUCC), variables that have an effect on soil P dynamics (soil properties - SPRO, climate - CLIM), and biogeochemical background (BIOG). The different drivers and their representations were summarized in Tables 1–8. The datasets used in this study are called (data = GPASOIL-v1) while (data = GPASOIL-v0) is used to name the datasets used in Ringeval *et al*.^11^. Tables 1–8 provide the difference between (data = GPASOIL-v0) and (data = GPASOIL-v1) for each driver. BIOG and SPRO were constant in time while other drivers varied in time. Drivers whose the computation required more information than the one given in Tables 1–8 were described below.

#### FARM

##### P in manure (for both cropland and grassland)

Two estimates of P in manure applied to cropland soil and two estimates for grassland soil were used to assess the uncertainty associated with this soil input (Section “Error estimate…” and Supplementary Table S1). For each land-use category (cropland or grassland), the first estimate relied on a half-degree resolution dataset for N in manure reaching the soil (Zhang *et al*.^30^ for cropland, $f{N}_{manu,Zhang}^{out\to x-tot}$, Eq. 25, and Xu *et al*.^31^ for grassland, $f{N}_{manu,Xu}^{out\to x-tot}$, Eq. 26). A spatially constant P:N ratio of 0.20 (kgP kgN^−1^) was used to convert N fluxes to P fluxes of manure.

25 $$f{P}_{manu,1}^{out\to x-tot}(y,crop,g)=0.20\ast f{N}_{manu,Zhang}(y,crop,g)$$

26 $$f{P}_{manu,1}^{out\to x-tot}(y,grass,g)=0.20\ast f{N}_{manu,Xu}(y,grass,g)$$

$f{N}_{manu,Xu}^{out\to x-tot}$ correspond to the sum of N in what was called deposition by grazing animals and application of manure by Xu *et al*.^31^.

The second estimate was computed thanks to Eqs. 27, 28 and relied on the P in manure produced by the livestock at the country-scale given by Demay *et al*.^9^, which combined data on of livestock population and P excretion rates per livestock category, without any distinction between cropland and grassland soils:

27 $$f{P}_{manu,2}^{out\to x-tot}\left(y,crop,g\right)=f{P}_{manu,1}^{out\to x-tot}\left(y,crop,g\right)\ast \frac{\overline{f{P}_{manu,Demay}\left(crop+grass,country\left(g\right)\right)}}{\overline{f{P}_{manu,1}^{out\to x-tot}\left(crop+grass,country\left(g\right)\right)}}$$

28 $$f{P}_{manu,2}^{out\to x-tot}\left(y,grass,g\right)=f{P}_{manu,1}^{out\to x-tot}\left(y,grass,g\right)\ast \frac{\overline{f{P}_{manu,Demay}\left(crop+grass,country\left(g\right)\right)}}{\overline{f{P}_{manu,1}^{out\to x-tot}\left(crop+grass,country\left(g\right)\right)}}$$

with the overbar there corresponds to a temporal average over the 1950–2017 period (i.e. the years in common for Demay *et al*.^9^ and $f{P}_{manu,1}^{out\to x-tot}$), country(g) is the country where the grid-cell *g* can be found, $f{P}_{manu,Demay}^{out\to x-tot}$ is the P manure produced at country scale provided by Demay *et al*.^9^ and $f{P}_{manu,1}^{out\to x-tot}\left(crop+grass,country\left(g\right)\right)$ is the country average of variables computed thanks to Eqs. 25, 26 without any distinction crop vs grass. For a given country, $f{P}_{manu,2}^{out\to x-tot}$ has the same relative spatial distribution among grid-cells as $f{P}_{manu,1}^{out\to x-tot}$ but its spatial variabilities between countries is different. The ratio of 0.20 used in Eqs. 25, 26 was given by Sun *et al*.^29^ and corresponds to a P:N ratio averaged among ruminant manure (mean P:N value of 0.165 between value of 0.15 for cattle and 0.18 for goat and sheep^32^) and monogastric manure (mean P:N value of 0.26 between value of 0.24 for chicken and 0.28 for swine^32^), weighted by the global amount of manure for each livestock super-category (ruminants: 14.4 TgN yr^−1^ and monogastrics: 10.1 TgN yr^−1^) given by FAOSTAT for the year 2000. The use of a spatially and temporal invariant P:N ratio for manure was needed given the lack of gridded data about P manure applied on cropland and grassland soil. It was however a simplistic assumption as N and P were partly decoupled due to N losses in manure through NH_3_ volatilization. While applied to the same data of manure application in Sun *et al*.^29^ as used here, the ratio of Lun *et al*.^32^ characterized manure excretion (i.e. before volatilization). Also, the P:N values used to compute the value of 0.20 was representative to a given country (USA)^32^ while such ratio could change as function of the country and farming practices, following its relationship with animal nutrition^33^. Finally, the use of the same ratio for both cropland and grassland was also an approximation as the proportion of monogastric vs ruminant likely differs between cropland and grassland.

##### P in chemical fertilizer (for both cropland and grassland)

Given the lack of global dataset about P fertilizer applied to grassland, a spatially constant P:N ratio of 0.22 for chemical fertilizer was used to derive P in chemical fertilizer for grassland ($f{P}_{chem}^{out\to i-lab}\left(y,grass,g\right)$) from N in chemical fertilizer applied to grassland ($f{N}_{chem,Xu}\left(y,grass,g\right)$) given by Xu *et al*.^31^:

29 $$f{P}_{chem}^{out\to i-lab}\left(y,grass,g\right)=0.22\ast f{N}_{chem,Xu}\left(y,grass,g\right)$$

The ratio of 0.22 corresponds to the ratio of global P chemical fertilizer for managed grassland in 2002–2010 (0.4 TgP yr^−1^ in Lun *et al*.^32^) and the global N chemical fertilizer for grassland for same years (1.8 TgN yr^−1^ in Xu *et al*.^31^). This ratio was computed and used in Sun *et al*.^29^.

P in chemical fertilizer for cropland ($f{P}_{chem}^{out\to i-lab}\left(y,crop,g\right)$) was derived from Lu and Tian^34^, which assumed that all chemical fertilizer was applied to cropland (i.e. grassland did not receive any chemical fertilizer). Here, we corrected the chemical fertilizer applied to cropland by subtracting the chemical fertilizer applied on grassland given by Eq. 29 from Lu and Tian^34^ estimates, i.e.:

for any grid-cell *g* and year *y*:

30 $$\begin{array}{lll}f{P}_{chem}^{out\to i-lab}(y,crop,g) & = & 1/frac(y,crop,g)\ast (f{P}_{chem,LuTian}(y,crop,g)\\  &  & \ast frac(y,crop,g)-f{P}_{chem}^{out\to i-lab}(y,grass,g)\\  &  & \ast frac(y,grass,g))\end{array}$$

with *frac* is the grid-cell fraction occupied by cropland or grassland, and $f{P}_{chem,LuTian}$ was taken from Lu and Tian^34^ (in kgP ha^−1^ yr^−1^). This correction lead to very small change (at the global scale, $f{P}_{chem}^{out\to i-lab}$ over 2008–2018 is 18.1 Tg.yr^−1^ for cropland vs 0.5 Tg.yr^−1^ for grassland).

##### P uptake and P residues for grassland

P uptake ($f{P}_{upta}^{i-lab\to out}$) and P in plant residues ($f{P}_{resi}^{out\to i-lab}$) in grassland were derived from the actual NPP, the part of NPP which is human-appropriated through grazing/mowing (the so-called grazing intensity, GI), as well as from some spatially constant parameters as follows:

31 $$\begin{array}{ccc}f{P}_{upta}^{i-lab\to out}(y,grass,g) & = & \frac{1}{\gamma }\ast \frac{1}{dry}\ast (\frac{{P}_{{\rm{ \% }},above}}{100}\ast \frac{lifespa{n}_{above}}{L}\ast NP{P}_{above}(y,grass,g)\\  &  & +\frac{{P}_{{\rm{ \% }},below}}{100}\ast \frac{lifespa{n}_{below}}{L}\ast NP{P}_{below}(y,grass,g))\end{array}$$

32 $$f{P}_{resi}^{out\to i-lab}(y,grass,g)=(1-GI(y,grass,g))\ast f{P}_{upta}^{i-lab\to out}(y,grass,g)$$

where *γ* is the carbon content of dry matter (in kgC (kgDM)^−1^), *dry* is the ratio between dry-matter biomass and fresh-matter biomass (kgDM (kgFM)^−1^), P_%_ is the P concentration of grass biomass (in gP (100gFM)^−1^), lifespan is the life duration of plant organs (in year), *L* is the length of the growing season (in years), NPP is the annual NPP (expressed here in kgC ha^−1^ yr^−1^), GI is the so-called grazing intensity, corresponding to the part of NPP which is grazed/mowed (no unit). A distinction aboveground vs belowground was done for *P*_%_*, lifespan*, and *NPP*. Contrary to estimates based on carbon stock (such as used for cropland below), NPP-based estimates should consider a recycling through biomass turnover^35^, that is why we rely here on leaf lifespan (*lifespan*_*above*_, in yr) and root lifespan (*lifespan*_*root*_, in yr) derived from observations divided by the length of the growing season (*L*).

Lifespan for root show large variations among climatic zones and root diameter (fine vs coarse)^36^. Here, we used a lifespan_below_ of 0.7 yr, close to the value provided by Zhang and Wang^37^ for graminoids. The parameter lifespan_above_ was set to 0.8 yr slightly below the leaf longevity used in absence of stress in LPJ-GUESS for C3-C4 grasses (1 yr in Smith *et al*.^38^). We set the length of the growing season (L) equal to 1 yr. *γ* is set to 0.45 kgC (kgDM)^−1^, as commonly used (see e.g Monfreda *et al*.^39^). The parameter *dry* is set to 0.20 kgDM (kgFM)^−1^ which is the value of dry fraction of economic yield given by Monfreda *et al*.^39^ for the crop category « alfalfa ».

Terms involved in Eq. 31 are prone to uncertainty, especially NPP^40^ and P concentrations. Here we used the spatial distribution of the actual NPP_tot_ (defined as NPP_tot_ = NPP_above_ + NPP_below_) given by Kastner *et al*.^41^ as they also provided the spatial distribution of GI used in the current study to estimate the P in residues. Kastner *et al*.^41^ provided different estimates and we used here one of them (the so-defined run number 1_3_2_1_1_1 in the Readme file of Kastner *et al*.^41^). In the chosen run, LPJ-GUESS without nitrogen limitation is used to approach NPP_tot_ and its global average reaches ~460 gC m^−2^ yr^−1^, well above the value found in Wang *et al*.^42^ for temperate/C3 grassland (273 gC m^−2^ yr^−1^) but well below global value derived from fields (979 ± 78 gC m^−2^ yr^−1^) found in Sun *et al*.^40^. Thus, we used for each grid-cell g, two estimates for NPP_tot_:

33 $$NP{P}_{tot,1}\left(y,grass,g\right)=NP{P}_{Kastner}\left(y,g\right)$$

34 $$NP{P}_{tot,2}\left(y,grass,g\right)=NP{P}_{Kastner}\left(y,g\right)\ast \frac{\overline{NP{P}_{Sun}}}{\overline{NP{P}_{Kastner}}}$$

with $\overline{NP{P}_{Sun}}$ and $\overline{NP{P}_{Kastner}}$ equal to 979 and 460 gC m^−2^ yr^−1^, respectively. Globally averaged GI is 0.12. NPP_below_ and NPP_above_ are computed as 0.54*NPP_tot_ and 0.46*NPP_tot_, respectively, as found in Sun *et al*.^40^.

Lun *et al*.^43^ provided a value of 1.5e^−1^ gP (100gFM)^−1^ for the P concentration of the economic yield for the crop category « other crops » encompassing « forage and silage (maize, grasses nes, alfalfa, clover,…) », which is close to the value provided by Smil^44^ for the harvest of the crop category « Forages » (2.0e^−1^ gP (100gFM)^−1^) but much higher than the P concentration found in GOLUM^42^ (and taken from Zechmeister-Boltenstern *et al*.^45^) or found in Spohn *et al*.^46^. Wang *et al*.^42^ provided a molar P:C ratio for foliage of 1/753 for temperate C3 grass and 1/1728 for tropical C4 grass (Table S1 of Wang *et al*.^42^), i.e. a C3-C4 averaged molar P:C ratio of 1/1241, very close to the molar P:C ratio of 1/1260 given by Spohn *et al*.^46^ for senescent leaves of grassland. These values correspond to a P concentration of 2.5e^−2^ gP (100gFM)^−1^, following the below equation:

35 $${P}_{{\rm{ \% }},above}=100\ast \gamma \ast dry\ast \frac{M(P)}{M(C)}\ast {P}_{molar,above}$$

with P_molar,above_ the P:C molar ratio (no unit), P_%_ the P concentration in gP (100gFM)^−1^, M(P) and M(C) the atomic mass of P and C. In our study, we used this value (2.5e^−2^ gP (100gFM)^−1^) as lower boundary and the Lun *et al.*^43^ value (1.5e^−1^ gP (100gFM)^−1^) as upper boundary, thus a mean value of 8.8e^−2^ gP (100gFM)^−1^.

Finally, we assumed the following relationship between P_%,above_ and P_%,below_, based on the order of magnitude of leaf:root ratio of molar P:C ratio used in^45^ for C3 or C4 grassland:

36 $${P}_{{\rm{ \% }},below}=\frac{1}{2}{P}_{{\rm{ \% }},above}$$

##### P uptake and P residues for cropland

P uptake at grid-cell scale for the cropland fraction ($f{P}_{uptake}^{i-lab\to out}\left(y,crop,g\right)$) was computed as the sum of the uptake by different crops weighted by the harvested area of each crop:

37 $$f{P}_{upta}^{i-lab\to out}(y,crop,g)=\frac{1}{{\sum }_{c\in lis{t}_{crop}}Area(y,c,g)}.{\sum }_{c\in lis{t}_{crop}}(Area(y,c,g)\ast f{P}_{upta}^{i-lab\to out}(y,c,g))$$

P in plant residues ($f{P}_{resid}^{out\to x-tot}$) was computed as:

38 $$f{P}_{resi}^{out\to x-tot}(y,crop,g)=\frac{1}{{\sum }_{c\in lis{t}_{crop}}Area(y,c,g)}.{\sum }_{c\in lis{t}_{crop}}(Area(y,c,g)\ast f{P}_{resi}^{out\to x-tot}(y,c,g))$$

with *c* a crop within the list of crop considered in Monfreda *et al*.^39^ (called here list_crop_) and Area(y,c,g) the area covered by the crop *c* (in ha). Among 172 crops listed in list_crop_, 14 were not considered as we did not find any corresponding crop in FAOSTAT. It is particularly the case for some crops of the group « forage » in Monfreda *et al*.^39^ (alfalfa, beets for fodder, clover, rye grass for forage and silage, etc.) but not for all, as we made some corresponding between non-forage and forage crop in FAOSTAT. E.g. we used the “maize” crop in FAOSTAT to get some information needed to be combined with the crop “maize for forage” of Monfreda *et al*.^39^. The corresponding between non-forage and forage crop was disputable but it concerned here only the relative change in yield and area as compared to the year 2000 (Eqs. 43, 46). We also excluded carob as carob area in Spain shows very weird pattern in FAOSTAT (from ~130 000 ha on average over 1961–1989 to 450 ha on average over 1990–2017).

For each crop, P uptake and P residues are computed as follows:

39 $$\begin{array}{lll}f{P}_{uptake}^{i-lab\to out}(y,c,g) & = & \frac{Yield(y,c,g)}{100}.[{P}_{{\rm{ \% }},root}(c).\frac{dr{y}_{harvest}(c)}{dr{y}_{root}(c)}.\frac{RSR(c)}{HI(c)}\\  &  & +{P}_{{\rm{ \% }},abov\backslash harvest}(c).\frac{dr{y}_{harvest}(c)}{dr{y}_{abov\backslash harvest}(c)}.\left(\frac{1}{HI(c)}-1\right)+{P}_{{\rm{ \% }},harvest}(c)]\end{array}$$

40 $$\begin{array}{ccc}f{P}_{resid}^{out\to x-tot}(y,c,g) & = & \frac{Yield(y,c,g)}{100}.[{P}_{{\rm{ \% }},root}(c).\frac{dr{y}_{harvest}(c)}{dr{y}_{root}(c)}.\frac{RSR(c)}{HI(c)}\\  &  & +fra{c}_{resid}(c).{P}_{{\rm{ \% }},abov{\rm{\setminus }}harvest}(c).\frac{dr{y}_{harvest}(c)}{dr{y}_{abov{\rm{\setminus }}harvest}(c)}.\left(\frac{1}{HI(c)}-1\right)]\end{array}$$

with P_%,m_ the P concentration (in gP (100gFM)^−1^) for the organ *m* with *m* being {root, abov\harvest, harvest} where « abov\harvest » defines the aboveground biomass excluding the harvest (also called total residues in Smil^44^), dry_m_ the dry proportion of organ *m* expressed in fresh matter (in gDM gFM^−1^), frac_resid_ the fraction of the aboveground biomass excluding the harvest that remains on the soil (the rest being exported from the field), Yield the economic yield (expressed in kgFM ha^−1^), RSR the root:shoot ratio (no unit), HI the harvest index (no unit). Text S2 shows how we get these equations as well as how we get corresponding equations for crops whose the harvested part being root. Following Bentsen *et al*.^47^, we assumed that at harvest, the different organs have the same dry proportion (i.e. dry_grain_ = dry_abov\harvest_ = dry_root_). For all crops but root crops, we considered P_%,abov\harvest_ equal to the P concentration of aboveground residues given by Lun *et al*.^43^ (and completed for few crop categories by Smil^44^). P_%,harvest_ is provided by Lun *et al*.^43^. RSR is derived from the aboveground fraction (frac_above_) given in Monfreda *et al*.^39^ (RSR = 1/frac_above_ −1) and HI is directly given by Monfreda *et al*.^39^. frac_resid_ is equal to 0 for forage crop (group « forage » in Monfreda *et al*.^39^) and equal to 1/2 for other crops, following Smil^44^. We did not find any database available providing the P concentration of roots for different crop categories. Instead we assumed, following Ye *et al*.^48^ (focusing on rice), that for any crop *c*:

41 $${P}_{{\rm{ \% }},root}\left(c\right)=0.75\ast {P}_{{\rm{ \% }},abov\backslash harvest}\left(c\right)$$

The specificities of root crop computation are given in Text S2.

The spatially explicit yield ($Yield\left(y,c,g\right)$ in kgFM ha^−1^) for the crop category *c* is computed as follows:

42 $$Yield\left(y,c,g\right)=Yiel{d}_{Monfreda}\left(2000,c,g\right).\frac{Yiel{d}_{FAO,bis}\left(y,c,country\left(g\right)\right)}{Yiel{d}_{FAO}\left(2000,c,country\left(g\right)\right)}$$

with Yield_Monfreda_ the spatially explicit (half-degree resolution) distribution of yield given by Monfreda *et al*.^39^ for years 2000 (and expressed as weight of economic yield, i.e. with standardized water content), Yield_FAO_ the country-scale FAO yield and Yield_FAO,bis_ defined as follows:

43 $$Yiel{d}_{FAO,bis}\left(y,c,country\left(g\right)\right)=Yiel{d}_{FAO}\left(y,c,country\left(g\right)\right)\;{\rm{if}}\;{\rm{y}} > ={\rm{1961}}$$

44 $$Yiel{d}_{FAO,bis}(y,c,country(g))=Yiel{d}_{FAO}(1961,country(g),c)\ast \frac{pop(y,country(g))}{pop(1961,country(g))}\;{\rm{if}}\;{\rm{y}} < {\rm{1961}}$$

with *pop* the country-scale population defined by HYDE 3.2^23^. With Eq. 42, we combined the spatially explicit yield distribution of Monfreda *et al*.^39^ (but only one value for year 2000) with the temporal varying FAOSTAT yield (but at country-scale). For each crop, the relative intra-country distribution of yield given by Monfreda *et al*.^39^ for year 2000 was kept constant in time. With Eq. 44, we scaled the country-scale yield to the country-scaled human population before 1961, as it was assumed in Bouwman *et al*.^13^. As country spatial boundaries can change in time, we made some computations to allow consistency between each grid-cell and its belonging to a country whose the boundaries can change in time (Text S3).

The harvested area ($Area\left(y,c,g\right)$ in ha) for the crop category *c* for any grid-cell *g* and any year *y* is computed as follows:

45 $$Area\left(y,c,g\right)=are{a}_{Monfreda}\left(2000,c,g\right).\frac{Are{a}_{FAO,bis}\left(y,c,country\left(g\right)\right)}{Are{a}_{FAO}\left(2000,c,country\left(g\right)\right)}$$

with Area_Monfreda_ the spatially explicit distribution of harvested area given by Monfreda *et al*.^39^ for years 2000, Area_FAO_ the country-scale FAO harvested area and Area_FAO,bis_ defined as follows:

46 $$Are{a}_{FAO,bis}\left(y,c,country\left(g\right)\right)=Are{a}_{FAO}\left(y,c,country\left(g\right)\right)\;{\rm{if}}\;{\rm{y}} > ={\rm{1961}}$$

and

47 $$Are{a}_{FAO,bis}\left(y,c,country\left(g\right)\right)=Are{a}_{FAO}\left(1961,c,country\left(g\right)\right)\;{\rm{if}}\;{\rm{y}} < 1961$$

##### Composition of manure and residues for cropland and grassland

The composition (inorganic labile, organic labile, organic stable) of manure and residues were considered constant in space and among cropland/grassland:

48 $$f{P}_{manu}^{out\to i-lab}(y,lu,g)=fra{c}_{i-lab,manu}\ast f{P}_{manu}^{out\to x-tot}(y,lu,g)$$

49 $$f{P}_{manu}^{out\to o-lab}(y,lu,g)=fra{c}_{o-lab,manu}\ast f{P}_{manu}^{out\to x-tot}(y,lu,g)$$

50 $$f{P}_{manu}^{out\to o-sta}(y,lu,g)=(1-fra{c}_{i-lab,manu}-fra{c}_{o-lab,manu})\ast f{P}_{manu}^{out\to x-tot}(y,lu,g)$$

51 $$f{P}_{resi}^{out\to i-lab}(y,lu,g)=fra{c}_{i-lab,resi}\ast f{P}_{resi}^{out\to x-tot}(y,lu,g)$$

52 $$f{P}_{resi}^{out\to o-lab}(y,lu,g)=fra{c}_{o-lab,resi}\ast f{P}_{resi}^{out\to x-tot}(y,lu,g)$$

53 $$f{P}_{resi}^{out\to o-sta}(y,lu,g)=(1-fra{c}_{i-lab,resi}-fra{c}_{o-lab,resi})\ast f{P}_{resi}^{out\to x-tot}(y,lu,g)$$

with *lu* in {crop,grass}, *y* the year and *g* the grid-cell considered, frac_m,l_ corresponding to the fraction of $f{P}_{l}^{out\to x-tot}$ reaching the pool *m*. Following Ringeval *et al*.^11^, we prescribed: $fra{c}_{i-lab,manu}=0.8$, $fra{c}_{o-lab,manu}=0.1$, $fra{c}_{i-lab,resid}=0.4$ and $fra{c}_{o-lab,resid}=0.4$. We considered an uncertainty associated to the composition of residues. To do so, we use a random value between $fra{c}_{i-lab,resi}-50{\rm{ \% }}$ and $fra{c}_{i-lab,resi}+50{\rm{ \% }}$ as well as as between $fra{c}_{o-lab,resi}-50{\rm{ \% }}$ and $fra{c}_{o-lab,resi}+50{\rm{ \% }}$ (see the section about error estimates).

#### DEPO

Different classes of atmospheric deposition of P are often considered based on the sources of P in the atmosphere: mineral dust, sea salt, primary biogenic aerosol particles (PBAP), natural combustion and anthropogenic combustion^49^. We considered that P deposition input enriches both the inorganic labile pool ($f{P}_{depo}^{out\to i-lab}$) and the primary inorganic pool ($f{P}_{depo}^{out\to i-prim}\left(y,lu,g\right)$), as follows:

54 $$\begin{array}{lll}f{P}_{depo}^{out\to i-lab}(y,lu,g) & = & fra{c}_{i-lab,dust}\ast {D}_{dust}(y,g)+fra{c}_{i-lab,other}\ast ({D}_{seasalt}(y,g)\\  &  & +{D}_{PBAP}(y,g)+{D}_{natcomb}(y,g)+{D}_{anthcomb}(y,g))\end{array}$$

55 $$\begin{array}{lll}f{P}_{depo}^{out\to i-prim}(y,lu,g) & = & (1-fra{c}_{i-lab,dust})\ast {D}_{dust}(y,g)+(1-fra{c}_{i-lab,other})\\  &  & \ast ({D}_{seasalt}(y,g)+{D}_{PBAP}(y,g)\\  &  & +{D}_{natcomb}(y,g)+{D}_{anthcomb}(y,g))\end{array}$$

with D_dust_, D_seasalt_, D_PBAP_, D_natcomb_ and D_anthcomb_ are atmospheric P deposition fluxes (in kgP ha^−1^ yr^−1^) of mineral dust, sea salt, PBAP, natural combustion and anthropogenic combustion, respectively. frac_lab,dust_ and frac_lab,other_ are labile fractions of atmospheric deposition of P (no unit) for respectively dust and all other sources (sea salt, PBAP, natural and anthropogenic combustion). frac_lab,dust_ was chosen to be equal to 0.1 while frac_lab,other_ was chosen equal to 0.5^50^.

For any year y of the simulation and for any grid-cell g, we computed ${D}_{dust}\left(y,g\right)$, ${D}_{seasalt}\left(y,g\right)$, ${D}_{PBAP}\left(y,g\right)$, ${D}_{natcomb}\left(y,g\right)$ and ${D}_{anthcomb}\left(y,g\right)$ based on variables provided by Wang *et al*.^49,51^. Wang *et al*.^49^ provided, for any grid-cell *g*, ${D}_{dust}^{A}\left(\overline{2000-2011},g\right)$, ${D}_{seasalt}^{A}\left(\overline{2000-2011},g\right)$, ${D}_{PBAP}^{A}\left(2000,g\right)$, ${D}_{totcomb}^{A}\left(\overline{1960-2007},g\right)$ and for any World regions *reg*, ${E}_{anthcomb}^{A}\left(1960\le y\le 2007,reg\right)$ and ${E}_{natcomb}^{A}\left(1960\le y\le 2007,reg\right)$ while Wang *et al*.^51^ provided, for any grid-cell *g*, ${D}_{all}^{B}\left(1997\le y\le 2013,g\right)$, with *D*: atmospheric deposition of P, *E*: P emissions to the atmosphere, the upper letter corresponding to the version of the dataset of deposition (A: Wang *et al*.^49^ and B: Wang *et al*.^51^), the line over years means that only the data for an averaged time-period is available while a ≤ y ≤ b means that data are available for each year between *a* and *b*, *totcomb* means (*natcomb* + *anthcomb*) while *all* means: *(dust* + *(seasalt* + *PBAP* + *natcomb* + *(anthcomb)*.

To summarize our computation, the strategy was to consider *D*_*dust*_, *D*_*seaslat*_, *D*_*PBAP*_, *D*_*natcomb*_ as static in time, and temporal average given by Wang *et al*.^49^ were used for these variables. *D*_*natcomb*_ (respectively *D*_*anthcomb*_) were derived from *D*_*totcomb*_ and the ratio between *natcomb and totcomb* (resp. *anthcomb* and *totcomb*) in emissions. ${D}_{anthcomb}\left(y,g\right)$ was considered varying in time and its interannual variability was estimated from the interannual variability of emissions from anthropogenic combustion. For years after 2007, Wang *et al*.^51^ were used and corrected to ensure equality with Wang *et al*.^49^ for the years in common (1997–2007). Equations are given in Text S4.

#### SLUD

P in sludges from sewage treatment that reaches cropland soils P ($f{P}_{slu}^{out\to x-tot}$ in kgP ha^−1^ year^−1^) is estimated by combining human P excretions with the fraction of sewage sludge that is treated and the P removal efficiency of treatments plants following van Puijenbroek *et al*.^52^ and Demay *et al*.^9^:

56 $$\begin{array}{lll}f{P}_{slud}^{out\to x-tot}(y,crop,g) & = & \sum _{type=1,2,3}(fra{c}_{treat}(y,country(g),type)\ast NR(type))\\  &  & \ast huma{n}_{excr}(y,reg(country(g)))\ast pop(y,country(g))\\  &  & /Area(y,crop,country(g))\end{array}$$

with human_excr_ the total P in human excretion (in kgP capita^−1^ year^−1^) for big World regions, frac_treat_ is country-scale fraction of total population (no unit) with access to different types of wastewater treatment installations (types 1, 2 or 3), each one being characterized by a different removal fraction of P (NR, no unit with NR = 0.10, 0.45, 0.90 for respectively wastewater treatment installations type 1, 2, 3), *pop* the country-scale human population (in inhabitants), *Area* the country-scale cropland area (in ha).

*NR*, *human*_*excr*_, *frac*_*treatment*_ are provided by van Puijenbroek *et al*.^52^, *pop* is provided by HYDE 3.2^23^ and *Area* at country scale is computed from the dataset used in LUCC. The variable *human*_*excr*_ is available for the years 1970 and 2010. The variable *frac*_*treat*_ is available for years 1990, 2000, 2010 in van Puijenbroek *et al*.^52^ and we computed it for the year 1970 (see Text S5 for further technical details). Then, linear regressions are used to derive *human*_*excr*_ and *frac*_*treat*_ for any year between 1970 and 2010. Values for year 1970 (resp. 2010) were used for any years before (resp. after) 1970 (resp. 2010). The corresponding between big World regions and countries was provided by the IMAGE framework region classification (https://models.pbl.nl/image/index.php/Region_classification_map).

The Eq. 56 relies on the following assumptions:

- P in sludges is solely sourced from human food consumption (thereby excluding P release from detergents)

- all sludges produced are used in agriculture. This is a strong assumption as a few countries have specific rules banning the use of sludge in agriculture (e.g. Switzerland incinerated sludges^53^,) but this assumption was required as we did not find any database that compiled this information per country at the global scale

- all sludges used in agriculture are spread on croplands (not on grasslands).

For the composition of P in sludges, we used the same labile vs. stable contribution as for manure without considering any uncertainty related to the composition (Supplementary Table S1).

#### LOSS

The annual lateral flux of P lost from soil erosion by water (in kgP ha^−1^ yr^−1^) was computed by combining the fraction of soil mass of the top 0–0.3 m layer lost each year through erosion (frac_loss_, in yr^−1^) with each soil P pools (kgP ha^−1^):

57 $$f{P}_{loss}^{m\to out}\left(y,lu,g\right)={P}_{m}\left(y,lu,g\right)\ast fra{c}_{loss}\left(y,lu,g\right)$$

with m in {i-lab, o-lab, o-sta, i-prim, i-sec, i-occ}, lu in {crop, grass}, and P_m_ the soil P content of pool *m* simulated by our approach.

frac_loss_ was computed by combining the gross soil losses by water erosion with a cropland/grassland distinction (*loss*, in (kg of soil) ha^−1^ yr^−1^), the soil bulk density of the fine earth fraction (bdod) and the volumetric fraction of coarse fragment (cfvo) to estimate the soil mass of the top 0–0.3 m layer:

58 $$fra{c}_{loss}(y,lu,g)=\frac{loss(2000,lu,g)}{bdod(g)\ast (1-cfvo(g))\ast \Delta z\ast {10}^{+4}}$$

with *bdod* the bulk density for the top 0–0.3 m soil layer (in (kg of fine soil) m^−3^), *cfvo* is the volumetric fraction of coarse fragment (without unit), Δz = 0.3 m and 10^+4^ a converting factor in m² ha^−1^. The variable *loss* was computed following the approach described in Borrelli *et al*.^54^ but here applied to the land-use dataset described for year 2000 in LUCC. The geo-statistical approach proposed by^ Borrelli *et al*.54^ allowed for an accurate spatial definition of the land uses (native resolution at 250 m cell size at the equator aggregated to 0.5° latitude × 0.5° longitude) and the consideration of the effects of the different regional cropping systems.

As in Ringeval *et al*.^11^, Eq. 58 is based on the following assumptions. First, we assumed that the soil losses of Borrelli *et al*.^54^ corresponded to a loss of topsoil (i.e. 0–0.3 m soil layer). Second, the estimates of losses for year 2000 (in (kg of soil) ha^−1^ yr^−1^) were considered representative of erosion rates for the whole century. Last, the estimates from Borrelli *et al*.^54^ correspond to a gross erosion rate and not to the net flux, which results from both mobilization and processes, such as deposition, storage and burial. However, we considered that these later processes were not relevant to the system represented here, either because they largely occur in non-agricultural fractions of the grid-cell (e.g. deposition on river banks) or because they concerned soil horizons below 0.3 m (burial).

#### LUCC

The following land-use categories are considered in our approach: cropland (*crop*), grassland (*grass*), non-agricultural vegetation (*nonagri*), and urban (*urban*). For each year *y* and grid-cell *g*, we needed the fractions of cropland ($frac\left(y,crop,g\right)$) and of grassland ($frac\left(y,grass,g\right)$) as well as the transitions between the above mentioned 4 land-use categories (i.e. ${\Delta }_{i}\left(y,j,g\right)$) for the conversion from *j* to *i* with both *i* and *j* in {*crop, grass, nonagri, urban*}). These variables were estimated based on Chini *et al*.^20^ after we made the corresponding between our land-use categories (left hand side) and the ones considered in Chini *et al*.^20^ (right hand side):

59 $$cropland=c3ann+c3nfx+c3per+c4ann+c4per$$

60 $$grassland=pastr+range$$

61 $$nonagri=primf+primn+secdf+secdn$$

62 $$urban=urban$$

with the following meaning for categories in Chini *et al*.^20^: *c3ann*: C3 annual crops, *c3nfx*: C3 nitrogen-fixing crops, *c3per*: C3 perennial crops, *c4ann*: C4 annual crops, *c4per*: C4 perennial crops, *past*: managed pasture, *range*: rangeland, *primf*: forested primary land, *primn*: non-forested primary land, *secdf*: potentially forested secondary land, *secdn*: potentially non-forested secondary land, *urban*: urban land.

#### CLIM

We used the simulations performed by land-surface models for the CMIP6 exercise^55^ to get information about the soil liquid water content (*W*_*rel*_, in fraction of the field capacity and W_abs_, in L(kg of soil)^−1^) and the soil temperature (*T*_*soil*_, in °C) for the top soil layer considered in our approach (0–0.3 m) over the historical period. All CMIP-6 simulations providing the following variables at the basis of our computation of *W*_*rel*_*, W*_*abs*_ and *T*_*soil*_ have been used: *mrsll* (liquid water content of soil layers, in kg.m-2), *mrsofc* or *fldcapacity* (field capacity, in kg m^−2^), *tsl* (soil temperature, in °C). The choice of *mrsofc* or *fldcapacity* to approach the field capacity of a given land-surface model depends if the field capacity varies with depth in this land-surface model. Finally, the average among 9 simulations (combination between 4 land-surface models: CNRM-ESM2-1, CNRM-CM6-1, IPSL-CM6A-LR, MIROC6; and 3 climate data used as input of the land-surface models for the historical period: land-hist, land-hist-cruNcep, land-hist-princeton; see Table 6 for the reference of each simulation) has been computed and used. The period 1850–2012 was common to the 9 simulations. For years after 2012, to prevent any bias due to the lack of some simulations, we used the 1993–2012 climatology each year.

The annual average of each variable (*W*_*abs*_*, W*_*rel*_ and *T*_*soil*_) was used in our approach. The influences of *T*_*soil*_ and *W*_*rel*_ on weathering and mineralization were modelled using different functions (*h*_1_ and *g*_1_ for sensitivity to *T*_*soil*_, *h*_2_ and *g*_2_ for sensitivity to *W*_*rel*_) in both (model = GPASOIL-v0) and (model = GPASOIL-v1). *W*_*abs*_ was used in (model = GPASOIL-v1) in some parametrizations (Eqs. 12, 13) and to translate P_i-sol_ into soil solution P concentration (Eqs. 3, 7). In addition, the near-surface atmospheric temperature needed in one parametrization of (model = GPASOIL-v1) (T_a_ in Table 10) was taken from climate data sets used as input of the land-surface models quoted above.

### Error estimate from uncertainty associated with the datasets to describe the different drivers

While we recognized the existence of different sources of uncertainty (dataset, model structure, parametrization^56^), we mainly focus here on how the uncertainty related to the representation of the different drivers propagated to the simulation of current cropland/grassland soil P pools. Nevertheless, the uncertainty associated to SPRO has an effect on the model parameterizations. The uncertainty associated to mineralization rates (model parameter) was investigated separately.

To investigate the propagation of the uncertainty associated with the driver datasets, we performed 100 simulations by using a random value for each variable related to a given driver (see the list of variables in the 3^nd^ line of Tables 1–8) with all grid-cells considered as independent. For each driver, how the random value was defined was described in Supplementary Table S1 and summarized here. The uncertainty associated with the driver LUCC was not considered given the difficulty to properly quantify it by keeping consistency between the different variables involved in LUCC (fraction and transitions between the different land-use). The coefficient of variation computed over the 100 simulations was used hereafter as uncertainty estimate.

For a given driver, when two estimates are considered (called hereafter E1 and E2), we choose to use a normal distribution with 4σ between E1 and E2, which means that ~96% of the random values chosen will fall between the two estimates. This means that we considered E1 and E2 as lower/upper boundaries and we considered the range they defines as encompassing most values possible. Instead of a normal distribution, a uniform distribution was considered for variable that we considered very uncertain (P in manure for cropland and grassland, residues composition) as we considered each estimate (E1 or E2) as reasonable as the other estimate. For a few variables related to some drivers for which only one estimate was available (called hereafter E) and no uncertainty was provided (i.e. P in chemical fertilizer for grassland, P uptake for cropland, residues composition, P in sludge), we derived E1 and E2 as (1 − x)*E and (1 + x)*E with x either equal to 30% or 50% depending on how we consider the driver uncertain, i.e. a standard-deviation equal to 15% or 25% of the mean, respectively.

Random values can lead to unmeaning values for few grid-cells. To prevent this, we set the minimum value allowed for soil input/output to zero. For variables related to other drivers (BIOG, CLIM, SPRO), we prescribed the minimum/maximum values by using the global spatial minimum/maximum of the mean value (Supplementary Table S1). For temporal varying drivers, we consider the uncertainty related to the different time-steps as independent.

In the following, we focused on the simulation performed with the mean value for each driver instead of the mean among the 100 simulations performed to assess the uncertainty. Both are close except for grid-cells with very small soil P pools. In these grid-cells, if the combination of random soil input/output lead to negative values of P_i-lab_, P_i-lab_ is set to 0 and thus, the mean of the 100 simulations was artificially increased, leading to values larger than the simulation performed with mean drivers. As consequence, the coefficient of variation (instead of the standard deviation) among the 100 simulations was used to assess the uncertainty in soil P pools.

One additional set of 100 simulations was performed to estimate the role played by the uncertainty in BIOG on the uncertainty of P_i-lab_. In this set, we used the mean value for all variables related to BIOG and considered uncertainty for all other drivers. Difference between the CV computed with this set of 100 simulations and the one computed in the set where all drivers are uncertain allows to distinguish the role played by BIOG.

## Data Records

One tgz file called GPASOIL-v1_output.tgz was made available on recherche.data.gouv.fr^57^ and contains the soil P pools simulated with (data = v1) and (model = v1.1), as described earlier, in a netcdf format. These soil P pools correspond to GPASOIL-v1.1 (cf. Table 11). To assess the uncertainty related to mineralization rates, we also provided the GPASOIL-v1.0 soil P pools, i.e. the pools simulated with (data = v1) and (model = v1.0). Concerning the coordinate systems, all variables are referenced to the World Geodetic System (WGS84). The cell size is 0.5 decimal degrees. Fluxes of P corresponding to soil P input/output and to soil P dynamics are also given in the same file. Output are provided for the period 1900–2018 at annual time-step for both cropland and grassland. Fluxes were expressed in kgP ha^−1^ yr^−1^ and pools were expressed in kgP ha^−1^, and are both representative to the top soil layer 0–0.3 m. Two files are provided: one corresponding to the simulation with mean estimate for each driver and another one corresponding to the coefficient of variation to assess the effect of the uncertainty in driver estimate on the simulated soil P pools. README_output_upload.txt describes the netcdf files. An additional netcdf file with the land area was also provided to allow users to compute global averages of soil P pools.

## Technical Validation

Our technical validation evaluated first each component of our approach, namely i) the driver estimates based on published datasets and ii) the model of soil P dynamic. Then it focused on how the update of each component (dataset, model) modified the soil P maps simulated from GPASOIL-v0 to GPASOIL-v1. This is part of the technical validation as it allows us to trace the impact of the update of each component on the reconstructed soil P. In this section, different combinations (data x model) were used (Table 11). Then, we discussed about how our best estimates (GPASOIL-v1.1) compared to previous published modelling products Zhang *et al*.^12^ (McDowell *et al*.^58^ and GPASOIL-v0). Finally, we compared the spatial variability simulated by GPASOIL-v1.1 to the one of published regional datasets on measured soil P.

### Evaluation of the computation of some selected drivers

The estimate of the drivers considered in our approach (BIOG, FARM, etc. see Tables 1–8) was based on published datasets. While the estimate was straightforward from published datasets for some drivers (e.g. BIOG), some additional computations were required for others, in particular for P uptake (with strong differences in the computation between cropland and grassland), P in manure and P deposition. In the current section, we checked that our own computation performed well, i.e. that we succeed in applying the equations given in the section named “Drivers of agricultural soil P distribution: datasets and computation”. If possible, we compared some intermediary variables (e.g. yield per crop category, which is an intermediary variable of the computation of P uptake for cropland) or final P-related variables (e.g. P in manure) to other independent datasets.

**Fig. 2:**
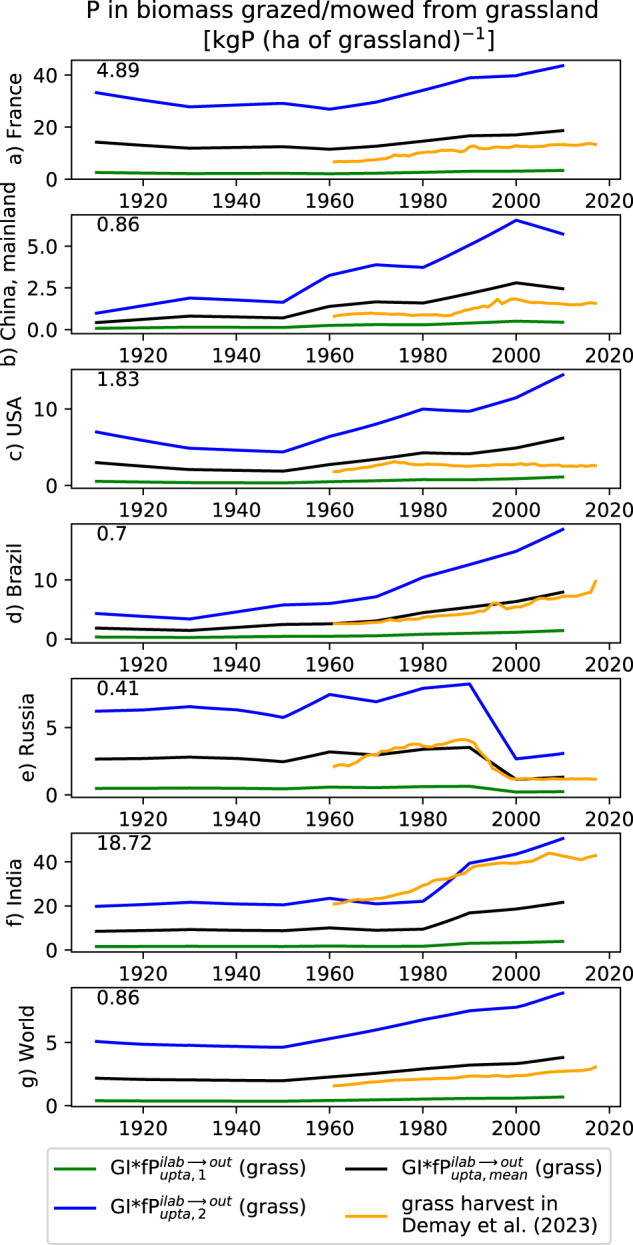
Comparison at country-scale between P in biomass grazed/mowed from grassland estimated in this study and estimates provided by Demay *et al*.^9^. P in biomass grazed/mowed from grassland is defined as GI*fPuptake in our study. Two estimates used in our study are plotted (subscript 1 in green, subscript 2 in blue) as well as the mean of these two estimates (subscript ‘mean’ in black). P in biomass grazed/mowed from grassland in Demay *et al*.^9^ (called P harvest from grassland in this reference) is plotted in orange and is an independent estimates. All fluxes are expressed in kgP (ha of grassland)^−1^. Estimate 1 (respectively estimate 2) corresponds to the min (resp the max) value for all grid-cells within the country considered. “World” (panel (g)) corresponds to the sum of all countries considered in both our estimates and Demay *et al*.^9^ and thus excludes few countries not available in Demay *et al*.^9^. The number in the left-top corner of each panel corresponds to the root mean square error (kgP (ha of grassland)^−1^) between orange and black curves for years in common.

In Fig. 2, we compared our estimates of P in biomass grazed/mowed from grassland to the estimate computed by Demay *et al*.^9^. P in biomass grazed/mowed from grassland was defined as the difference $f{P}_{upta}^{i-lab\to out}(grass)-$ $f{P}_{resi}^{out\to x-tot}(grass)$, or following Eq. 32 as $GI\ast f{P}_{upta}^{i-lab\to out}\left(grass\right)$. The two estimates used to assess the uncertainty ($GI\ast f{P}_{upta,1}^{i-lab\to out}\left(grass\right)$, $GI\ast f{P}_{upta,2}^{i-lab\to out}\left(grass\right)$) and their mean ($GI\ast f{P}_{upta,mean}^{i-lab\to out}\left(grass\right)$) were plotted in Fig. 2. P in biomass grazed/mowed from grassland in Demay *et al*.^9^ (called P harvest from grassland in this latter reference) was computed at country scale based on forage P demand of livestock (Eq. S4 of Demay *et al*.^9^). We found that the independent estimate of Demay et al.^9^ was very close to the mean of our estimates (black curve in Fig. 2).

P uptake for cropland was based on the spatially explicit temporal yield per cropland that we estimated using Eq. 42. Figure S1 shows how the Eq. 42 provided temporal estimates of yield for maize at grid-cell scale (*Yield* in kgFM ha^−1^, line 2 of Fig. S1) by combining Yield_Monfreda_, the spatially explicit yield given by Monfreda *et al*.^39^ for circa the year 2000 (line 4) with Yield_FAO_, the country-scale annual FAO yield (line 3). Within each country, the relative spatial variability was kept constant for any year and equal to one of Yield_Monfreda_. Figure S1 focuses on the temporal averages over three time-periods (1982–1992, 1993–2003, 2004–2014) as it allows to compare our estimates with estimates of Iizumi and Sakai^59^ (line 1) available over the 1982–2014 time-period. Overall, a relatively good agreement is found despite some discrepancies between our estimate and^59^, explained by some mismatch in country-scale variability (e.g. USA) or global distribution of the crop area (e.g. India) between Monfreda *et al*.^39^ and Iizumi and Sakai^59^. Note that Iizumi and Sakai^59^ assumed no temporal change in harvested area and focused only on few crops, that is why we only used this dataset for comparison in the current study.

Figure S2 focuses on the temporal variation at country scale of *Yield* and *Area*, the spatially explicit and crop-specific yield and harvested area, respectively. *Yield* and *Area* are computed thanks to Eqs. 42, 45. As Fig. S1, Figure S2 focuses on maize (c = maize in Eqs. 42, 45). We compared *Yield* to the dataset of Monfreda *et al*.^59^ and *Area* to the dataset used to characterize LUCC^20^ for the broad crop category encompassing maize in LUCC (namely “c4ann” in Chini *et al*.^20^). LUCC is used in our study but not within the computation of the cropland P uptake and thus is independent to its computation. Figure S2 shows that our procedure allows both *Area* and *Yield* to match Monfreda et al.^39^ at country-scale for the year 2000 and to mimic the year-to-year variability of FAOSTAT (left columns of Fig. S2). When we put together all crops belonging to the category « c4ann » of LUCC, we found that the simulated year-to-year variability of area differ to the one given by LUCC (except for Brazil) but that the order of magnitude of area per country relatively matches the one of LUCC. Crop composition of total P uptake is given in Fig. S3.

**Fig. 3:**
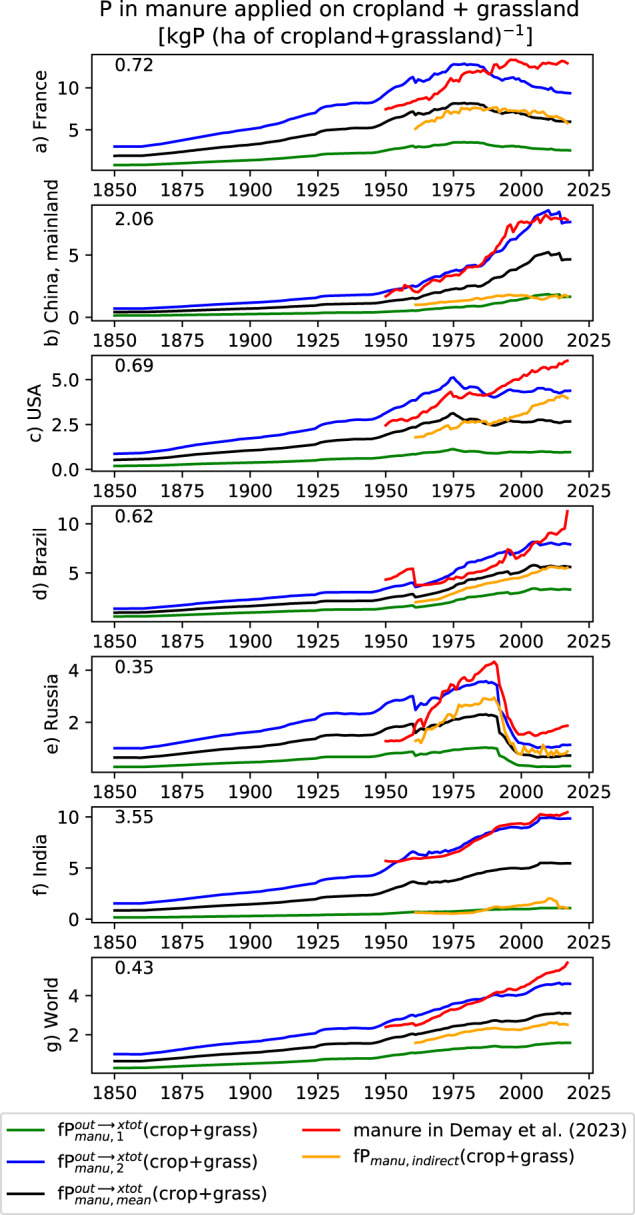
Comparison at country-scale between the two estimates of P in manure applied on cropland + grassland used in our approach ($f{P}_{manu,1}^{out\to x-tot}$ in and $f{P}_{manu,2}^{out\to x-tot}$). Temporal country-scale average of Demay *et al*.^9^ (red curve) is used to scale the country-scale average of $f{P}_{manu,2}^{out\to x-tot}$ while the year-to-year variability of $f{P}_{manu,2}^{out\to x-tot}$ follows the one of $f{P}_{manu,1}^{out\to x-tot}$. The mean of our two estimates (subscript ‘mean’) is plotted in black. Independent estimate based on a livestock budget ($f{P}_{manu,indirect}$) is also plotted in orange. All fluxes are expressed in kgP (ha of cropland + grassland)^−1^. Estimate 1 (respectively estimate 2) corresponds to the min (respectively the max) value for all grid-cells within the country considered. “World” in this figure corresponds to the sum of all countries considered in both our estimates and Demay *et al*.^9^ and thus excludes few countries not available in Demay *et al*.^9^. The number in the left-top corner of each panel corresponds to the root mean square error (in kgP (ha of cropland + grassland)^−1^) between orange and black curves for years in common.

In our approach, P in manure applied to cropland and grassland soils was estimated following two different methods. While the 1^st^ estimate was based on half-degree resolution dataset for N in manure and N:P ratios, the 2^nd^ estimate was based on country-scale estimates of Demay *et al*.^9^ with the same inner-country spatial distribution and temporal variability as the 1^st^ estimate. Figure 3 shows the estimate 1 and estimate 2 for few countries and at the global scale. Demay *et al*.^9^ did not distinguished cropland from grassland and we plotted in Fig. 3 the sum of P in manure for the two land-covers. By construction, the country-averages of estimate 2 and Demay *et al*.^9^ over 1950–2017 are equal, and the temporal variability of estimate 2 is similar to the one of estimate 1. Figure 3 shows that estimates 1 and 2 greatly differed, with, e.g. 1.4 kgP/ha for estimate 1 vs 3.9 for estimate 2, over 1961–2017 at global scale (Fig. 3g). On Fig. 3, we also compared our estimates to the following independent country-scale computation (fP_manu,indirect_, in kgP (ha of cropland + grassland)^−1^) based on a livestock budget:

63 $$\begin{array}{lll}f{P}_{manu,indirect}(y,crop+grass,country) & = & 1/Area(y,crop+grass,country)\\  &  & \ast [GI(y,grass,country)\\  &  & \ast f{P}_{upta,mean}^{i-lab\to out}(y,grass,country)\\  &  & \ast Area(y,grass,country)\\  &  & +grai{n}_{feed}(y,country)\\  &  & +mine{r}_{feed}(y,country)\\  &  & -produc{t}_{animal}(y,country)]\end{array}$$

with $GI(y,grass,country)\ast f{P}_{upta,mean}^{i-lab\to out}(y,grass,country)\ast Area(y,grass,country)$ the P in biomass withdraw from grassland (either mowed or grazed) to feed the livestock (in kgP), gain_feed_ and miner_feed_ the grain and mineral used to feed livestock, respectively, and product_animal_ the P in animal products (P in milk, egg and slaughtered animals). The variables gain_feed_, miner_feed_ and product_animal_ are expressed in kgP. The country-scale data required to compute grain_feed_, minera_feed_ and product_animal_ were provided by FAOSTAT and Demay *et al*.^9^. Parameters needed to compute product_animal_ (average carcass weight, carcass yield over live weight, P content of animal live weight, P concentration of egg and milk) were found in Senthilkumar *et al*.^60^. We found that $f{P}_{manu,indirect}$ was close to our mean estimate in both temporal average value (2.6 kgP ha^−1^ for our mean estimate over 1961–2018 at global scale vs 2.2 for $f{P}_{manu,indirect}$) and temporal correlation (e.g. pearson correlation of 1.0, p < 0.05). Interestingly, the year-to-year variability of our estimates and the one of Demay *et al*.^9^ show some consistent patterns for China, India and Russia but less for other countries.

Figure S4a shows that our procedure to compute P deposition allowed to get a consistent global P deposition from anthropogenic combustion over the whole time-period.

### Application of the Wang ***et al***. soil P inorganic dynamics parametrizations at the global scale

**Fig. 4:**
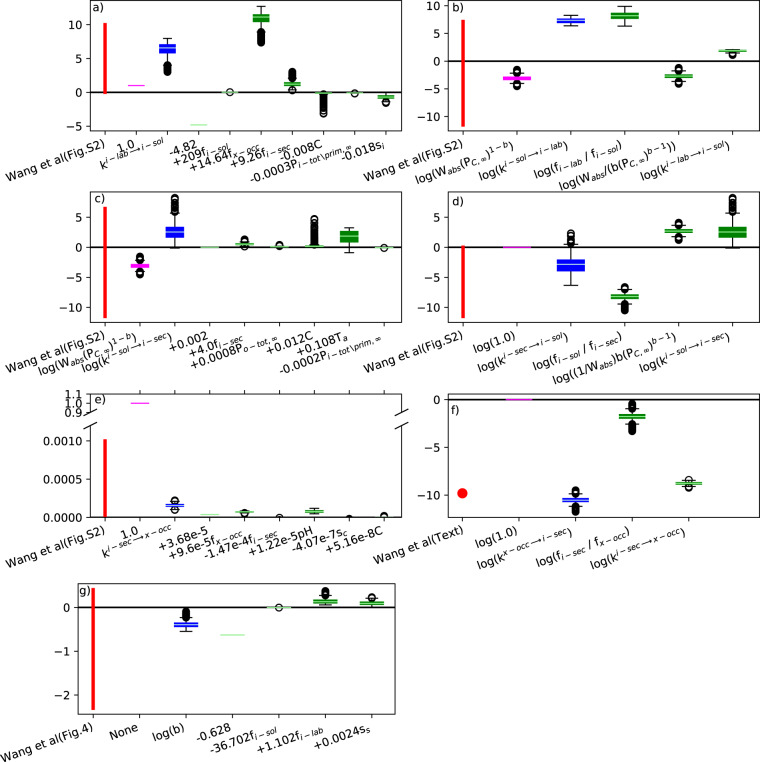
Half-degree grid-cell distribution of each flux parameter involved in (model = GPASOIL-v1). Each panel corresponds to one parameter. In each panel, the red bar gives the min - max range of values for this parameter provided by Wang *et al*.^18^ (mainly from Fig. S2 of Wang *et al*.^18^), the magenta bar corresponds to the values required to make the output flux from a pool smaller than the size of this pool, the blue bar corresponds to the grid-cell distribution of this parameter in the current study and green bars correspond to the distribution of variables used to compute this parameter in the current study. The values required to make the output flux from a pool smaller than the size of this pool are given in magenta. For instance, the gross flux from P_i-sec_ to P_i-sol_ (called $f{P}_{desorp}^{i-sec\to i-sol}$) is computed with Eq. 4: $f{P}_{desorp}^{i-sec\to i-sol}={k}^{i-sec\to i-sol}.{P}_{i-sec}$. The inequality $f{P}_{desorp}^{i-sec\to i-sol} < {P}_{i-sec}$ at daily time-step is equivalent to ${k}^{i-sec\to i-sol} < 1$ (with ${k}^{i-sec\to i-sol}$ in day^−1^) and thus we compared the distribution of ${k}^{i-sec\to i-sol}$ to 1 in the panel (d). Similarly, the gross flux from P_i-sol_ to P_i-sec_ (called $f{P}_{sorp}^{i-sol\to i-sec}$) is computed as follows: $f{P}_{sorp}^{i-sol\to i-sec}={k}^{i-sol\to i-sec}.{\left({P}_{i-sol}/{W}_{{\rm{abs}}}\right)}^{b}$. Thus, the inequality $f{P}_{sorp}^{i-sol\to i-sec} < {P}_{i-sol}$ at daily time-step is equivalent to ${k}^{i-sol\to i-sec} < \overline{{W}_{{\rm{abs}}}}{\left({P}_{C,\infty }\right)}^{1-b}$ with ${k}^{i-sol\to i-sec}$ expressed in mgP (kg soil)^−1^ day^−1^ (mg P/L)^−b^. Thus, we compared the distribution of ${k}^{i-sol\to i-sec}$ to $\overline{{W}_{{\rm{abs}}}}{\left({P}_{C,\infty }\right)}^{1-b}$ in the panel (c) Green bars correspond to the distribution of variables used to compute each parameter. For instance, panel (a) focuses on ${k}^{i-lab\to i-sol}$. The equation to compute this parameter is: ${k}^{i-lab\to i-sol}=-4.82+209{f}_{i-sol}+14.64{f}_{x-occ}+9.26{f}_{i-sec}-$ $0.008C-0.0003{P}_{i-tot{\rm{\backslash prim}},\infty }-0.018{s}_{{\rm{i}}}$, (cf. Table 10), thus the blue bar corresponds to the distribution of ${k}^{i-lab\to i-sol}$ and green bars corresponds to the distribution of −4.82 (1^st^ green bar), $+209{f}_{i-sol}$ (2^nd^ green bar), $+14.64{f}_{x-occ}$ (3^rd^ green bar), etc. This shows the contribution of each variable to the value of the parameter. The parameter b is without unit, ${k}^{i-sec\to x-occ}$, ${k}^{x-occ\to i-sec}$, ${k}^{i-lab\to i-sol}$ and ${k}^{i-sec\to i-sol}$ are in day^−1^, ${k}^{i-sol\to i-sec}$ and ${k}^{i-sol\to i-lab}$ are in mgP (kg soil)^−1^ day^−1^ (mg P/L)^−b^. ${k}^{x-occ\to i-sec}$, ${k}^{i-sol\to i-sec}$ and ${k}^{i-sec\to i-sol}$ are log transform to express them as a sum of another variables.

(model = GPASOIL-v1) was based on Wang *et al*.^18^ parametrization for soil inorganic P dynamics. Parameters used in our approach follow the relationships with pedo-climatic variables found in Wang *et al*.^18^ with some modifications (all k parameters constant in time, oxalate excluded from parametrization leading to both simplified equation for k parameters and ${P}_{c,\infty }$ constant in space). Soil P fractions used in the relationships to compute the different parameters were given by the initial conditions chosen for the soil P pools (i.e. ${P}_{c,\infty }$ = 0.1 mgP L^−1^ for P_i-sol_ and P of unmanaged soil P given by He *et al*.^14^ for other pools). Figure 4 shows that the spatial distribution of parameters involved in (model = GPASOIL-v1) fall well within the range provided by Wang *et al*.^18^ (blue boxplot vs. the range in red in Fig. 4). Nevertheless, we found that the numerical values of some parameters would sometimes require shorter time-step than daily one used here (blue boxplot vs. magenta box in Fig. 4). Indeed, magenta box in Fig. 4 shows the upper value behind which the net flux is larger than the pool it leaves at daily time-step. The issue was particularly true for ${k}^{i-sol\to i-sec}$ whose the global median in (model = GPASOIL-v1) (12.6 mgP (kg soil)^−1^ day^−1^ (mg P/L)^−b^) is well above the value consistent with a daily time-step ($\overline{{W}_{{\rm{abs}}}}{\left({P}_{C,\infty }\right)}^{1-b}$ has a median value of 0.05 mgP (kg soil)^−1^ day^−1^ (mg P/L)^−b^) (Fig. 4). As an equilibrium was assumed between P_i-sol_ and P_i-sec_, this issue does not exist for neither ${k}^{i-sol\to i-lab}$ nor ${k}^{i-lab\to i-sol}$. To solve this issue, a shorter time-step should be chosen, but the time-step needed (e.g. ~5 minutes based on median values given above) is not consistent with global application. Our strategy was to not put any constraints on the parameters themselves nor on each gross flux but we prevent a net flux to be larger than the pool from which this net flux leaves.

Figure S5 shows the sensitivity to the time-steps chosen (1-day or 6-hours time-steps) on a given grid-cell. In such sensitivity tests, the flux parameters were modified by multiplying them by the ratios of the different time-steps. The effect is particularly visible on time-steps with positive soil P budget (Figs. S5, S6) for which the inconsistency between a long time-step (1 day) and ${k}^{i-sol\to i-sec}$ reduces the net flux from P_i-sol_ to P_i-sec_, letting the soil P input within P_i-lab_. The effect of the time-step chosen could be significant at grid-cell scale (e.g. + 17% larger P_i-lab_ for 1-day time-step than for 6-hours time-step in 2010, panels (b) and (c) of Fig. S5) or regionally (−15% lower P_i-lab_ in China for 1-day time-step than for 2-days time-step, Fig. S6a) but remains small at the global scale (few percent of difference in the global average of both P_i-sec_ and P_i-lab_ between 1-day time-step vs 2-days time-step, Fig. S6).

### Effect of the update of each component (data and model) from GPASOIL-v0 on P_i-lab_ simulated in 2018

#### Effect of the update of the dataset to describe the drivers (from (data = GPASOIL-v0; model = GPASOIL-v0) to (data = GPASOIL-v1; model = GPASOIL-v0))

Here we focus on the effect of using new datasets to describe the different drivers with using the same model as the one of Ringeval *et al*.^11^ (i.e. model = GPASOIL-v0). Figure S7 shows first that (data = GPASOIL-v1) modified the simulations of P_i-lab_ and P_x-tot_ for both cropland and grassland (comparison between 1^st^ and last line of Fig. S7). In particular, P_i-lab_ of grassland is strongly reduced with (data = GPASOIL-v1) (20 kgP ha^−1^ at the global scale in 2005) as compared to (data = GPASOIL-v0) (208 kgP ha^−1^). Sensitivity tests (Fig. S7) show that BIOG explains the most the difference between (data = GPASOIL-v1) and (data = GPASOIL-v0) for P_x-tot_. Conversely, FARM is the main driver of the difference between (data = GPASOIL-v1) and (data = GPASOIL-v0) for P_i-lab_ of grassland, and a combination of BIOG and FARM explains the difference for P_i-lab_ of cropland. The key roles played by BIOG and FARM in the spatial distribution of simulated soil P pools were already demonstrated in Ringeval *et al*.^11^.

The biggest difference in FARM between (data = GPASOIL-v0) and (data = GPASOIL-v1) for grassland concerns the P uptake which is much larger in (data = GPASOIL-v1) than in (data = GPASOIL-v0) (Fig. S8). In (data = GPASOIL-v0), the plant uptake of grassland was constrained to be equal to ~90% of the total P input^13^.

The spatial extent of simulated P_i-lab_ is larger in (data = GPASOIL-v1) (~36000 grid-cells and 1481 millions of ha for cropland; ~41000 grid-cells and 3237 millions of ha for grassland) than in (data = GPASOIL-v0) (~15000 grid-cells and 1048 millions of ha for cropland; ~20000 grid-cells and 2215 millions of ha for grassland). This is explained by a Boolean treatment of cropland in Bouwman *et al*.^13^ (with grid-cells with small cropland fraction not considered) and resulting to exclusion of around 1/3 of the real cropland area in GPASOIL-v0.

#### Effect of the update of soil P dynamic model (from (data = GPASOIL-v1; model = GPASOIL-v0) to (data = GPASOIL-v1; model = GPASOIL-v1))

**Fig. 5:**
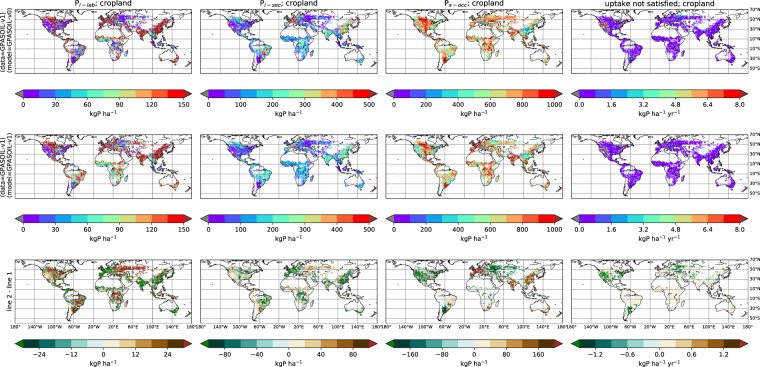
Effect of using (model = GPASOIL-v1) instead of (model = GPASOIL-v0) on simulated soil P pools for cropland. First line shows the simulation with (model = GPASOIL-v0) while the second line shows (model = GPASOIL-v1). All simulations have been performed with (data = GPASOIL-v1). The last line shows the difference (model = GPASOIL-v1) - (model = GPASOIL-v0). All plots are in kgP ha^−1^. The effect of changing the soil P dynamic model is provided for different soil P pools (P_i-lab_, P_i-sec_, P_x-occ_) and on the variable f_upns_, which corresponds to the P uptake prescribed by the data but that the soil P pools simulated by the model are not able to satisfy. Soil P pools plotted correspond to the year 2018 while fP_upns_ corresponds to the annual average over 1900–2018.

**Fig. 6:**
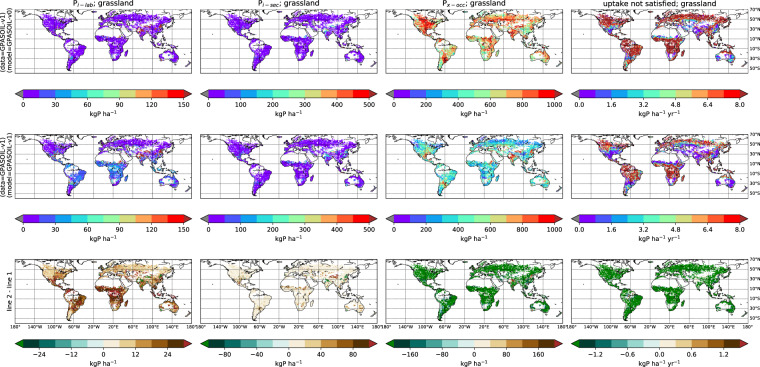
Same as Fig. 5 but applied to grassland.

Here, we prescribed (data = GPASOIL-v0) for the whole section and assessed the effect of using (model = GPASOIL-v1) instead of (model = GPASOIL-v0) (Figs. 5, 6 for cropland and grassland, respectively). Overall, for cropland, compared to (model = GPASOIL-v0), (model = GPASOIL-v1) tends to increase vs. decrease P_i-lab_ in grid-cells where values were very low vs. very high, respectively, making the spatial soil P distribution more homogeneous (Fig. S9). For grassland, (model = GPASOIL-v1) tends to increase P_i-lab_ almost everywhere as compared to (model = GPASOIL-v0) (38 vs 23 kgP ha^−1^, respectively) (Fig. 6). Note that, while P_i-lab_ is not defined equally in (model = GPASOIL-v0) and (model = GPASOIL-v1) (as P_i-sol_ is only distinguished from P_i-lab_ in (model = GPASOIL-v1)), the comparison between the two models can nevertheless focus on P_i-lab_ as the maximum value for the ratio P_i-sol_/P_i-lab_ reaches only 0.2% in (model = GPASOIL-v1). We found that (model = GPASOIL-v1) allows soil P pools simulated to be more consistent with the P plant uptake prescribed (last column of Figs. 5, 6), especially for grassland (Fig. 6).

**Fig. 7:**
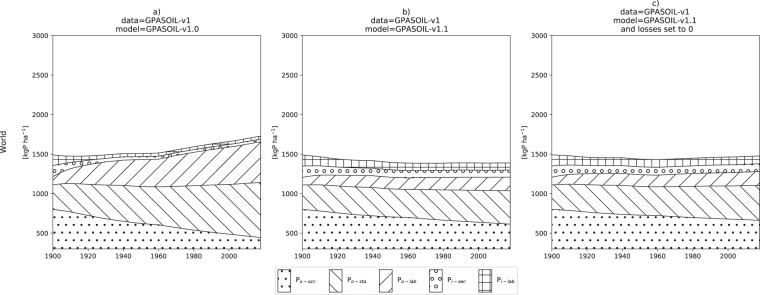
Effect of increased mineralization rates on soil P pools simulated for grassland at the global scale. In panel (a), k_m1_ = 2.7e^−5^day^−1^ and k_m2_ = 2.7e^−4^day^−1^ for respectively P_o-sta_ and P_o-lab_ (i.e. residence time of 100 yr and 10 yr, respectively) following (model = GPASOIL-v0). In panel (b), k_m1_ = 1.8e^−4^day^−1^ and k_m2_ = 1.4e^−3^day^−1^ (i.e. a residence time of 15 yr and 2 yr, respectively). In panel (c), same mineralization rates as in panel (b) are used but in addition, losses by erosion are set to zero.

Soil exchanges with P_x-occ_ play a key-role in the difference between (model = GPASOIL-v0) and (model = GPASOIL-v1). This in particularly true for grassland for which, the model compensates negative soil P budget by P taken from P_x-occ_ (3^rd^ column of Fig. 6) while de-occlusion is not allowed in (model = GPASOIL-v0). The amount of P transferred from P_x-occ_ to other pools (P_i-sec_ then P_i-lab_) in (model = GPASOIL-v1) depends on the mineralization rates of organic pools (P_o-lab_ and P_o-sta_) used in the model. Sensitivity tests showed that the P_x-occ_ for global grassland decreased much less in time if we increased the mineralization rates (i.e. decrease the organic residence times) (Fig. 7). Our soil P dynamic model for organic P is too simplistic (no biochemical mineralization, no link with carbon, no microbial pools^27^) and building a more realistic model based on measurements would require a specific study. As it is likely that the temporal variations of global grassland soil P were small during the 1^st^ half of the 20^th^ century, we chose mineralization rates that allow to approach this global pattern in our simulations, i.e. ${k}^{o-sta\to i-lab}$ = 1.8e^−4^day^−1^ and ${k}^{o-lab\to i-lab}$ = 1.4e^−3^day^−1^ (i.e. a residence time of 15 yr and 2 yr, respectively) (Fig. 7). Such configuration is called GPASOIL-v.1.1. Even with increased mineralization, a decrease of P_x-occ_ over time remained, partly attributed to losses through erosion and to transfers to sustain the prescribed P uptake (Fig. 7c). The effect on the global average of the change in mineralization rate is large (from 149 kgP ha^−1^ for cropland and 38 for grassland with GPASOIL-v1.0 to 187 for cropland and 91 for grassland with GPASOIL-v1.1).

We also found that the global averaged soil P pools were sensitive to the value of ${P}_{c,\infty }$ chosen. The choice of ${P}_{c,\infty }$ has a small effect on the global average for common values of ${P}_{c,\infty }$ (global averages of cropland P_i-lab_ of 149 kgP ha^−1^ for default value of ${P}_{c,\infty }$ (0.1 mgP L^−1^) vs 174 and 148 for kgP ha^−1^ for ${P}_{c,\infty }$ equal to the 1^st^ and 3^rd^ quartile of ${P}_{c,\infty }$ found in Helfenstein *et al*.^25^, respectively; 2^nd^ and 3^rd^ line of Fig. S10) but a large effect for lowest values of ${P}_{c,\infty }$ such as values that could be found in Andosols or Ferralsols (global averaged P_i-lab_ of 239 kgP ha^−1^ for ${P}_{c,\infty }$ = 0.005 mgP L^−1^, 1^st^ line of Fig. S10). This suggest that more work is needed to constrain further spatial variation of ${P}_{c,\infty }$ (see the “Usages Notes” section).

### Best simulations, uncertainty associated and comparison to previous estimates

**Fig. 8:**
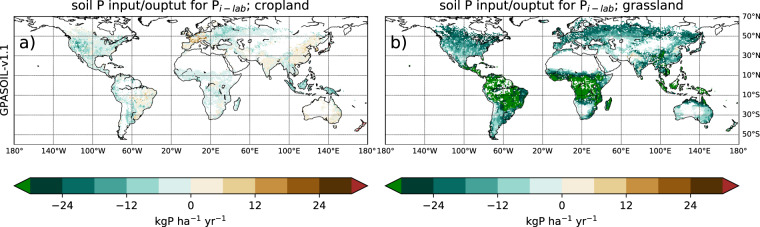
Spatial distribution of the soil P budget (soil P input - output) for P_i-lab_ for both cropland and grassland. Only plant P uptake allowed by the soil P pools simulated was considered in this budget and thus the soil P budget plotted is representative to GPASOIL-v1.1.

**Fig. 9:**
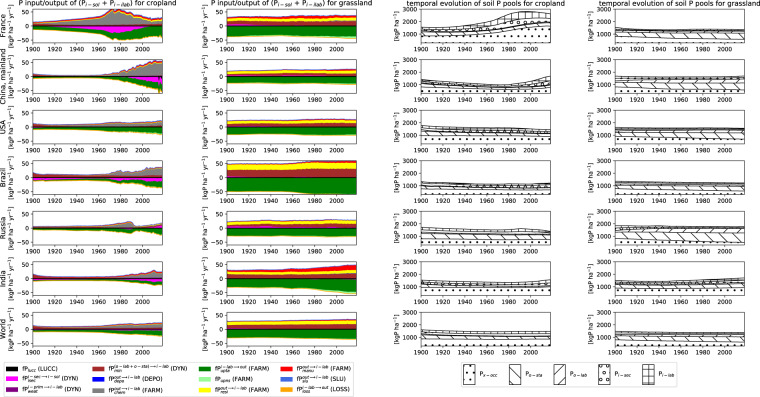
Temporal evolution of soil P_i-lab_ input/output and simulated soil P pools for several (group of) countries and for both cropland and grassland. Simulations used correspond to GPASOIL-v1.1. In left columns, only flux corresponding to soil P dynamics (weathering, mineralization, net flux from P_i-sec_) are simulated while others are prescribed through (data = GPASOIL-v1).

In the following, we focused on the GPASOIL-v1.1 simulations as we considered them as our best estimates. Figure 8 shows the spatial distribution of the soil P budget (soil P input - output) for P_i-lab_. Figure 9 shows the input/output for P_i-lab_ (including soil P input/output and fluxes resulting from soil P dynamics) and soil P pools simulated for GPASOIL-v1.1 for both cropland and grassland for few countries and at the global scale. It underlines the transfer from P_i-lab_ to P_i-sec_ (through P_i-sol_) for countries as France (in 1970s) or China (in the current time period) in cropland, when soil P input are larger than soil P output. Flux from P_i-sec_ to P_i-lab_ was simulated for cropland in Russia or for many countries in grassland. Such flux also occurred for France in the current time-period in cropland but with a smaller magnitude (average of 0.9 kgP ha^−1^ yr^−1^ over 2010–2018 in cropland for France), which corresponds to transfer from P inherited from past fertilizer applications to current plant uptake.

**Fig. 10:**
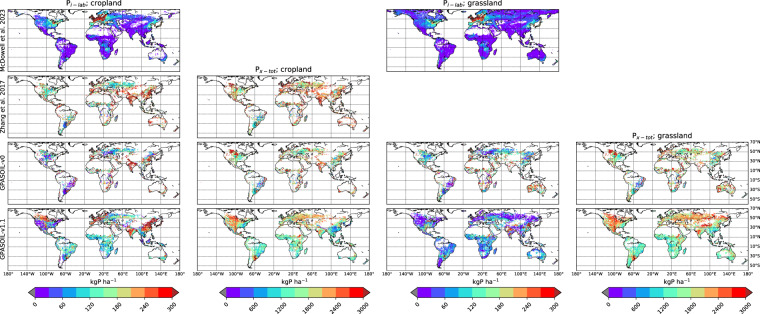
Comparison between previous estimates (McDowell *et al*.^58, Zhang *et al*.^, GPASOIL-v0) and our best estimates (GPASOIL-v1.1) for P_i-lab_ and P_x-tot_ for cropland and grassland when available. Grassland were not simulated in Zhang *et al*.^12^ (and while Sattari *et al*.^82^, based on the same approach, focused on grassland, they did not provide nor discuss the distribution in grasslands). McDowell *et al*.^58^ provided Olsen P concentration for top 0–0.2 m at 1 km^2^ resolution for any land-use categories. We used the land-use information used in their study to filter their estimates and computed soil P for cropland and grassland only before changing the projection and regriding to half-degree resolution. Soil P concentration was converted in kgP ha^−1^ for top 0–0.3 m by using the bulk density used in our study and by assuming that the P concentration is representative to 0-0.3 m top soil layer. Note that P_i-lab_ of McDowell *et al*.^58^ is based on Olsen P extraction while pools design in other estimates plotted in this Figure is based on Hedley fractionation. McDowell *et al*.^58^ did not provide P_x-tot_.

Figure 10 shows the comparison, for the top 0–0.3 m soil layer, between GPASOIL-v1.1 and other published modelling products available: GPASOIL-v0, Zhang *et al*.^12^, and McDowell et al.^58^. In parallel to the studies based on process-based model of soil P dynamics mentioned in the introduction^11,12^, we also included a comparison against a global dataset of soil plant available P based on a statistical model trained on a database of measured soil P concentration^58^. McDowell *et al*.^58^ did not focus on agricultural soils but their dataset encompassed these soils, and we filtered them in the scope of our comparison. GPASOIL-v1.1, GPASOIL-v0 and Zhang *et al*.^12^ provide soil P pools for top 0–0.3 m in kgP ha^−1^ for cropland (and grassland) while McDowell *et al*.^58^ provide soil P concentration for top 0–0.2 m in mgP (kg of soil)^−1^ for any land-use categories at 1 km² resolution. We used the land-use information used in McDowell *et al*.^58^ to filter their estimate and computed soil P for cropland and grassland only, before regriding them to half-degree regular resolution. McDowell *et al*.^58^ provide soil P pools for cropland, rangeland, improved grassland, forest and non-productive areas. According to our definition of grassland, we gathered ‘improved grassland’ and ‘rangeland’ classes of McDowell *et al*.^58^ to compare to our grassland soil P pools estimates. Then, we convert soil P concentration in kgP ha^−1^ for top 0–0.3 m by using the bulk density used in our study and by assuming that the P concentration provided by McDowell *et al*.^58^ is also representative to 0–0.3 m.

Global average of P_i-lab_ decreased for both cropland and grassland from GPASOIL-v0 to GPASOIL-v1.1. The relative decrease was even larger for grassland (from 208 to 91 kgP ha^−1^) than for cropland (from 253 to 187 kgP ha^−1^). P_i-lab_ reached 238 kgP ha^−1^ in Zhang *et al*.^12^ for cropland, and 62 and 32 kgP ha^−1^ in McDowell *et al*.^58^ for cropland and grassland, respectively. P_i-lab_ provided by McDowell *et al*.^58^ is difficult to compare to other estimates used here as it is based on Olsen P extraction vs. pool design based on Hedley fractionation used in other studies. For cropland, we simulated larger P in South America in GPASOIL-v1.1 than in GPASOIL-v0, a lower P content in Africa in GPASOIL-v1.1 than in GPASOIL-v0 while some patterns remains stable in the two versions (high soil P in India and China, low P content in West Europe and Russia).

Differences in the global averaged soil P between GPASOIL-v1.1 and McDowell *et al*.^58^ are likely to be caused by the shallower depth used in McDowell *et al*.^58^ and the different P fractions measured (Olsen P) or estimated (P_i-lab_). The effect of difference in soil depth considered is not straightforward, depending on the farming practices (tillage, fertilization, etc.^61^). However, we do know the likely effect of the Olsen-P vs P_i-lab_. Labile inorganic P from Hedley is close to the Colwell P test used in Australia; both use a 16 hours soil extraction with 0.5 M ${{\rm{HCO}}}_{{3}^{-}}$. There are equations to estimate Colwell P from Olsen-P while these equations differ according to soil P sorption capacity (e.g., Colwell-P is 2.869 × Olsen-P - 2.93 for non-calcareous soils and 1.376 × Olsen-P + 8.80 for calcareous soils)^62^. After converting the values derived from McDowell *et al*.^58^ for (more common) non-calcareous soils, we get 165 and 81 kgP ha^−1^, for cropland and grassland respectively; meaning that the global values calculated in McDowell *et al*.^58^ are within the same range as P_i-lab_ simulated here.

As the biogeochemical background was found to be a major driver of the spatial distribution of P_x-tot_^11^, and because the same biogeochemical background was considered for both cropland and grassland, P_x-tot_ was similar between cropland and grassland for GPASOIL-v1.1. The same reasoning could be applied to GPASOIL-v0. P_x-tot_ for cropland shows also some similarities between Zhang *et al*.^12^ and GPASOIL-v0 as both relied on the same dataset to approach the initial soil P content^4^. Differences in P_x-tot_ between GPASOIL-v0 and GPASOIL-v1.1 were linked to differences in P_x-tot_ of unmanaged soil used in each study (Yang *et al*.^4^ for v0 vs. He *et al*.^14^ for v1).

**Fig. 11:**
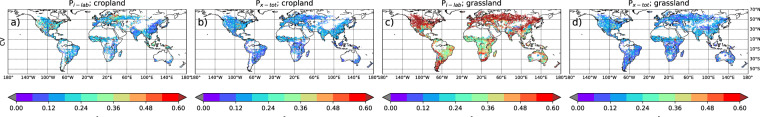
Coefficients of variation (CV) for P_i-lab_ and P_x-tot_ for both cropland and grassland. CV was computed by using mean and standard-deviation among 100 simulations performed to assess how the uncertainty in the driver estimates has an effect on the soil P pools simulated. In the 100 simulations used for this plot, the uncertainty of all drivers was considered.

The coefficients of variation for GPASOIL-v1.1 for P_i-lab_ and P_x-tot_ were plotted in Fig. 11 for both cropland and grassland. These coefficients were due to uncertainty in drivers estimates only. Uncertainty was larger for P_i-lab_ than for P_x-tot_ as the uncertainty in BIOG explained exclusively the uncertainty in P_x-tot_ while all drivers contributed to the uncertainty in P_i-lab_. Uncertainty of P_x-tot_ was similar for both cropland and grassland (global averaged coefficient of variation of 0.17) while much larger for grassland (0.54) than for cropland (0.22) for P_i-lab_. This was explained by the large uncertainty in plant uptake due to uncertainty in both grassland NPP and P concentration of grass. Using mean values for variables related to FARM (instead of random ones) makes the global averaged coefficients of variation of P_i-lab_ to decrease up to 0.14 for cropland and 0.24 for grassland, underlying the large contribution of FARM uncertainty in uncertainty of simulated P_i-lab_.

### Evaluation of the simulated soil P pools at different scales (country, continental/watershed, global scale)

The evaluation of predicted soil P is challenging due to lack of data compiling Hedley P measurements from cropland/grassland soils at the global scale. Different chemical extraction protocols are used worldwide with difficulties to find correspondence among different extractions. In addition, some chemical extraction in cropland/grassland soils at long-term field sites can be found in the literature but these sites usually encompass different P treatments (varying within their amount/rate/nature of fertilizer) with difficulties to link with farming practices occurring in the region/grid-cell including the sites. Here we relied on 3 regional datasets based on soil monitoring networks that carried out measurements on soil samples: RMQS over France^63^, LUCAS over Europe^19^, and STS over USA/Canada^64^. They differ in the chemical extraction used, the spatial extent and resolution, and the distinction (or not) between cropland and grassland. Characteristics of each dataset is given in Table 12.

**Table 12:** Information about the regional dataset used to evaluate the soil P distribution simulated in our study (GPASOIL-v1.1).

Name	RMQS	LUCAS	STS
Reference	^Jolivet *et al*.63^	^Jones *et al*.19^	^TFI64^
Website	Data can be download from: 10.15454/QSXKGA	Data can be downloaded after a request at ESDA website^80^: https://esdac.jrc.ec.europa.eu/content/lucas2015-topsoil-data	http://soiltest.tfi.org.
Spatial extent	France	Europe	Canada and USA
Years of sampling	1^st^ compaign of measurement (2002–2009)	2015	Different years were available in STS but we used the last year in common to GPASOIL-v1.1 and STS (i.e. 2015).
Soil layer	Value of the 1^st^ soil layer considered was used (this soil layer is not necessary exactly equal to 0–30 cm but very close to it)	0–0.2 m	Value for the top 0–0.3 m was computed by using the P concentration available for each soil layer within this horizon in STS.
Chemical extraction for P	Olsen P	Olsen P (information given in Tóth *et al*.^81^)	All measures were done using different P extraction method and then all data were converted to Bray and Kurtz P1 Equivalent.
Land-cover used to approach cropland and grassland	The categories “cropland” and “fruits” for the land-cover “occupation1” were used for our land-cover cropland. The category “grassland” was used for our land-cover “grassland”.	Categories “cropland” and “grassland” for the “main group of Land Cover” (LC0) were considered	Values are provided at county/province without distinction between cropland and grassland
Unit	gP_2_O_5_ (kg of soil)^−1^	mgP (kg of soil)^−1^	mgP (kg of soil)^−1^
Treatments performed for comparison to our dataset	- change in projection - average of all sites within 0.5 grid-cell (with average done separately for cropland and grassland) - conversion in kgP ha^−1^ by using the 0.5 resolution bulk density and volumetric fraction of coarse fragments used in our approach	- average of all sites within 0.5 grid-cell (with average done separately for cropland and grassland) - conversion in kgP ha^−1^ by using the 0.5 resolution bulk density and volumetric fraction of coarse fragments used in our approach	- depth average to compute 0–0.3 m value - conversion in kgP ha^−1^ by using the country-scale value of bulk density that we computed from the 0.5 resolution bulk density and volumetric fraction of coarse fragments used in our approach

For both RMQS and LUCAS, we averaged all sites within each 0.5 grid-cell separately for cropland and grassland, by considering land-uses types of each dataset (Table 12). For both RMQS and LUCAS, a detection limit for P-Olsen measurement was given in the dataset description (5 mgP (kg of soil)^−1^ for RMQS, 10 mgP (kg of soil)^−1^ for LUCAS) but we did not exclude the sites below this threshold to prevent bias in our grid-cell averages. All dataset (RMQS, LUCAS, STS) provided soil P in terms of concentration and we converted them in kgP ha^−1^ by using the half-degree grid-cell bulk density and volumetric fraction of coarse fragments used previously in our study and by considering the P concentration given in each dataset representative to the top 0–0.3 m. The soil P content at half-degree resolution in kgP ha^−1^ from the 3 datasets were provided in Figs. S11–S13.

**Fig. 12:**
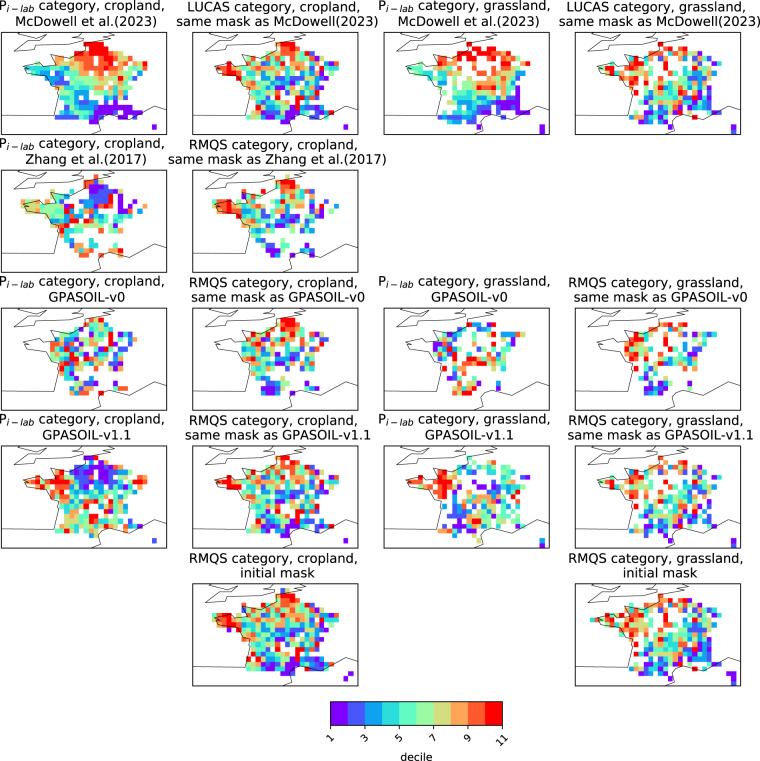
Comparison between RMQS and either McDowell *et al*.^58^, Zhang *et al*.^12^, GPASOIL-v0 or GPASOIL-v1.1. RMQS compiled Olsen P with a cropland vs grassland distinction over France. The comparison focuses on the distribution in deciles as the simulations and RMQS were not based on same chemical extraction. All RMQS sites within half-degree grid-cell were averaged, with a distinction between cropland and grassland. Years used for the modelling approach to perform the comparison are given in Table 13. As the number of grid-cells vary between the modelling approach of McDowell *et al*.^58^, Zhang *et al*.^12^, GPASOIL-v0 or GPASOIL-v1.1, we masked the RMQS dataset according to the mask of each modelling approach output. Note that both RMQS and McDowell *et al*.^58^ are based on Olsen P.

**Fig. 13:**
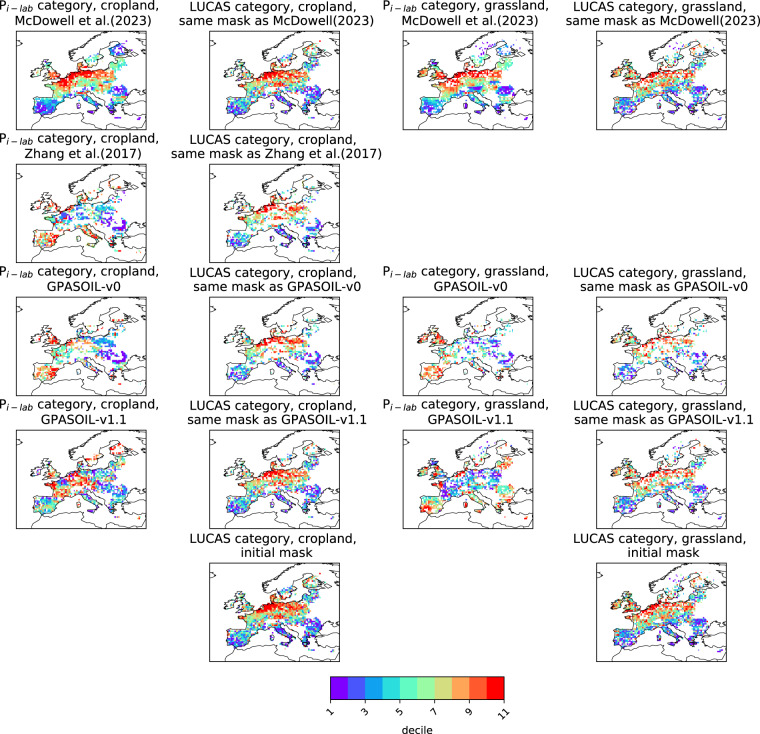
Comparison between LUCAS and either McDowell *et al*.^58^, Zhang *et al*.^12^, GPASOIL-v0 or GPASOIL-v1.1. LUCAS compiled Olsen P with a cropland vs grassland distinction over Europe. The comparison focuses on the distribution in deciles as the simulations and LUCAS were not based on same chemical extraction. All LUCAS sites within half-degree grid-cell were averaged, with a distinction between cropland and grassland. Years used for the modelling approach to perform the comparison are given in Table 13. As the number of grid-cells vary between the modelling approach of McDowell *et al*.^58^, Zhang *et al*.^12^, GPASOIL-v0 or GPASOIL-v1.1, we masked LUCAS according to the mask of each modelling approach output. Note that LUCAS was part of the data source used in McDowell *et al*.^58^ to train their statistical model.

**Fig. 14:**
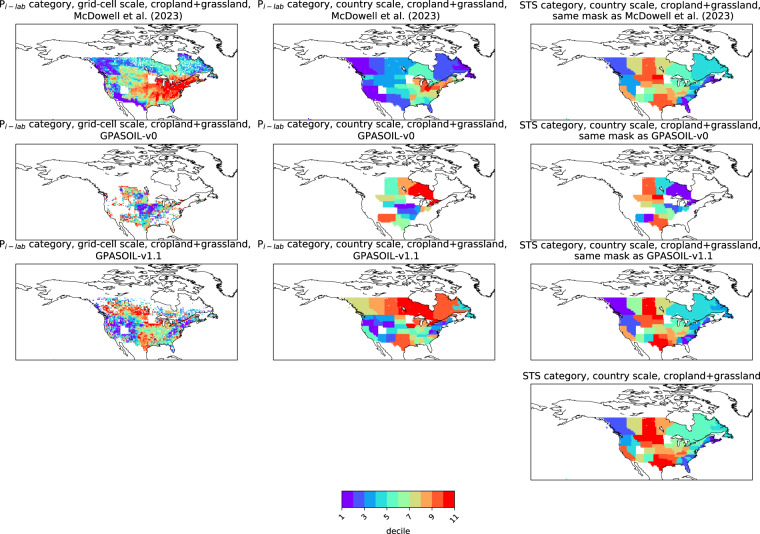
Comparison between STS and either McDowell *et al*.^58^, GPASOIL-v0 or GPASOIL-v1.1. State/province averages were performed on simulations to allow the comparison to STS. The comparison focuses on the distribution in deciles as the simulations and STS were not based on the same chemical extraction. Note that McDowell *et al*.^58^, GPASOIL-v0, GPASOIL-v1.1 are not representative to the same year (Table 13). We excluded from the comparison the states/provinces for which our simulation does not provide 75% of the land in farm for the considered states/province.

As the datasets and our simulation did not rely on the same chemical extraction (pools representative to Hedley extraction in our simulation vs Olsen P for RMQS and LUCAS vs Bray and Kurtz P1 Equivalent for STS, Table 12), we focused our evaluation on the comparison of the relative spatial distribution. To do so, we first mapped our simulation and each dataset by using deciles (Figs. 12–14). As the number of grid-cells considered vary between our GPASOIL-v0 and GPASOIL-v1.1, we masked the grid-cells in the dataset that are excluded from our simulations (Figs. 12–14). Spearman correlation was computed on the absolute value (i.e. not in decile but in kgP ha^−1^) and provided in Table 13. In the scope to compare our simulation to STS, we computed first state/province scale values from our simulation before computing relative distribution with decile. We excluded from the comparison the states/provinces for which our simulation does not provide 75% of the land in farm for the considered state/province. The treatment of our simulation to compare it to STS was plotted in Fig. S14. The year used in our simulation for the comparison to the different datasets was the exact year of the dataset for GPASOIL-v1.1 (2002–2009 average for RMQS, 2015 for LUCAS, 2015 for STS) and 2005 for GPASOIL-v0 (as it is the only one year available in this latter). The comparison distinguished cropland and grassland when possible (RMQS and LUCAS).

**Table 13:** Spearman correlations computed between the different dataset (RMQS, LUCAS, STS) and P_i-lab_ simulated by McDowell *et al*.^58^, Zhang *et al*.^12^, GPASOIL-v0 or GPASOIL-v1.1.

Dataset used for the comparison	Spatial extent and years of sampling	Modeling approach	Years used in the modeling approach to compare with the dataset	Spearman correlation over cropland	Spearman correlation over grassland	Spearman correlation over cropland + grassland
RMQS	France 2002–2009	^McDowell *et al*.58^	2009	**+0.33 (p-value = 0.00)**	**+0.36 (p-value = 0.00)**	
		^Zhang *et al*.12^	2010	−0.09 (p-value = 0.27)		
		GPASOIL-v0	2005	+0.03 (p-value = 0.77)	−0.11 (p-value = 0.27)	
		GPASOIL-v1.1	2002–2009	+0.01 (p-value = 0.94)	+**0.32 (p-value = 0.00)**	
LUCAS	France 2015	^McDowell *et al*.58^	2009	+**0.39 (p-value = 0.00)**	+**0.56 (p-value = 0.00)**	
		^Zhang *et al*.12^	2010	+0.01 (p-value = 0.92)		
		GPASOIL-v0	2005	+0.04 (p-value = 0.68)	**−0.22 (p-value = 0.02)**	
		GPASOIL-v1.1	2015	−0.01 (p-value = 0.89)	+0.07 (p-value = 0.34)	
LUCAS	Europe 2015	^McDowell *et al*.58^	2009	+**0.56 (p-value = 0.00)**	+**0.58 (p-value = 0.00)**	
		^Zhang *et al*.12^	2010	**−0.07 (p-value = 0.03)**		
		GPASOIL-v0	2005	+0.05 (p-value = 0.11)	**−0.10 (p-value = 0.01)**	
		GPASOIL-v1.1	2015	+**0.18 (p-value = 0.00)**	**−0.13 (p-value = 0.00)**	
STS	Canada and USA 2015	^McDowell *et al*.58^	2009			−0.20 (p-value = 0.35)
		GPASOIL-v0	2005			−0.23 (p-value = 0.35)
		GPASOIL-v1.1	2015			+**0.32 (p-value = 0.03)**

Bold front is used for p-value ≤ 0.05.

Overall, we found that GPASOIL-v1.1 had higher correlation than GPASOIL-v0 for grassland over France when using RMQS (+0.32 for GPASOIL-v1.1 vs non-significant correlation for GPASOIL-v0), for cropland over Europe (+0.18 for GPASOIL-v1.1 vs non-significant correlation for GPASOIL-v0, both by using LUCAS) and for Canada/USA (no distinction between cropland and grassland) (+0.32 for GPASOIL-v1.1 vs non-significant correlation for GPASOIL-v0). In particular, GPASOIL-v1.1 was able to catch the higher soil P content in grassland of West of France (Fig. 12). However, a similar agreement was not found between GPASOIL-v1.1 and LUCAS after extracting French sites from LUCAS (Table 13 and Fig. S15). This can be explained by a relatively low spearman correlation between RMQS and LUCAS over France for grassland (0.47; p-value = 0.00). GPASOIL-v1.1 was also able to catch the high soil P content in states/provinces of the north centre and south centre of the USA and Canada.

The update from GPASOIL-v0 to GPASOIL-v1.1 did not improve the correlation with datasets neither for cropland in France nor for grassland in Europe (Table 13). In particular, GPASOIL-v1.1 simulated a low soil P content in cropland of north of France contrary to what was found in RMQS. A low soil P content was also found in the north of France in GPASOIL-v0 (Fig. 12). In GPASOIL-v1.1, this pattern was explained by a large P uptake of root crops (sugar beet, potatoes) in that region. Overall, the soil P pools simulated in north of Europe are sensitive to root crop parameters related to uptake and residues. GPASOIL-v1.1 has some difficulties to catch the increasing soil P along a south-east to north-west gradient found in both cropland and grassland at the European scale in LUCAS (Fig. 13). In particular, the spearman correlation between LUCAS and GPASOIL-v1.1 was significantly negative for grassland (−0.13, p-value = 0.00, Table 13). GPASOIL-v1.1 had also difficulties to reproduce low soil P in East and West of Canada (Fig. 14).

Correlations between McDowell *et al*.^58^ and regional soil P datasets are higher than correlations found with GPASOIL-v1.1 for both RMQS and LUCAS, but not for STS. The high correlations between McDowell *et al*.^58^ and LUCAS is explained by the fact that LUCAS was one the data source used to train the statistical model in McDowell *et al*.^58^. Also, the fact that both RMQS and McDowell *et al*.^58^ are based on Olsen P can contribute to explain the high correlation between them. Difficulties of McDowell *et al*.^58^ to match STS (that did not provide separate values for cropland vs grassland) could be partly explained by the fact that we considered all “rangeland” pixels in McDowell *et al*.^58^ as grassland, contributing to unrealistic contribution of rangeland soil P pools to agricultural pools at states/provinces scale.

At that stage, it is difficult to understand if the mismatch between GPASOIL-v1.1 and the different soil P dataset was explained by misrepresentation of some drivers (in particular FARM or SPRO), incorrect soil P dynamics modeling or uncertainties related to the use of the different dataset for the comparison (see next Section).

## Usages Notes

The soil P pools available in the GPASOIL-v1.1 dataset are model estimates and not free from error due to imperfect modeling (e.g. soil organic P dynamics), inaccurate/uncertainties in inputs (e.g. use of dataset about N for manure to derive P in manure, bulk density not representative to agricultural soils, etc.), limitation in the estimate of some drivers (e.g. difficulty to represent P uptake in grassland), misreporting in agricultural census statistics at the basis of few input (e.g. crop P uptake), and constant in space information (e.g. soil plough layer). Only few sources of uncertainty are considered in our approach (uncertainty related to the datasets describing the drivers to provide the coefficients of variation, uncertainty related to the mineralization rates for GPASOIL-v1.0 and GPASOIL-v1.1).

Caution is particularly required if the user wants to focus on a restricted geographical area (e.g. ≤ continent) of the estimates.

The variables used as input of the soil P dynamic model (the so-called drivers) provided with the soil P pools simulated thanks to our approach should not be used separately to the soil P pools and the users should instead use original dataset if he.she wants to focus on a given driver.

It is worth noting that GPASOIL-v1.1 does not exactly match the spatial distribution given by regional soil P dataset. This mismatch can be explained by misrepresentation of some drivers (in particular FARM or SPRO), incorrect soil P dynamics modeling or uncertainties related to the use of the different dataset for the comparison. About the misrepresentation of some drivers, we made use of the fact that both RMQS and LUCAS provide, in addition to soil P used in the evaluation of our simulations, information about pH, soil organic carbon, and soil texture, i.e. soil properties that are involved in our soil P dynamics parameterization (SPRO driver). Figure S16 shows the comparison for these soil properties between Soilgrids 2.0 (used in our approach), RMQS and LUCAS. Some spatial patterns are not consistent between Soilgrids 2.0, RMQS and LUCAS (e.g. pH). Also, the comparison lets suggest that we would need to distinguish cropland vs grassland for soil parameters involved in the soil P dynamics model.

To improve our understanding of the mismatch between GPASOIL-v1.1 and soil P dataset, it could also be particularly interesting to investigate if the correlation between GPASOIL-v1.1 and LUCAS would be improved in case of simulations over Europe where both soil P input/output and soil dynamics parameterization were improved. European simulation of GPASOIL-v1.1 could be forced by soil P input/output of Panagos *et al*.^65^. How the correlation with LUCAS evolved in sensitivity tests where some crop-specific parameters related to uptake and residues (in particular for root crops) are made varying should also be studied. Soil P dynamics could be improved for European simulations as LUCAS provide information about soil oxides, which can allow to use initial parameterizations of Wang *et al*.^18^ (instead of modified ones as descried in Table 10) and in particular spatially varying concentration of P in soil solution at steady-state (${P}_{c,\infty }$). Also, it could be interesting to evaluate if the soil P dynamic model, even without being fed by oxide concentrations, can match the known global distribution of soil phosphorus retention potential^66^. Finally, the extension of dataset such as LUCAS or RMQS with Hedley fractionation measurements would also allow a more straightforward comparison to modelling studies.

### Supplementary information

Supplementary Table S1

Supplementary Information

## Data Availability

The scripts of the modelling approach described in this paper was made available at recherche.data.gouv.fr^57^. Our code is available within the GPASOIL_scripts.tgz file along the tgz file that contain the GPASOIL-v1.0 and GPASOIL-v1.1 soil P pools. GPASOIL_scripts.tgz contains the whole file tree and associated scripts that were used to generate GPASOIL-v1_output.tgz. The directory GPASOIL/ contains few sub-directories: - GrabData_and_PrepDriver/ that contains all scripts (bash and python) used to generate the input of the soil P dynamic model. These input correspond to the different drivers described in the current manuscript. The input files generated (in netcdf format) are within the directory output_prepDriver/ and are here provided. The procedures to download the original dataset (if this dataset is available on the web) used to generate the input files and information are given for each driver in specific README.txt files. - main/ contains the soil P dynamic model. Different version are provided: (model = v0), (model = v1.0) and (model = v1.1). The 2nd one (i.e. (model = v1.0)) was used to generate GPASOILv1.0 while the latter (i.e. (model = v1.1)) was used to generate GPASOILv1.1 - evaluation/ contains the script to compare the model output to regional databases. The databases are not provided. General information about the procedure to get the regional databases are provided in a specific README.txt file. - output_main/ is the directory that receives the different soil P maps simulated. A container generated with Singularity 3 was also provided and allows users to run the different scripts on a server or local computer without issue of incompatibility about Ubuntu distribution or Python packages. Modeling and analysis were performed in using Python (Python Software Foundation. Python Language Reference, version 3.6.10., available at http://www.python.org, last access: January 2020).

## References

[1] Hou, E. *et al*. Global meta-analysis shows pervasive phosphorus limitation of aboveground plant production in natural terrestrial ecosystems. *Nat. Commun*. **11**, (2020). 10.1038/s41467-020-14492-w PMC6994524 32005808

[2] Kvakić M (2018). Glob. Biogeochem. Cycles.

[3] Carpenter SR (2005). Eutrophication of aquatic ecosystems: Bistability and soil phosphorus. Proc. Natl. Acad. Sci..

[4] Yang X, Post WM, Thornton PE, Jain A (2013). The distribution of soil phosphorus for global biogeochemical modeling. Biogeosciences.

[5] Achat DL, Pousse N, Nicolas M, Brédoire F, Augusto L (2016). Soil properties controlling inorganic phosphorus availability: general results from a national forest network and a global compilation of the literature. Biogeochemistry.

[6] He X (2021). Global patterns and drivers of soil total phosphorus concentration. Earth Syst. Sci. Data.

[7] Elser J, Bennett E (2011). A broken biogeochemical cycle. Nature.

[8] MacDonald GK, Bennett EM, Potter PA, Ramankutty N (2011). Agronomic phosphorus imbalances across the world’s croplands. Proc. Natl. Acad. Sci. USA.

[9] Demay J, Ringeval B, Pellerin S, Nesme T (2023). Half of global agricultural soil phosphorus fertility derived from anthropogenic sources. Nat. Geosci..

[10] Sattari SZ, Bouwman AF, Giller KE, van Ittersum MK (2012). Residual soil phosphorus as the missing piece in the global phosphorus crisis puzzle. Proc. Natl. Acad. Sci. USA.

[11] Ringeval B (2017). Phosphorus in agricultural soils: drivers of its distribution at the global scale. Glob. Change Biol..

[12] Zhang J (2017). Spatiotemporal dynamics of soil phosphorus and crop uptake in global cropland during the 20th century. Biogeosciences.

[13] Bouwman, L. *et al*. Exploring global changes in nitrogen and phosphorus cycles in agriculture induced by livestock production over the 1900–2050 period. *Proc. Natl. Acad. Sci. USA* **52**, 10.1073/pnas.1012878108 (2013). 10.1073/pnas.1012878108 PMC3876211 21576477

[14] He X (2023). Global patterns and drivers of phosphorus fractions in natural soils. Biogeosciences.

[15] Wang YP, Law RM, Pak B (2010). A global model of carbon, nitrogen and phosphorus cycles for the terrestrial biosphere. Biogeosciences.

[16] Helfenstein J (2018). Combining spectroscopic and isotopic techniques gives a dynamic view of phosphorus cycling in soil. Nat. Commun..

[17] Helfenstein J (2020). Estimates of mean residence times of phosphorus in commonly considered inorganic soil phosphorus pools. Biogeosciences.

[18] Wang, Y. *et al*. Toward a Global Model for Soil Inorganic Phosphorus Dynamics: Dependence of Exchange Kinetics and Soil Bioavailability on Soil Physicochemical Properties. *Glob. Biogeochem. Cycles* **36**, (2022). 10.1029/2021GB007061 PMC9286372 35865755

[19] Jones, A., Fernandez-Ugalde, O. & Scarpa, S. *LUCAS 2015 topsoil survey: presentation of dataset and results*. vol. 10.2760/616084, JRC121325 (EUR 30332 EN, Publications Office of the European Union: Luxembourg, 2020).

[20] Chini L (2021). Land-use harmonization datasets for annual global carbon budgets. Earth Syst. Sci. Data.

[21] Poggio L (2021). SoilGrids 2.0: producing soil information for the globe with quantified spatial uncertainty. SOIL.

[22] Bronick CJ, Lal R (2005). Soil structure and management: a review. Geoderma.

[23] Klein Goldewijk K, Beusen A, Doelman J, Stehfest E (2017). Anthropogenic land use estimates for the Holocene – HYDE 3.2. Earth Syst. Sci. Data.

[24] Krinner G (2005). A dynamic global vegetation model for studies of the coupled atmosphere-biosphere system. Glob. Biogeochem. Cycles.

[25] Helfenstein J, Jegminnat J, McLaren TL, Frossard (2018). Soil solution phosphorus turnover: derivation, interpretation, and insights from a global compilation of isotope exchange kinetic studies. Biogeosciences.

[26] Buendía C, Kleidon A, Porporato A (2010). The role of tectonic uplift, climate, and vegetation in the long-term terrestrial phosphorous cycle. Biogeosciences.

[27] McConnell CA, Kaye JP, Kemanian AR (2020). Reviews and syntheses: Ironing out wrinkles in the soil phosphorus cycling paradigm. Biogeosciences.

[28] Beck MA, Sanchez PA (1996). Soil phosphorus movement and budget after 13 years of fertilized cultivation in the Amazon basin. Plant Soil.

[29] Sun Y (2021). Global evaluation of the nutrient-enabled version of the land surface model ORCHIDEE-CNP v1.2 (r5986). Geosci. Model Dev..

[30] Zhang B (2017). Global manure nitrogen production and application in cropland during 1860–2014: a 5 arcmin gridded global dataset for Earth system modeling. Earth Syst. Sci. Data.

[31] Xu, R. *et al*. Increased nitrogen enrichment and shifted patterns in the world’s grassland: 1860–2016, 13, *Earth Syst. Sci. Data*, **11**, 175–187, 10.5194/essd-11-175-2019 (2019).

[32] Lun F (2018). Global and regional phosphorus budgets in agricultural systems and their implications for phosphorus-use efficiency. Earth Syst. Sci. Data.

[33] Velthof, G. L., Bannink, A. & Oenema, O. Relationships between animal nutrition and manure quality.

[34] Lu C, Tian H (2017). Global nitrogen and phosphorus fertilizer use for agriculture production in the past half century: shifted hot spots and nutrient imbalance. Earth Syst. Sci. Data.

[35] Sun Y (2017). Diagnosing phosphorus limitations in natural terrestrial ecosystems in carbon cycle models. Earths Future.

[36] Wang J (2019). Vegetation type controls root turnover in global grasslands. Glob. Ecol. Biogeogr.

[37] Zhang X, Wang W (2015). The decomposition of fine and coarse roots: their global patterns and controlling factors. Sci. Rep..

[38] Smith B (2014). Implications of incorporating N cycling and N limitations on primary production in an individual-based dynamic vegetation model. Biogeosciences.

[39] Monfreda C, Ramankutty N, Foley JA (2008). Farming the planet: 2. Geographic distribution of crop areas, yields, physiological types, and net primary production in the year 2000: GLOBAL CROP AREAS AND YIELDS IN 2000. Glob. Biogeochem. Cycles.

[40] Sun, Y. *et al*. Field‐Based Estimation of Net Primary Productivity and Its Above‐ and Belowground Partitioning in Global Grasslands. *J. Geophys. Res. Biogeosciences* **126**, (2021).

[41] Kastner, T. *et al*. Land use intensification increasingly drives the spatiotemporal patterns of the global human appropriation of net primary production in the last century. *Glob. Change Biol*. gcb.15932, 10.1111/gcb.15932 (2021). 10.1111/gcb.15932 34651392

[42] Wang Y (2018). GOLUM-CNP v1.0: a data-driven modeling of carbon, nitrogen and phosphorus cycles in major terrestrial biomes. Geosci. Model Dev..

[43] Lun F (2021). Influences of international agricultural trade on the global phosphorus cycle and its associated issues. Glob. Environ. Change.

[44] Smil V (2000). Phosphorus in the environment: natural flows and human interferences. Annu. Rev. Energy Environ..

[45] Zechmeister-Boltenstern S (2015). The application of ecological stoichiometry to plant–microbial–soil organic matter transformations. Ecol. Monogr..

[46] Spohn M (2020). Increasing the organic carbon stocks in mineral soils sequesters large amounts of phosphorus. Glob. Change Biol..

[47] Bentsen NS, Felby C, Thorsen BJ (2014). Agricultural residue production and potentials for energy and materials services. Prog. Energy Combust. Sci..

[48] Ye Y (2014). Carbon, Nitrogen and Phosphorus Accumulation and Partitioning, and C:N:P Stoichiometry in Late-Season Rice under Different Water and Nitrogen Managements. PLoS ONE.

[49] Wang R (2015). Significant contribution of combustion-related emissions to the atmospheric phosphorus budget. Nat. Geosci..

[50] Mahowald N (2008). Global distribution of atmospheric phosphorus sources, concentrations and deposition rates, and anthropogenic impacts. Glob. Biogeochem. Cycles.

[51] Wang R (2017). Global forest carbon uptake due to nitrogen and phosphorus deposition from 1850 to 2100. Glob. Change Biol..

[52] van Puijenbroek PJTM, Beusen AHW, Bouwman AF (2019). Global nitrogen and phosphorus in urban waste water based on the Shared Socio-economic pathways. J. Environ. Manage..

[53] Donatello S, Cheeseman CR (2013). Recycling and recovery routes for incinerated sewage sludge ash (ISSA): A review. Waste Manag..

[54] Borrelli, P. *et al*. An assessment of the global impact of 21st century land use change on soil erosion. *Nat. Commun*. **8**, (2017). 10.1038/s41467-017-02142-7 PMC5722879 29222506

[55] van den Hurk B (2016). LS3MIP (v1.0) contribution to CMIP6: the Land Surface, Snow and Soilmoisture Model Intercomparison Project – aims, setup and expected outcome. Geosci. Model Dev..

[56] Valade A (2014). Modeling sugarcane yield with a process-based model from site to continental scale: uncertainties arising from model structure and parameter values. Geosci. Model Dev..

[57] Ringeval B (2023). Recherche Data Gouv..

[58] McDowell RW, Noble A, Pletnyakov P, Haygarth PM (2023). A Global Database of Soil Plant Available Phosphorus. Sci. Data.

[59] Iizumi T, Sakai T (2020). The global dataset of historical yields for major crops 1981–2016. Sci. Data.

[60] Senthilkumar K, Nesme T, Mollier A, Pellerin S (2012). Conceptual design and quantification of phosphorus flows and balances at the country scale: The case of France. Glob. Biogeochem. Cycles.

[61] Tian J (2017). Accumulation and distribution of phosphorus in the soil profile under fertilized grazed pasture. Agric. Ecosyst. Environ..

[62] Moody PW, Speirs SD, Scott BJ, Mason SD (2013). Soil phosphorus tests I: What soil phosphorus pools and processes do they measure?. Crop Pasture Sci..

[63] Jolivet, C., Boulonne, L. & Ratié, C. Manuel du réseau de Mesures de la Qualité des Sols. *Unité InfoSol - INRA Orléans - Fr*. (2006).

[64] TFI. Soil Test Levels in North America, 2020 Summary Update. (2020).

[65] Panagos P (2022). Improving the phosphorus budget of European agricultural soils. Sci. Total Environ..

[66] Batjes, N. H. Global distribution of soil phosphorus retention potential.

[67] Bouwman, A. F. & Kram. *Integrated modelling of global environmental change: an overview of IMAGE 2.4*. (Netherlands Environmental Assessment Agency, 2006).

[68] Xu, R. *et al*. Half-degree gridded manure and fertilizer nitrogen inputs in global grassland systems during 1860–2016, PANGAEA, 10.1594/PANGAEA.892940 (2018).

[69] Demay, J. *Supplementary Information - Half of global agricultural soil phosphorus fertility derived from anthropogenic sources*. 10.57745/LEPJCS (2023).

[70] Kastner, T. *et al*. Data supplement for ‘Land use intensification increasingly drives the spatiotemporal patterns of the global human appropriation of net primary production in the last century’, 10.5281/ZENODO.5519104 (2022). 10.1111/gcb.15932 34651392

[71] Lu, C. & Tian, H. Half-degree gridded nitrogen and phosphorus fertilizer use for global agriculture production during 1900-2013, 10.1594/PANGAEA.863323 (2016).

[72] Zhang, B. *et al*. Manure nitrogen production and application in cropland and rangeland during 1860 - 2014: A 5-minute gridded global data set for Earth system modeling, 10.1594/PANGAEA.871980 (2017).

[73] Klein Goldewijk, Dr. ir. C.G.M. Anthropogenic land-use estimates for the Holocene; HYDE 3.2. DANS. 10.17026/dans-25g-gez3 (Utrecht University, 2017).

[74] Van Oost K (2007). The Impact of Agricultural Soil Erosion on the Global Carbon Cycle. Science.

[75] Hurtt GC (2011). Harmonization of land-use scenarios for the period 1500–2100: 600 years of global gridded annual land-use transitions, wood harvest, and resulting secondary lands. Clim. Change.

[76] Chini, L. *et al*. *Land-Use Harmonization Datasets for Annual Global Carbon Budgets*. https://essd.copernicus.org/preprints/essd-2020-388/essd-2020-388.pdf, 10.5194/essd-2020-388 (2021).

[77] Decharme B, Martin E, Faroux S (2013). Reconciling soil thermal and hydrological lower boundary conditions in land surface models: LOWER BOUNDARY CONDITIONS OF SOIL IN LSM. J. Geophys. Res. Atmospheres.

[78] He X (2023). Global patterns and drivers of phosphorus pools in natural soils version 2.0. 24653728 Bytes.

[79] Yang X, Post WM (2011). Phosphorus transformations as a function of pedogenesis: A synthesis of soil phosphorus data using Hedley fractionation method. Biogeosciences.

[80] Panagos, P. *et al*. European Soil Data Centre 2.0: Soil data and knowledge in support of the EU policies. *Eur. J. Soil Sci*. **73** (2022).

[81] Tóth G, Guicharnaud R-A, Tóth B, Hermann T (2014). Phosphorus levels in croplands of the European Union with implications for P fertilizer use. Eur. J. Agron..

[82] Sattari SZ, Bouwman AF, Martinez Rodríguez R, Beusen AHW, van Ittersum MK (2016). Negative global phosphorus budgets challenge sustainable intensification of grasslands. Nat. Commun..

