# Supplementary material for: A global dataset on phosphorus in agricultural soils
Source: Sci Data. 2024 Jan 2;11:17. doi: 10.1038/s41597-023-02751-6 (PMC10762041; doi:10.1038/s41597-023-02751-6)
Supplement: Supplementary file 1 — Supplementary Table S1 [file 41597_2023_2751_MOESM1_ESM.pdf]

Table S1: Summary of the strategy used to consider the uncertainty related to the different drivers in (data=GPASOIL-v1) ; i.e. strategy to compute a random value for each variable considered.

Driver name	Land-use considered (if a distinction is needed)	Variables considered	Type of distribution	Characteristics of the distribution	Additional conditions when a random value is chosen
FARM	grassland	P in chemical fertilizer	Normal distribution defined by a mean and a standard-deviation	For each grid-cell, the mean is equal to 0.22 multiplied by N in chemical fertilizer provided by (Xu et al., 2019) and a standard-deviation equal to 25% of the mean was assumed. The value of 25 % was arbitrary chosen.	Random value > 0
FARM	grassland	Total P in manure	Uniform distribution between two estimates	For each grid-cell, the 1st estimate is equal to 0.20 multiplied by N in manure provided by (Xu et al., 2019) and the 2nd estimate is equal to the 1st one multiplied by a scaling factor based on the country-scale estimate of P manure produced by livestock (Demay et al., 2023).	Random value > 0
FARM	grassland	Composition of P in manure	No uncertainty considered		
FARM	grassland	NPP involved in plant uptake computation	Normal distribution with four standard-deviation between two estimates	The two estimates are both based on the (Kastner et al., 2021) spatial distribution but with global average of either (Kastner et al., 2021) or (Sun et al., 2021).	Random value > 0
FARM	grassland	P concentration of aboveground plant involved in the computation of P plant uptake	Normal distribution with four standard-deviation between two estimates	First estimate of $2.5e^{-2}$ gP $(100gFM)^{-1}$ (Ref (Wang et al., 2018)) and second estimate of $1.5e^{-1}$ gP $(100gFM)^{-1}$ (Ref (Lun et al., 2021)).	Random value > $2.5e^{-2}$ gP $(100gFM)^{-1}$
FARM	grassland	Total P in residues	No uncertainty considered (i.e. for each grid-cell, once the random value for P uptake was computed, P in residues was deduced by keeping the same (P uptake : P residues) ratio as with the mean values of uptake and residue).		
FARM	grassland	Composition of P in residues	Uniform between two values	Two estimates equal to the mean - 50% and the mean + 50%, with a mean composition of 0.4 (inorganic labile), 0.4 (organic labile), 0.2 (stable organic).	Ensure consistency between the different fractions
FARM	cropland	P in chemical fertilizer	No uncertainty considered		
FARM	cropland	Total P in manure	Uniform distribution between two estimates	For each grid-cell, the 1st estimate equal to 0.20 multiplied by N in manure provided by (Zhang et al., 2017) and the 2nd estimate equal to 1st one multiplied by a scaling factor based on the country-scale estimate of P manure produced by livestock (Demay et al., 2023).	Random value > 0
FARM	cropland	Composition of P in manure	No uncertainty considered		
FARM	cropland	P plant uptake	Normal distribution defined by a mean and a standard-deviation	For each grid-cell, the mean is equal to the value computed following Eq.37 and a standard-deviation equal to 25% of the mean was assumed. The value of 25 % was arbitrary chosen.	Random value > 0
FARM	cropland	Total P in residues	No uncertainty considered	For each grid-cell, once the random value for P uptake was computed, P in residues was deduced by keeping the same (P uptake : P residues) ratio as with the mean values of uptake and residues.	
FARM	cropland	Composition of P in residues	Uniform between two estimates	Two estimates equal to the mean - 50% and the mean + 50%, with a mean composition of 0.4 (inorganic labile), 0.4 (organic labile), 0.2 (stable organic).	Ensure consistency between the different fractions
DEPO		Total P in atmospheric deposition	Normal distribution defined by a mean and a standard-deviation	For each grid-cell, the mean is equal to the value derived from combination of (Wang et al., 2015) and (Wang et al., 2017), and a standard-deviation equal to 60% of the mean was assumed. The value of 60 % was derived from values provided at the global scale by (Wang et al., 2017).	Random value > 0
DEPO		Composition of P in atmospheric deposition	No uncertainty considered (i.e. once the random value for P total deposition was computed, the contribution of each source (mineral dust, and others) to the total deposition was deduced by keeping the same contribution as the one computed with the mean value).		
SLUD		Total P in sludges	Normal distribution defined by a mean and a standard-deviation	For each grid-cell, the mean is equal to the value derived from (van Puijenbroek et al., 2019) and (Demay et al., 2023), and a std equal to 15% of the mean was assumed. The value of 15 % was arbitrary chosen.	Random value > 0
SLUD		Composition of P in sludges	No uncertainty considered		
LOSS		Fraction of soil lost per erosion	Normal distribution defined by a mean and a standard-deviation	For each grid-cell, the mean is equal to the mean value provided by (Borrelli et al., 2017) and a standard-deviation equal to 16% of the mean was considered. The value of 16 % corresponds to the upper uncertainty range found in (Borrelli et al., 2017). Note : in (Borrelli et al., 2017), the uncertainty was not centered : -6.68% +15.6%:	Random value > 0
LUCC		Land fractions and land transitions	No uncertainty considered		
CLIM		Near-surface air temperature, soil temperature and soil water content (absolute and relative to the field capacity)	Normal distribution defined by a mean and a standard-deviation	For each grid-cell, the mean and standard-deviation were computed by using 9 CMIP-6 simulations.	The random value varies between the spatial minimum and spatial maximum of the mean value.
SPRO		Soil texture, soil water pH, and soil carbon concentration	Normal distribution with 3.75 standard-deviation between two estimates	For each grid-cell, the two estimates correspond to the 5% and 95 % quantiles provided by Soilgrids 2.0 (Poggio et al., 2021)	The random value varies between the spatial minimum of the 5% quantile and the spatial maximum of the 95% quantile
BIOG		Total P in unmanaged soils	Normal distribution defined by a mean and a standard-deviation	For each grid-cell, the mean is equal to the mean value provided by (He et al., 2023) and the standard-deviation was approached by the standard-error provided by (He et al., 2023). The standard-error is preferred here, instead of the standard-deviation, as with random forest (as used to generate the dataset in (He et al., 2023)), the standard-error is a measure of the probability of the true value while the standard-deviation is a measure of the probability of samples which increases with the number of trees used in the random forest. The standard-error of the 0.1-0.2m was used to approach the standard-error of the 0-0.3m horizon.	The random value varies between (the spatial minimum – spatial mean of the standard-error) and (the spatial maximum + the spatial mean of the standard-error).
BIOG		Contribution of each pool to the total P in unmanaged soils	No uncertainty considered (i.e. the contribution of each pool to the total soil P is kept the same for each random value as the contribution to the mean).		

References:

Borrelli, P., Robinson, D. A., Fleischer, L. R., Lugato, E., Ballabio, C., Alewell, C., Meusburger, K., Modugno, S., Schütt, B., Ferro, V., Bagarello, V., Oost, K. V., Montanarella, L., and Panagos, P.: An assessment of the global impact of 21st century land use change on soil erosion, Nat. Commun., 8, https://doi.org/10.1038/s41467-017-02142-7, 2017.

Demay, J., Ringeval, B., Pellerin, S., and Nesme, T.: Half of global agricultural soil phosphorus fertility derived from anthropogenic sources, Nat. Geosci., 16, 69–74, https://doi.org/10.1038/s41561-022-01092-0, 2023.

He, X., Augusto, L., Goll, D. S., Ringeval, B., Wang, Y.-P., Helfenstein, J., Huang, Y., and Hou, E.: Global patterns and drivers of phosphorus fractions in natural soils, Biogeosciences, 20, 4147–4163, https://doi.org/10.5194/bg-20-4147-2023, 2023.

Kastner, T., Matej, S., Forrest, M., Gingrich, S., Haberl, H., Hickler, T., Krausmann, F., Lasslop, G., Niedertscheider, M., Plutzar, C., Schwarzmüller, F., Steinkamp, J., and Erb, K.: Land use intensification increasingly drives the spatiotemporal patterns of the global human appropriation of net primary production in the last century, Glob. Change Biol., gcb.15932, https://doi.org/10.1111/gcb.15932, 2021.

Lun, F., Sardans, J., Sun, D., Xiao, X., Liu, M., Li, Z., Wang, C., Hu, Q., Tang, J., Ciais, P., Janssens, I. A., Obersteiner, M., and Peñuelas, J.: Influences of international agricultural trade on the global phosphorus cycle and its associated issues, Glob. Environ. Change, 69, 102282, https://doi.org/10.1016/j.gloenvcha.2021.102282, 2021.

Poggio, L., de Sousa, L. M., Batjes, N. H., Heuvelink, G. B. M., Kempen, B., Ribeiro, E., and Rossiter, D.: SoilGrids 2.0: producing soil information for the globe with quantified spatial uncertainty, SOIL, 7, 217–240, https://doi.org/10.5194/soil-7-217-2021, 2021.

van Puijenbroek, P. J. T. M., Beusen, A. H. W., and Bouwman, A. F.: Global nitrogen and phosphorus in urban waste water based on the Shared Socio-economic pathways, J. Environ. Manage., 231, 446–456, https://doi.org/10.1016/j.jenvman.2018.10.048, 2019.

Sun, Y., Feng, Y., Wang, Y., Zhao, X., Yang, Y., Tang, Z., Wang, S., Su, H., Zhu, J., Chang, J., and Fang, J.: Field-Based Estimation of Net Primary Productivity and Its Above- and Belowground Partitioning in Global Grasslands, J. Geophys. Res. Biogeosciences, 126, https://doi.org/10.1029/2021JG006472, 2021.

Wang, R., Balkanski, Y., Boucher, O., Ciais, P., Peñuelas, J., and Tao, S.: Significant contribution of combustion-related emissions to the atmospheric phosphorus budget, Nat. Geosci., 8, 48–54, https://doi.org/10.1038/ngeo2324, 2015.

Wang, R., Goll, D., Balkanski, Y., Hauglustaine, D., Boucher, O., Ciais, P., Janssens, I., Penuelas, J., Guenet, B., Sardans, J., Bopp, L., Vuichard, N., Zhou, F., Li, B., Piao, S., Peng, S., Huang, Y., and Tao, S.: Global forest carbon uptake due to nitrogen and phosphorus deposition from 1850 to 2100, Glob. Change Biol., 23, 4854–4872, https://doi.org/10.1111/gcb.13766, 2017.

Wang, Y., Ciais, P., Goll, D., Huang, Y., Luo, Y., Wang, Y.-P., Bloom, A. A., Broquet, G., Hartmann, J., Peng, S., Penuelas, J., Piao, S., Sardans, J., Stocker, B. D., Wang, R., Zaehle, S., and Zechmeister-Boltenstern, S.: GOLUM-CNP v1.0: a data-driven modeling of carbon, nitrogen and phosphorus cycles in major terrestrial biomes, Geosci. Model Dev., 11, 3903–3928, https://doi.org/10.5194/gmd-11-3903-2018, 2018.

Xu, R., Tian, H., Pan, S., Dangal, S. R. S., Chen, J., Chang, J., Lu, Y., Skiba, U. M., Tubiello, F. N., and Zhang, B.: Increased nitrogen enrichment and shifted patterns in the world's grassland: 1860–2016, 13, 2019.

Zhang, B., Tian, H., Lu, C., Dangal, S. R. S., Yang, J., and Pan, S.: Global manure nitrogen production and application in cropland during 1860–2014: a 5 arcmin gridded global dataset for Earth system modeling, Earth Syst. Sci. Data, 9, 667–678, https://doi.org/10.5194/essd-9-667-2017, 2017.
