# Supplementary material for: A global dataset on phosphorus in agricultural soils
Source: Sci Data. 2024 Jan 2;11:17. doi: 10.1038/s41597-023-02751-6 (PMC10762041; doi:10.1038/s41597-023-02751-6)
Supplement: Supplementary file 2 — Supplementary Information [file 41597_2023_2751_MOESM2_ESM.pdf]

Supplementary Information of

A global dataset on phosphorus in agricultural soils

B. Ringeval, J. Demay, D.S. Goll, X. He, Y-P. Wang, E. Hou, S. Matej, K-H. Erb, R. Wang, L. Augusto, F. Lun, T. Nesme, P. Borrelli, J. Helfenstein, R.W. McDowell, P. Pletnyakov, S. Pellerin
Scientific Data

Table of contents:

Text S1: Langmuir equation in (model=GPASOIL-v0)
Text S2: Computation of P uptake and P residues as function of the harvested organ
Text S3: Corresponding between grid-cell and country
Text S4: Details of computation of deposition
Text S5: Computation of $frac_{treat}$ for year 1970

Table S1: Summary of the strategy used to consider the uncertainty related to the different drivers in (data=GPASOIL-v1). Cf. Additional document.
Table S2 : Values of (K_S, S_{max}) given by (Wang et al., 2010) and used in (model=GPASOIL-v0) to describe the equilibrium between P_{ISEC} and P_{ILAB}.

Figure S1 : For maize, comparison between the variable finally used in our approach to estimate the yield and the variables at the basis of its computation: the country-scale and temporal varying yield provided by FAOSTAT and the spatially explicit yield distribution for year 2000 given by (Monfreda et al., 2008).
Figure S2 : Time-series of harvested area and yield at country-scale : comparison between the variables finally used in our approach, variables used in our computation and other estimates.
Figure S3: Crop composition of total P uptake at country scale.
Figure S4: P deposition at the global scale derived from a combination between (Wang et al., 2015) and (Wang et al., 2017).
Figure S5 : For a given grid-cell, effect of the time-steps used (1-day vs 6-hours) on the simulated net flux between $P_{i\text{-}sec}$ and $P_{i\text{-}sol}$ and soil P pools.
Figure S6 : Effect of the number of time-steps on simulated $P_{i\text{-}lab}$, $P_{i\text{-}sec}$, $P_{x\text{-}occ}$ for cropland in 2018.
Figure S7 : Spatial distribution of $P_{i\text{-}lab}$ of cropland, $P_{i\text{-}lab}$ of grassland and $P_{x\text{-}tot}$ of cropland simulated in 2005 with (data=GPASOIL-v1, model=GPASOIL-v0) and differences with (data=GPASOIL-v0, model=GPASOIL-v0).
Figure S8 : Soil P budget for $P_{i\text{-}lab}$ of grassland.
Figure S9: Distribution of values of $P_{i\text{-}lab}$ in both cropland and grassland in terms of number of grid-cells.
Figure S10 : Sensitivity of soil P pools simulated in 2018 to $P_{c,\infty}$.
Figure S11: Soil P distribution provided by RMQS after regrid at the half-degree resolution then change in unit.
Figure S12: Soil P distribution provided by LUCAS after regrid at the half-degree resolution then change in unit.
Figure S13: Soil P distribution provided by the STS dataset after change in unit.
Figure S14: Treatment of $P_{i\text{-}lab}$ simulated with GPASOIL-v1.1 to allow the comparison to state/province distribution given by STS.
Figure S15: Same as Fig.13 but focusing on France only.
Figure S16: Comparison between Soilgrids 2.0, RMQS and LUCAS for few soil properties over France.
Figure S17: Strategy to attribute a value to a grid-cell belonging to a country in 2000 for the years before the beginning of the existence of this country.

Text S1: Langmuir equation in (model=GPASOIL-v0)

As reminder, the equilibrium between $P_{i\text{-}sec}$ and $P_{i\text{-}lab}$ in (model=GPASOIL-v0) is based on a Langmuir equation :

if $P_{i\text{-}sec}+P_{i\text{-}lab}<0$, $P_{i\text{-}lab}=P_{i\text{-}sec}=0$ else $$P_{i-sec}=S_{max}\frac{K_S.P_{i-lab}}{1+K_S.P_{i-lab}}$$ (Eq.S1)

where S_{max} is the maximum capacity of secondary minerals to bind P (in kgP ha^{-1}) and K_s is a coefficient (in ha kgP^{-1}). Numerical resolution is done by introducing $P_{i\text{-}sol,lab}=P_{i\text{-}sol}+P_{i\text{-}lab}$ then by solving an 2nd order equation with Pi-sec as unique unknown. Above equation allows to redistribute $P_{i\text{-}sol,lab}$ between $P_{i\text{-}sol}$ and $P_{i\text{-}lab}$ when steady-state is assumed.

Following (Wang et al., 2010), (S_{max}, K_s) varies with the soil order (Table S2). (Wang et al., 2010) calibrated (S_{max}, K_s), so that the resulting P distribution in unmanaged soil matched the dataset described in (Cross and Schlesinger, 1995). 1/Ks corresponds to the parameter called k_{plab} in (Wang et al., 2010) and the derivation of Eq.S1 corresponds to Eq.D9 of (Wang et al., 2010).

Text S2: Computation of P uptake and P residues as function of the harvested organ

We first decomposed the total P uptake in P content of different organs :

$$fP_{uptake}^{i-lab\rightarrow out}(y,c,g)=P_{root}(y,c,g)+P_{abov\backslash harvest}(y,c,g)+P_{harvest}(y,c,g) \quad \text{(Eq.S2)}$$

with P_{root}, $P_{abov\backslash harvest}$ and $P_{harvest}$ corresponding respectively to P in roots, in the aboveground biomass excluding the harvest (also called total residues in (Smil, 2000)), and in the harvest. P in residues is derived by using the fraction of $P_{abov\backslash harvest}$ remaining on the soil (called $frac_{resid}$, the rest being exported from the field) :

$$fP_{resid}^{out\rightarrow x-tot}(y,c,g)=P_{root}(y,c,g)+frac_{resid}(c).P_{abov\backslash harvest}(y,c,g) \quad \text{(Eq.S3)}$$

By injecting the P concentration of each organ considered, Eq.S2 can be written as :

$$fP_{uptake}^{i-lab\rightarrow out}(y,c,g)=\frac{1}{100}. \quad \text{(Eq.S4)}$$
$$[P_{\%,root}(c).F_{root}(y,c,g)+P_{\%,abov\backslash harvest}.F_{abov\backslash harvest}(y,c,g)+P_{\%,harvest}.F_{harvest}(y,c,g)]$$

with $P_{\%,x}$ the P concentration (in gP $(100gFM)^{-1}$) and F_x the fresh matter biomass (in kgFM ha^{-1}) for the organ x with x in {root, abov\harvest, harvest}.

By using the definitions of root:shoot ratio (RSR) and harvest index (HI) (both expressed in dry matter) as well as the biomass in dry matter (DM_x), we can express F_{root} by:

$$F_{root}=\frac{1}{dry_{root}}.DM_{root}=\frac{1}{dry_{root}}.RSR.DM_{shoot}=\frac{1}{dry_{root}}.\frac{RSR}{HI}.DM_{harvest} \quad \text{, i.e.}$$

$$F_{root}=\frac{dry_{harvest}}{dry_{root}}.\frac{RSR}{HI}.F_{harvest} \quad \text{(Eq.S5)}$$

and $F_{abov\backslash harvest}$ by :

$$F_{abov\backslash harvest}=\frac{1}{dry_{abov\backslash harvest}}.DM_{abov\backslash harvest}$$

$$F_{abov\backslash harvest}=\frac{1}{dry_{abov\backslash harvest}}.(DM_{shoot}-DM_{harvest})$$

$$F_{abov\backslash harvest}=\frac{1}{dry_{abov\backslash harvest}}.DM_{harvest}.(\frac{1}{HI}-1) \quad \text{; i.e.}$$

$$F_{abov\backslash harvest}=\frac{dry_{harvest}}{dry_{abov\backslash harvest}}.(\frac{1}{HI}-1).F_{harvest} \quad \text{(Eq.S6).}$$

Reinjecting Eq.S5-S6 in Eq.S4, we found:

$$fP_{uptake}^{i-lab\rightarrow out}(y,c,g)=\frac{1}{100}.F_{harvest}(y,c,g).$$
$$[P_{\%,root}(c).\frac{dry_{harvest}(c)}{dry_{root}(c)}.\frac{RSR(c)}{HI(c)}+P_{\%,abov\backslash harvest}(c).\frac{dry_{harvest}(c)}{dry_{abov\backslash harvest}(c)}.(\frac{1}{HI(c)}-1)+P_{\%,harvest}(c)]$$

Finally, $F_{harvest}$ is the economic yield (expressed in kgFM ha^{-1}), and thus :

$$fP_{uptake}^{i-lab\rightarrow out}(y,c,g)=\frac{Yield(y,c,g)}{100}.$$
$$[P_{\%,root}(c).\frac{dry_{harvest}(c)}{dry_{root}(c)}.\frac{RSR(c)}{HI(c)}+P_{\%,abov\backslash harvest}(c).\frac{dry_{harvest}(c)}{dry_{abov\backslash harvest}(c)}.(\frac{1}{HI(c)}-1)+P_{\%,harvest}(c)]$$

(Eq.S7) (called Eq.39 in the main text).

Similarly, Eq.S3 can be written :

$$fP_{resid}^{out\rightarrow x-tot}(y,c,g)=\frac{Yield(y,c,g)}{100}.$$
$$[P_{\%,root}(c).\frac{dry_{harvest}(c)}{dry_{root}(c)}.\frac{RSR(c)}{HI(c)}+frac_{resid}(c).P_{\%,abov\backslash harvest}(c).\frac{dry_{harvest}(c)}{dry_{abov\backslash harvest}(c)}.(\frac{1}{HI(c)}-1)]$$
(Eq.S8) (called Eq.40 in the main text).

For crops whose the harvest concerns the root (encompassing the category « Root&Tubers » in (Monfreda et al., 2008), « carrot », classified as Vegetable&Melons in (Monfreda et al., 2008) and "sugar beet" classified as "sugar crop" in (Monfreda et al., 2008)), we assumed that the harvest concerns the whole root biomass and Eq.S2 can be replaced by:
$fP_{uptake}^{i-lab\rightarrow out}(y,c,g)=P_{root}(y,c,g)+P_{shoot}(y,c,g)$, i.e.:
$$fP_{uptake}^{i-lab\rightarrow out}(y,c,g)=\frac{1}{100}.[P_{\%,root}(c).F_{root}(y,c,g)+P_{\%,shoot}(c).F_{shoot}(y,c,g)] \qquad \text{(Eq.S9).}$$
For root crop, the definition of HI is different to other crops :
$$HI=\frac{DM_{root}}{DM_{root}+DM_{shoot}} \ .$$
F_{shoot} in Eq.S9 can be either expressed as function of HI or RSR. As RSR of tuber crop in (Monfreda et al., 2008) is not crop-specific but the default value of 0.25 (corresponding to a aboveground fraction of 0.8) provided by (Hicke, 2004) is used instead, we choose to express F_{shoot} as function of HI:
$F_{shoot}=\frac{1}{dry_{shoot}}.DM_{shoot}=\frac{1}{dry_{shoot}}.(\frac{1}{HI}-1).DM_{root}$, i.e.: $F_{shoot}=\frac{dry_{root}}{dry_{shoot}}.(\frac{1}{HI}-1).F_{root}$. By reinjecting this latter equation in Eq.S9 and by considering in addition that Yield=F_{root} for root crop, Eq.S9 becomes:
$$fP_{uptake}^{i-lab\rightarrow out}(y,c,g)=\frac{Yield(y,c,g)}{100}.[P_{\%,root}(c)+P_{\%,shoot}(c).\frac{dry_{root}}{dry_{shoot}}.(\frac{1}{HI}-1)] \qquad \text{(Eq.S10).}$$
As consequence, residues are computed thanks to:
$$fP_{resid}^{out\rightarrow x-tot}(y,c,g)=\frac{Yield(y,c,g)}{100}.frac_{resid}.P_{\%,shoot}(c).\frac{dry_{root}}{dry_{shoot}}.(\frac{1}{HI}-1) \qquad \text{(Eq.S11).}$$
We approached $P_{\%,shoot}$ of Eq.S10-S11 by the P concentration of aboveground residues given by (Lun et al., 2021) (and completed for few crop categories by (Smil, 2000)). $P_{\%,root}$ was approached by the P concentration of harvest and dry_{root} by the dry fraction of harvest, both provided by (Lun et al., 2021). We assumed that dry_{shoot} was equal to dry_{root}. We assumed that $frac_{resid}$ is set to 1 for non-forage root crop and equal to 0 for forage root crop (e.g. « carrot for forage »).

Text S3: Corresponding between grid-cell and country

In our approach, we combined spatially explicit dataset at half-degree resolution and country-scale dataset. The belonging of each grid-cell to a given country was regridded from a 5 arc minutes resolution distribution of M49 code (a unique code for each country) provided by HYDE 3.2 (Klein Goldewijk et al., 2017). This distribution characterized the country boundaries of year 2000 while country spatial boundaries definition can change in time. As we did not have access to spatially explicit maps of M49 for each year within 1900-2018, we needed to make some corresponding between M49 code for each grid-cell of year 2000 to country for any year between 1960 and 2018. This allowed us to provide variables (such as yield, area) to any grid-cells based on country-scale information of a country that does not exist in 2000. To do so, we distinguished two kind of variables (that we called “area-type” and “yield-type”) whose the treatment varied according to the different cases (split of countries, aggregation of countries, etc.) as explained in Fig.S17.

Text S4: Details of computation of deposition

For any year y of the simulation, $D_{dust}(y,g)=D^{A}_{dust}(\overline{2000-2011},g)$, $D_{seasalt}(y,g)=D^{A}_{seasalt}(\overline{2000-2011},g)$ and $D_{PBAP}(y,g)=D^{A}_{PBAP}(2000,g)$. Please, see the variable definition in the Main Text.

To get $D_{natcomb}(y,g)$, we proceed as follows :
for any year y between 1960 and 2007 and any World region reg, $E^{A}_{totcomb}(y,reg)=E^{A}_{natcomb}(y,reg)+E^{A}_{anthcomb}(y,reg)$.
We applied the ratio in emissions to the total combustion deposition : $D_{natcomb}(\overline{1960-2007},g)=D^{A}_{totcomb}(\overline{1960-2007},g)*\frac{E^{A}_{natcomb}(\overline{1960-2007},reg(g))}{E^{A}_{totcomb}(\overline{1960-2007},reg(g))}$
Then, for any year y of the simulation, $D_{natcomb}(y,g)=D_{natcomb}(\overline{1960-2007},g)$.

To get $D_{anthcomb}(y,g)$, we first computed $D_{anthcomb}(\overline{1960-2007},g)$ as follows: $D_{anthcomb}(\overline{1960-2007},g)=D^{A}_{totcomb}(\overline{1960-2007},g)*\frac{E^{A}_{anthcomb}(\overline{1960-2007},reg(g))}{E^{A}_{totcomb}(\overline{1960-2007},reg(g))}$
then proceed differently for years before or after 2007, as explained below.

For any 1960≤y≤2007, $D_{anthcomb}(y,g)=D_{anthcomb}(\overline{1960-2007},g)*\frac{E^{A}_{anthcomb}(y,reg(g))}{E^{A}_{anthcomb}(\overline{1960-2007},reg(g))}$.

For any 2008≤y≤2013, we relied on $D^{B}_{anthcomb}$ computed as follows for any 1997≤y≤2013 : $D^{B}_{anthcomb}(y,g)=min(0,D^{B}_{all}(y,g)-D_{dust}(y,g)-D_{seasalt}(y,g)-D_{PBAP}(y,g)-D_{natcomb}(y,g))$.
Then, for any 2008≤y≤2013, $D_{anthcomb}(y,g)=D^{B}_{anthcomb}(y,g)*\frac{D^{A}_{anthcomb}(\overline{1997-2007},g)}{D^{B}_{anthcomb}(\overline{1997-2007},g)}$.

Finally, for any y≤1959, $D_{anthcomb}(y,g)=D_{anthcomb}(1960,g)$ and for any y≥2014, $D_{anthcomb}(y,g)=D_{anthcomb}(2013,g)$.

Text S5: Computation of $frac_{treat}$ for year 1970

$frac_{treat}$ is available for years 1990, 2000, 2010 and we computed it for 1970 by using the fraction of households connected to sewerage system (SC). SC is slightly above $\sum_{type=1,2,3} frac_{treat}(type)$ as it encompasses the fraction of population connected to sewerage system but whose the sewerage is not treated. SC is available at the World region scale for year 2010 (Table 6 of (van Puijenbroek et al., 2019)) and at the country-scale for years 1990, 2000, 2010 (Table S11 of (van Puijenbroek et al., 2019)) as follows :

$$frac_{treat}(type, country, 1970) = \frac{frac_{treat}(type, country, 1990)}{SC(country, 1990)} * \frac{SC(country, 2010)}{SC(reg(country), 2010)} * SC(reg(country), 1970)$$

As 2010 is the only year in common for SC at both World big region and country scale, we used $\frac{SC(country, 2010)}{SC(reg(country), 2010)} * SC(reg(country), 1970)$ to approach SC at country-scale for 1970.

Table S1: Summary of the strategy used to consider the uncertainty related to the different drivers in (data=GPASOIL-v1) ; i.e. strategy to compute a random value for each variable considered. Cf. additional document.

Table S2 : Values of (K_S, S_{max}) given by (Wang et al., 2010) and used in (model=GPASOIL-v0) to describe the equilibrium between P_{ISEC} and P_{ILAB}.

Soil order	$1/K_S$ (in [kgP ha^{-1}])	S_{max} (in [kgP ha^{-1}])
Alfisol/Spodosol	750	1340
Andisol/Aridisol	780	800
Entisol	640	500
Gelisol/Histosol/Inceptisol	650	770
Mollisol	540	740
Oxisol	100	1450
Ultisol	640	1330
Vertisol	320	320

References

Cross, A. F. and Schlesinger, W. H.: A literature review and evaluation of the. Hedley fractionation: Applications to the biogeochemical cycle of soil phosphorus in natural ecosystems, Geoderma, 64, 197–214, 1995.

Hicke, J. A.: Spatiotemporal patterns of cropland area and net primary production in the central United States estimated from USDA agricultural information, Geophys. Res. Lett., 31, L20502, https://doi.org/10.1029/2004GL020927, 2004.

Iizumi, T. and Sakai, T.: The global dataset of historical yields for major crops 1981–2016, Sci Data, 7, 97, https://doi.org/10.1038/s41597-020-0433-7, 2020.

Klein Goldewijk, K., Beusen, A., Doelman, J., and Stehfest, E.: Anthropogenic land use estimates for the Holocene – HYDE 3.2, Earth Syst. Sci. Data, 9, 927–953, https://doi.org/10.5194/essd-9-927-2017, 2017.

Lun, F., Sardans, J., Sun, D., Xiao, X., Liu, M., Li, Z., Wang, C., Hu, Q., Tang, J., Ciais, P., Janssens, I. A., Obersteiner, M., and Peñuelas, J.: Influences of international agricultural trade on the global phosphorus cycle and its associated issues, Global Environmental Change, 69, 102282, https://doi.org/10.1016/j.gloenvcha.2021.102282, 2021.

Monfreda, C., Ramankutty, N., and Foley, J. A.: Farming the planet: 2. Geographic distribution of crop areas, yields, physiological types, and net primary production in the year 2000: GLOBAL CROP AREAS AND YIELDS IN 2000, Global Biogeochemical Cycles, 22, n/a-n/a, https://doi.org/10.1029/2007GB002947, 2008.

van Puijenbroek, P. J. T. M., Beusen, A. H. W., and Bouwman, A. F.: Global nitrogen and phosphorus in urban waste water based on the Shared Socio-economic pathways, Journal of Environmental Management, 231, 446–456, https://doi.org/10.1016/j.jenvman.2018.10.048, 2019.

Ringeval, B., Augusto, L., Monod, H., van Apeldoorn, D., Bouwman, L., Yang, X., Achat, D. L., Chini, L. P., Van Oost, K., Guenet, B., Wang, R., Decharme, B., Nesme, T., and Pellerin, S.: Phosphorus in agricultural soils: drivers of its distribution at the global scale, Global Change Biology, 23, 3418–3432, https://doi.org/10.1111/gcb.13618, 2017.

Smil, V.: Phosphorus in the environment: natural flows and human interferences, Annual review of energy and the environment, 25, 53–88, 2000.

Wang, R., Balkanski, Y., Boucher, O., Ciais, P., Peñuelas, J., and Tao, S.: Significant contribution of combustion-related emissions to the atmospheric phosphorus budget, Nature Geoscience, 8, 48–54, https://doi.org/10.1038/ngeo2324, 2015.

Wang, R., Goll, D., Balkanski, Y., Hauglustaine, D., Boucher, O., Ciais, P., Janssens, I., Penuelas, J., Guenet, B., Sardans, J., Bopp, L., Vuichard, N., Zhou, F., Li, B., Piao, S., Peng, S., Huang, Y., and Tao, S.: Global forest carbon uptake due to nitrogen and phosphorus deposition from 1850 to 2100, Global Change Biology, 23, 4854–4872, https://doi.org/10.1111/gcb.13766, 2017.

Wang, Y. P., Law, R. M., and Pak, B.: A global model of carbon, nitrogen and phosphorus cycles for the terrestrial biosphere, Biogeosciences, 7, 2261–2282, https://doi.org/10.5194/bg-7-2261-2010, 2010.

Supplementary Figures

Figure S1 : For maize, comparison between the variable finally used in our approach to estimate the yield (Yield) and the variables at the basis of its computation: the country-scale and temporal varying yield provided by FAOSTAT (and extended in time according to population, $Yield_{FAO,bis}$) and the spatially explicit yield distribution for year 2000 given by (Monfreda et al., 2008) ($Yield_{Monfreda}$). The independent yield estimate provided by (Iizumi and Sakai, 2020) was also plotted (1st line). The comparison between the different products has been made by distinguishing few time-periods. (corresponding to the different columns). In this figure, the country-scaled value of $Yield_{FAO,bis}$ has been attributed to all grid-cells belonging to the same country (whatever if cropland occurs or not in these grid-cells). Yields are expressed in (t of fresh matter) ha^{-1}. For this plot, the crop “maize” of (Iizumi and Sakai, 2020) has been considered (while “maize_major” and “maize_second” also exist) and crop considered for Yield is “maize” while “maize for forage” also exist in (Monfreda et al., 2008).

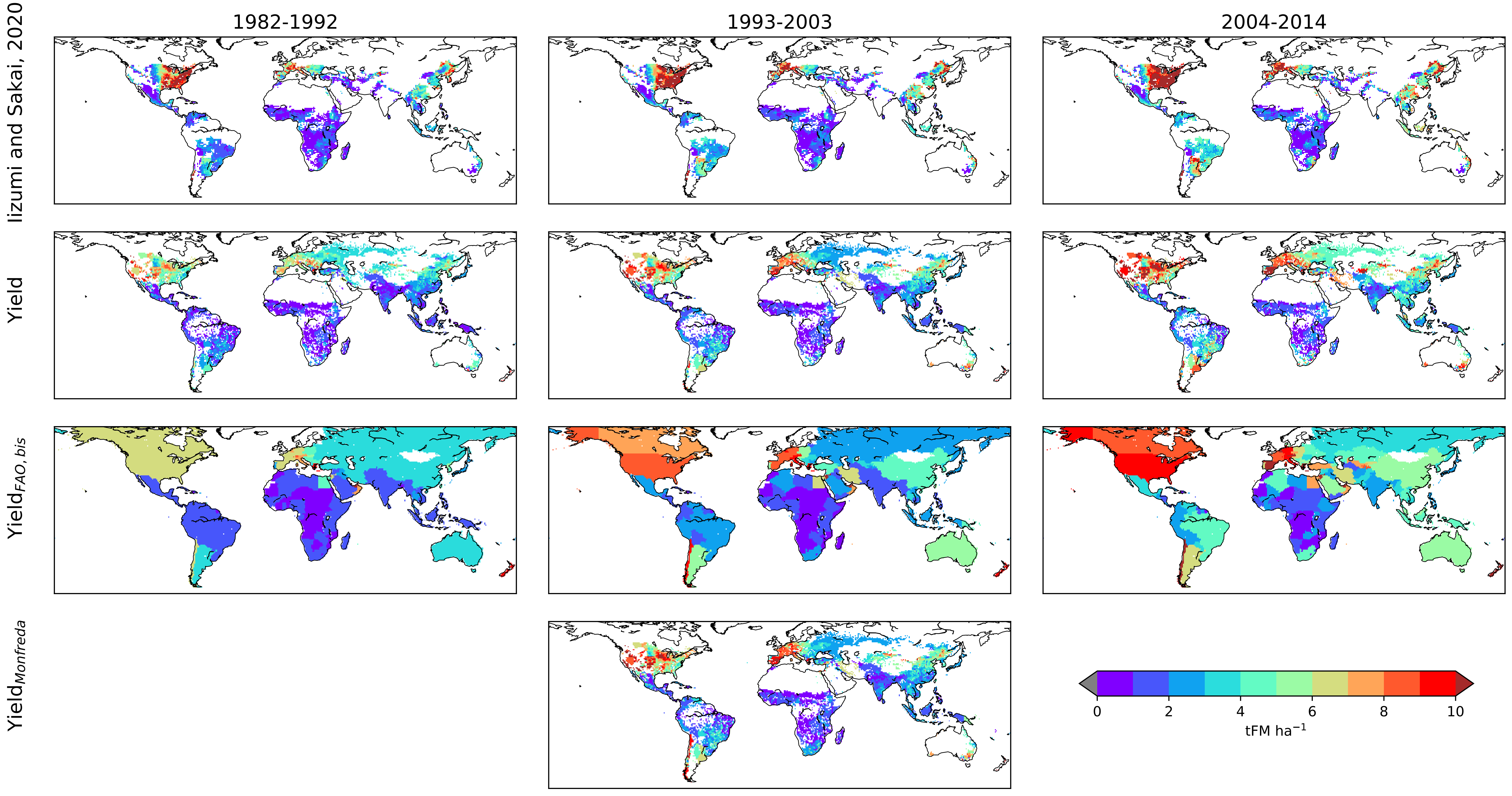

1982-1992
1993-2003
2004-2014
Iizumi and Sakai, 2020
Yield
Yield$_{FAO, bis}$
Yield$_{Monfreda}$
0
2
4
6
8
10
tFM ha^{-1}

Figure S2 : Time-series of harvested area and yield at country-scale : comparison between the variables finally used in our approach (Area, Yield ; in black), variables used in our computation ((Monfreda et al., 2008) and FAOSTAT) and other estimates ((Iizumi and Sakai, 2020) and LUCC). The comparison focuses on maize (left columns) and on the broad crop category encompassing maize in LUCC (« c4ann ») (right columns). This comparison was done at country-scale while the interest of the approach described above is to get a spatially-explicit and temporal-varying distribution of yield (see spatially explicit maps in Fig.S1).

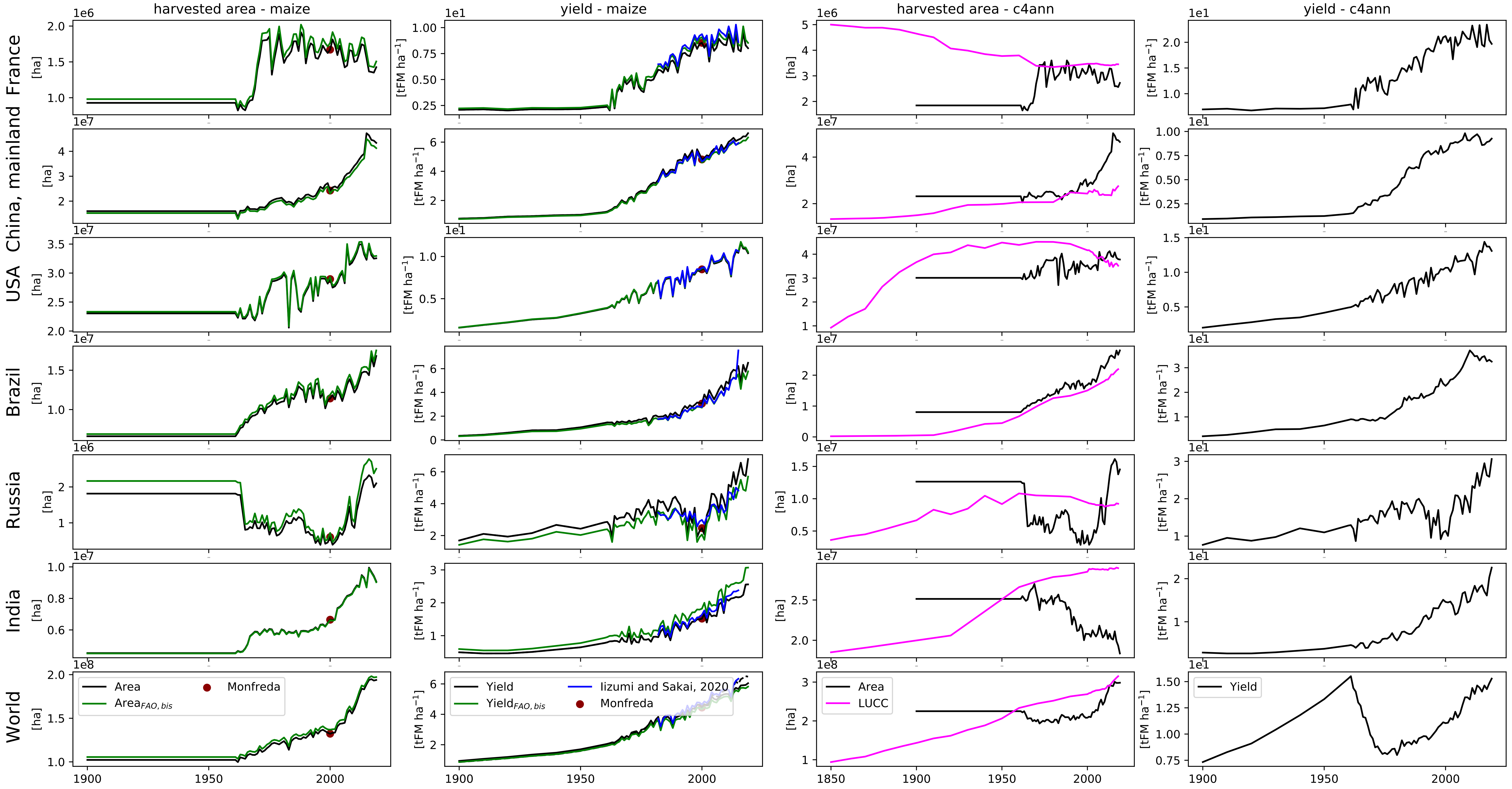

harvested area - maize
yield - maize
harvested area - c4ann
yield - c4ann
France
China, mainland
USA
Brazil
Russia
India
World
[ha]
[tFM ha^{-1}]
Area
Area$_{FAO, bis}$
Monfreda
Yield
Yield$_{FAO, bis}$
Iizumi and Sakai, 2020
LUCC

Figure S3: Crop composition of total P uptake (i.e. $fP_{upta,mean}^{i-lab \rightarrow out}(crop)$) at country scale. Only crop whose the contribution to the total crop P uptake is greater than 5% at least one year is distinguished. Other crops are summed in the category "other" (in yellow). Crop composition of the total crop area (Area(crop)) is also given in the right column. The sum of the area of the crop not considered in our approach as they were missing in FAOSTAT (but existing in (Monfreda et al., 2008) dataset for year 2000) are represented with a black bar. Dash curve in left column corresponds to the total crop area (without distinction in the different crop) provided for the driver LUCC in our study. Crop categories are named following (Monfreda et al., 2008) with « maizefor » corresponding to « maize for forage » and « vegetablenes » to « Vegetables Fresh, other ».

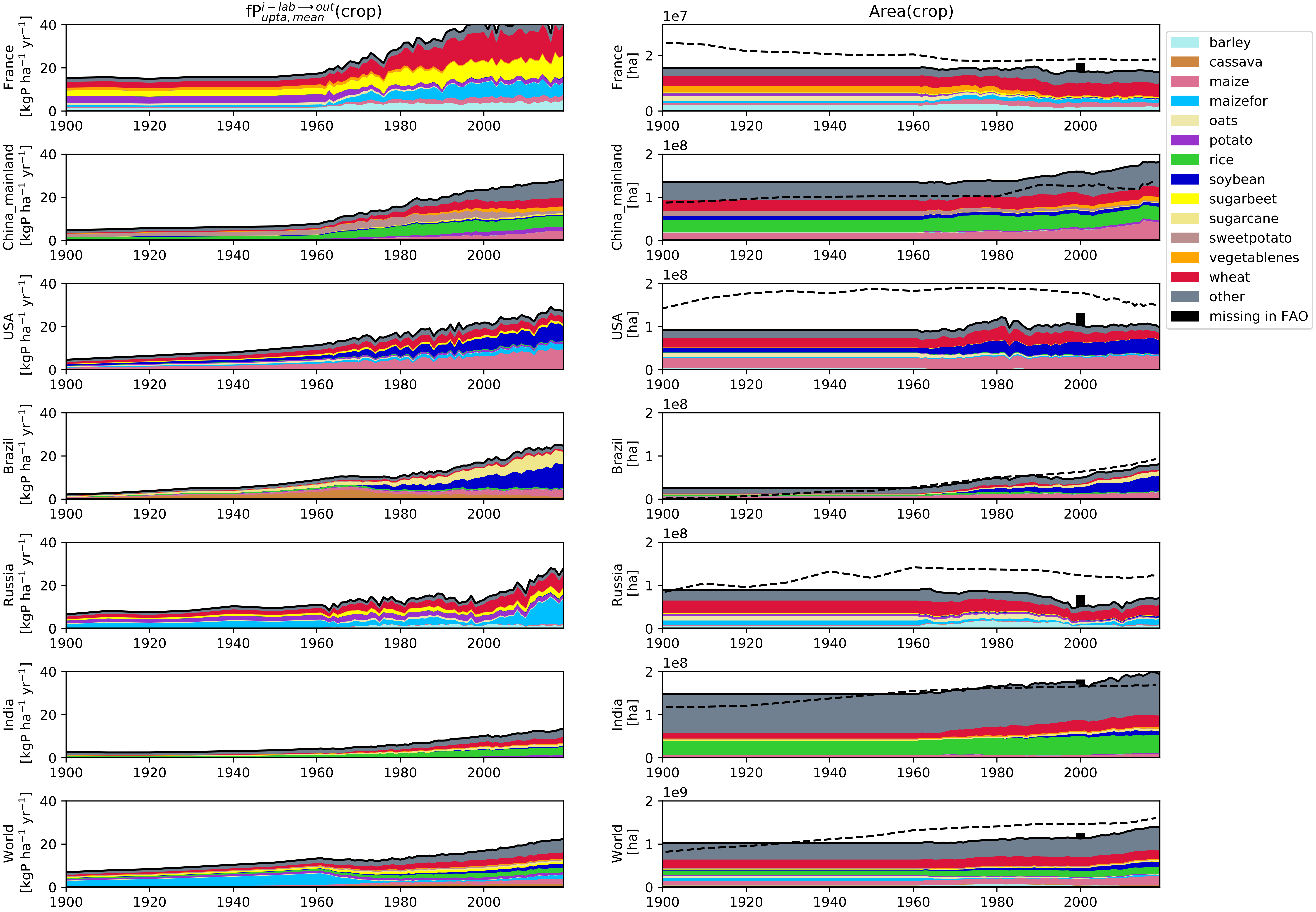

$fP^{i-lab\longrightarrow out}_{upta,mean}$(crop)
Area(crop)
France [kgP ha^{-1} yr^{-1}]
China_mainland [kgP ha^{-1} yr^{-1}]
USA [kgP ha^{-1} yr^{-1}]
Brazil [kgP ha^{-1} yr^{-1}]
Russia [kgP ha^{-1} yr^{-1}]
India [kgP ha^{-1} yr^{-1}]
World [kgP ha^{-1} yr^{-1}]
France [ha]
China_mainland [ha]
USA [ha]
Brazil [ha]
Russia [ha]
India [ha]
World [ha]
1e7
1e8
1e9
barley
cassava
maize
maizefor
oats
potato
rice
soybean
sugarbeet
sugarcane
sweetpotato
vegetablenes
wheat
other
missing in FAO

Figure S4: P deposition at the global scale derived from a combination between (Wang et al., 2015) and (Wang et al., 2017). The panel (a) shows the comparison of global P deposition from anthropogenic combustion comparison between (Wang et al., 2015) (A), (Wang et al., 2017) (B) and the values used here ($D_{anthcomb}$, dot black). The panel (b) displays the composition of total P deposition in cropland (the temporal change in $D_{seasalt}$, D_{dust}, D_{PBAP}, and $D_{natcomb}$ are only attributed to change in cropland extent (through LUCC)).

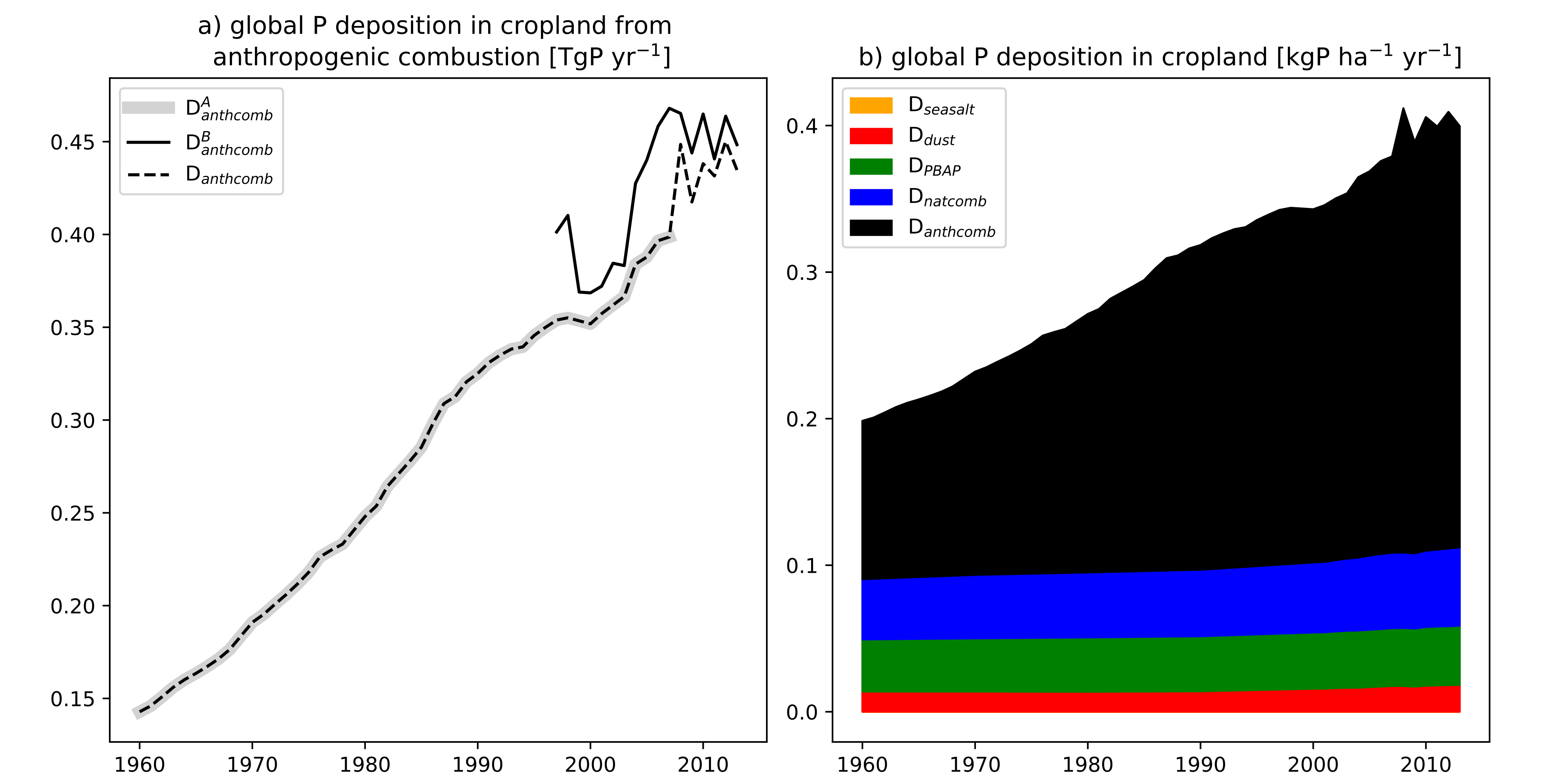

a) global P deposition in cropland from anthropogenic combustion [TgP yr^{-1}]
$D^A_{anthcomb}$
$D^B_{anthcomb}$
$D_{anthcomb}$
0.15
0.20
0.25
0.30
0.35
0.40
0.45
1960
1970
1980
1990
2000
2010
b) global P deposition in cropland [kgP ha^{-1} yr^{-1}]
$D_{seasalt}$
D_{dust}
D_{PBAP}
$D_{natcomb}$
$D_{anthcomb}$
0.0
0.1
0.2
0.3
0.4
1960
1970
1980
1990
2000
2010

Figure S5 : For a given grid-cell (latitude=31.25°N, longitude=106.75°E), effect of the time-steps used (1-day vs 6-hours) on the simulated net flux between $P_{i\text{-}sec}$ and $P_{i\text{-}sol}$ ($fP_{desorp}^{i-sec\rightarrow i-sol} - fP_{sorp}^{i-sol\rightarrow i-sec}$, solid lines in panel (a)) and soil P pools (panels (b) and (c)). Soil P budget for $P_{i\text{-}lab}$ (i.e. inorganic labile fraction of chemical fertilizer + manure + residues + deposition – uptake – losses) was also plotted on panel (a) (dot lines).

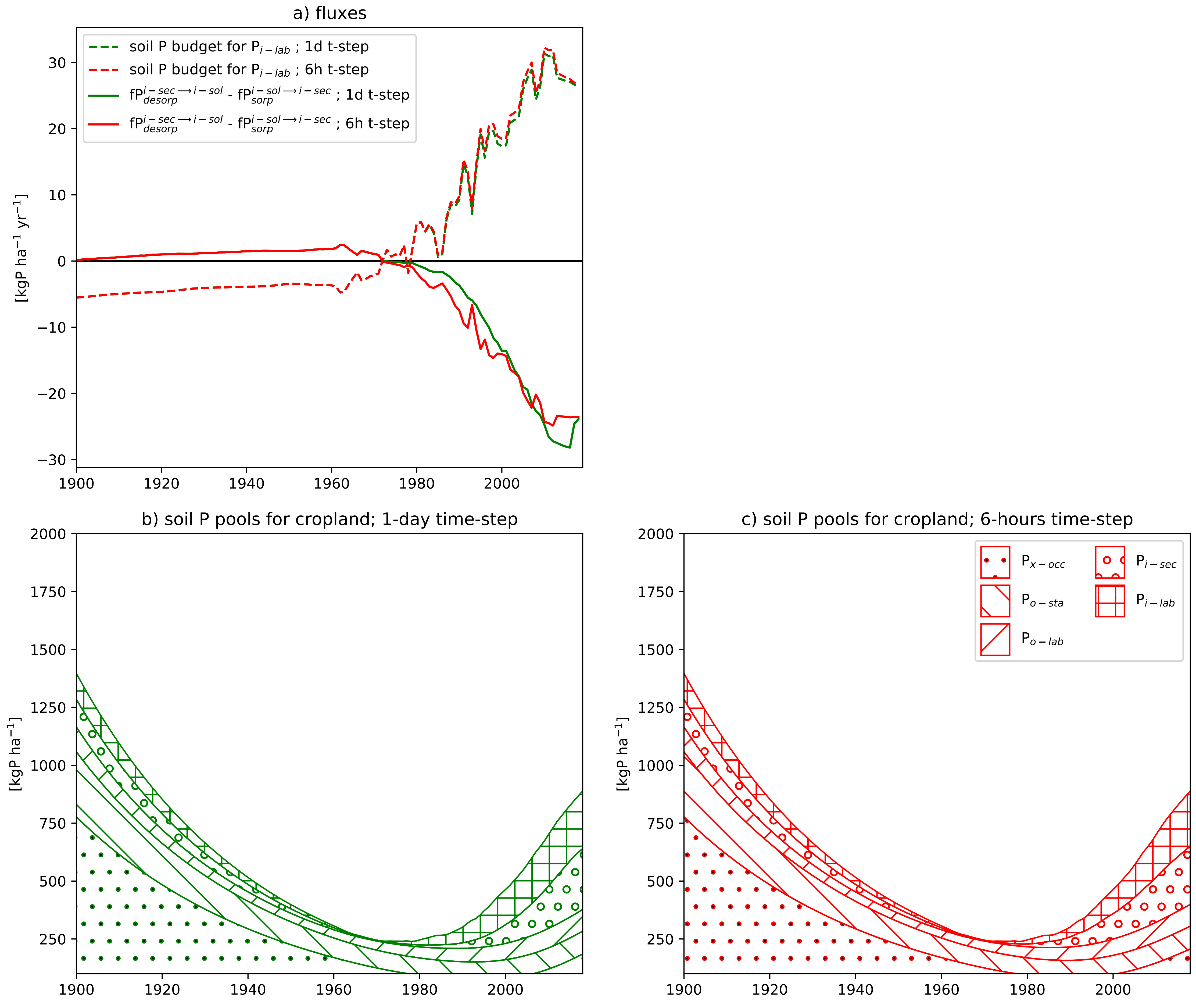
a) fluxes
soil P budget for P_{i-lab} ; 1d t-step
soil P budget for P_{i-lab} ; 6h t-step
$fP^{i-sec \rightarrow i-sol}_{desorp}$ - $fP^{i-sol \rightarrow i-sec}_{sorp}$; 1d t-step
$fP^{i-sec \rightarrow i-sol}_{desorp}$ - $fP^{i-sol \rightarrow i-sec}_{sorp}$; 6h t-step
[kgP ha^{-1} yr^{-1}]
30
20
10
0
−10
−20
−30
1900
1920
1940
1960
1980
2000
b) soil P pools for cropland; 1-day time-step
[kgP ha^{-1}]
2000
1750
1500
1250
1000
750
500
250
1900
1920
1940
1960
1980
2000
c) soil P pools for cropland; 6-hours time-step
P_{x-occ}
P_{i-sec}
P_{o-sta}
P_{i-lab}
P_{o-lab}
[kgP ha^{-1}]
2000
1750
1500
1250
1000
750
500
250
1900
1920
1940
1960
1980
2000

Figure S6 : Effect of the number of time-steps on simulated $P_{i\text{-}lab}$, $P_{i\text{-}sec}$, $P_{x\text{-}occ}$ for cropland in 2018. Here we plotted the difference (P_m simulated with 1-day time-step) – (P_m simulated with 2-days time-steps), expressed in percent of (P_m simulated with 1-day time-step) with m in {i-lab, i-sec, x-occ}. In both cases, simulations are performed with the approach configuration GPASOIL-v1.

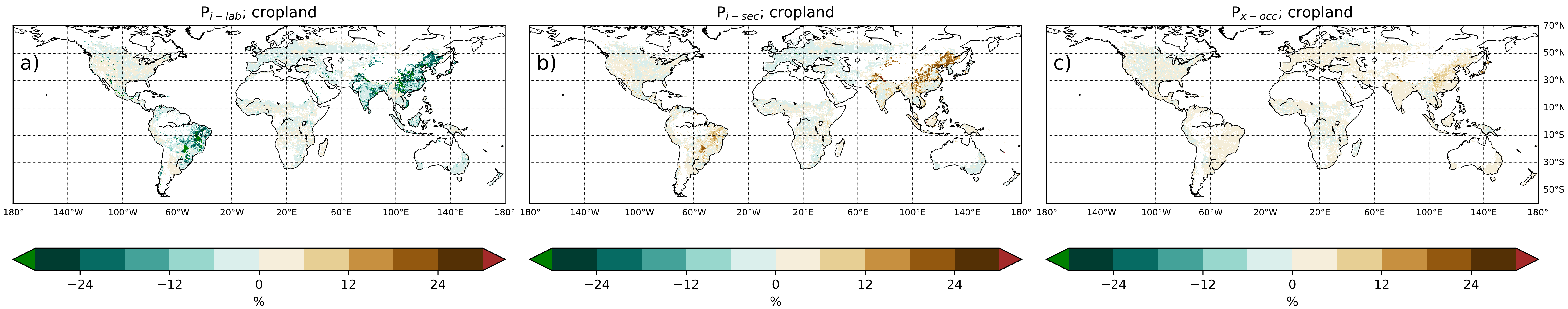

P_{i-lab}; cropland
P_{i-sec}; cropland
P_{x-occ}; cropland
a)
b)
c)
70°N
50°N
30°N
10°N
10°S
30°S
50°S
180°
140°W
100°W
60°W
20°W
20°E
60°E
100°E
140°E
180°
−24
−12
0
12
24
%

Figure S7 : Spatial distribution of $P_{i\text{-}lab}$ of cropland, $P_{i\text{-}lab}$ of grassland and $P_{x\text{-}tot}$ of cropland simulated in 2005 with (data=GPASOIL-v1, model=GPASOIL-v0) (top line) and differences with (data=GPASOIL-v0, model=GPASOIL-v0) (last line). Dataset of (Ringeval et al., 2017) was used to approach (data=GPASOIL-v0, model=GPASOIL-v0). $P_{x\text{-}tot}$ of grassland is not showed but is very similar to $P_{x\text{-}tot}$ of cropland. Sensitivity tests to evaluate the effect of using dataset describing BIOG and FARM of (data=GPASOIL-v0) instead of the ones used in (data=GPASOIL-v1) are shown (2nd, 3rd, and 4th lines). “data\FARM=GPASOIL-v1” means that all drivers but FARM have been updated (i.e. FARM= GPASOIL-v0 and other drivers=GPASOIL-v1).

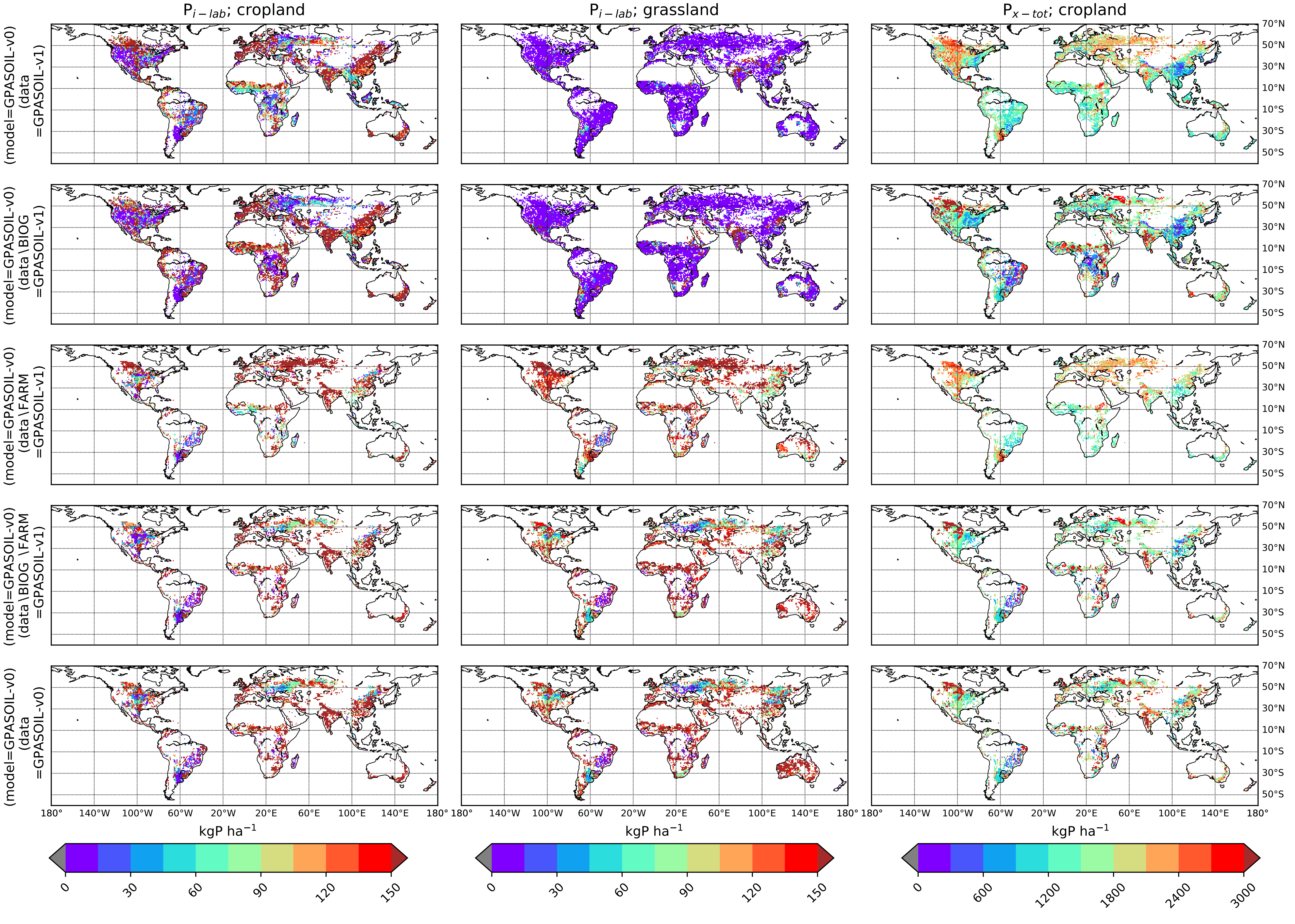

P_{i-lab}; cropland
P_{i-lab}; grassland
P_{x-tot}; cropland
(model=GPASOIL-v0) (data =GPASOIL-v1)
(model=GPASOIL-v0) (data \ BIOG =GPASOIL-v1)
(model=GPASOIL-v0) (data \ FARM =GPASOIL-v1)
(model=GPASOIL-v0) (data \ BIOG \ FARM =GPASOIL-v1)
(model=GPASOIL-v0) (data =GPASOIL-v0)
70°N
50°N
30°N
10°N
10°S
30°S
50°S
180°
140°W
100°W
60°W
20°W
20°E
60°E
100°E
140°E
180°
kgP ha^{-1}
0
30
60
90
120
150
600
1200
1800
2400
3000

Figure S8 : Soil P budget for $P_{i\text{-}lab}$ of grassland: comparison between (data=GPASOIL-v0, left column) and (data=GPASOIL-v1, right column). In GPASOIL-v0, the plant uptake of grassland was constrained as ~90 % of the total input (note that this plot focused on the input/output to $P_{i\text{-}lab}$ and thus, only a part of the total soil input were plotted).

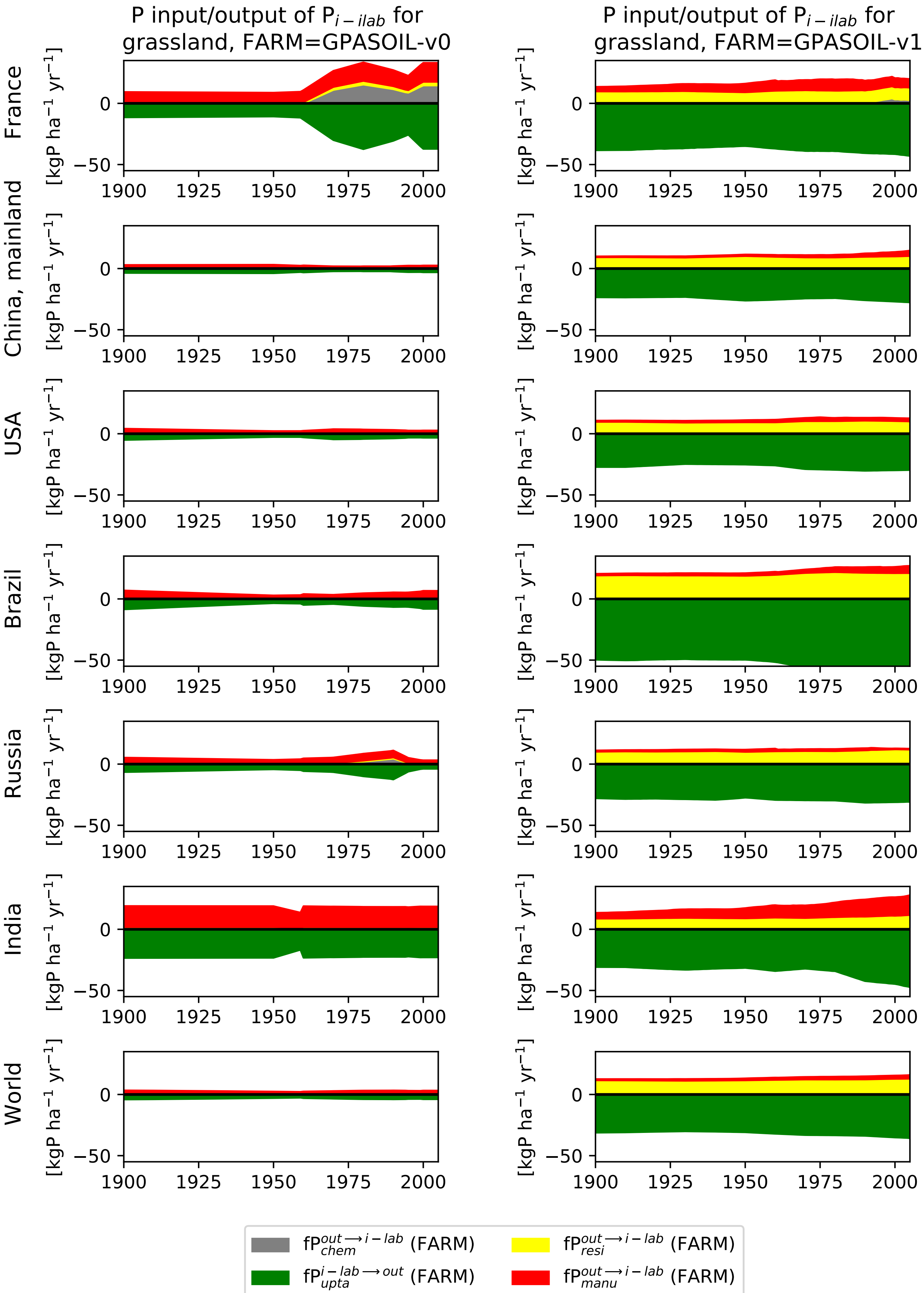
P input/output of P_{i-ilab} for grassland, FARM=GPASOIL-v0
P input/output of P_{i-ilab} for grassland, FARM=GPASOIL-v1
France
China, mainland
USA
Brazil
Russia
India
World
[kgP ha^{-1} yr^{-1}]
0
−50
1900
1925
1950
1975
2000
$fP_{chem}^{out \longrightarrow i-lab}$ (FARM)
$fP_{resi}^{out \longrightarrow i-lab}$ (FARM)
$fP_{upta}^{i-lab \longrightarrow out}$ (FARM)
$fP_{manu}^{out \longrightarrow i-lab}$ (FARM)

Figure S9: Distribution of values of $P_{i\text{-}lab}$ in both cropland and grassland in terms of number of grid-cells and effect of the model version on this distribution ((data=GPASOIL-v1, model=GPASOIL-v0) in blue, vs (data=GPASOIL-v1, model=GPASOIL-v1) in orange).

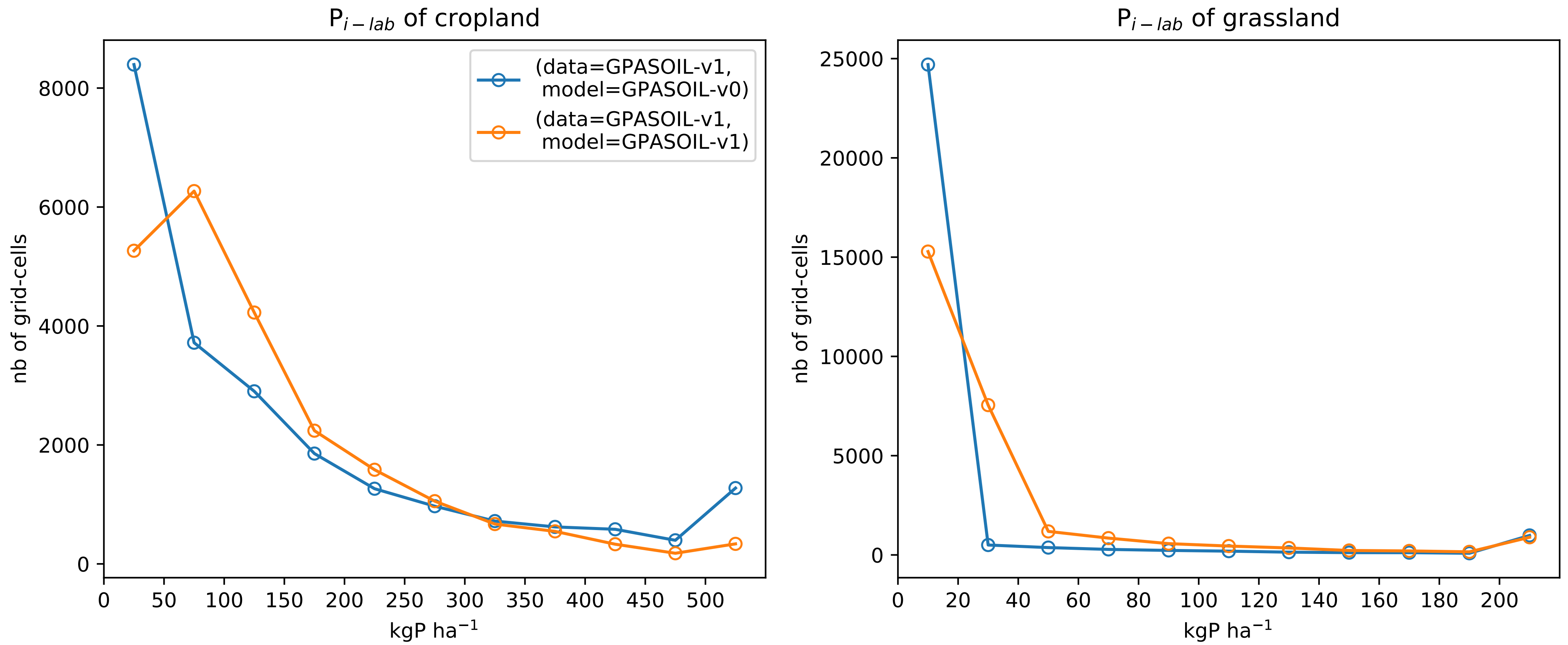

P_{i-lab} of cropland
(data=GPASOIL-v1, model=GPASOIL-v0)
(data=GPASOIL-v1, model=GPASOIL-v1)
nb of grid-cells
kgP ha^{-1}
P_{i-lab} of grassland
nb of grid-cells
kgP ha^{-1}

Figure S10 : Sensitivity of soil P pools simulated in 2018 to $P_{c,\infty}$. Three values for $P_{c,\infty}$ are tested: $5e^{-3}$, $3e^{-3}$ and $5e^{-1}$ mgP L^{-1} and compared to the simulation with the default value (0.1 mgP L^{-1}). For each value tested, the difference (soil P pools simulated with the default value – soil P pools simulated with the value tested) expressed in percent of the simulations performed with the default value is plotted. Differences in $P_{i\text{-}lab}$, $P_{i\text{-}sec}$ and $P_{x\text{-}tot}$ for cropland are plotted.

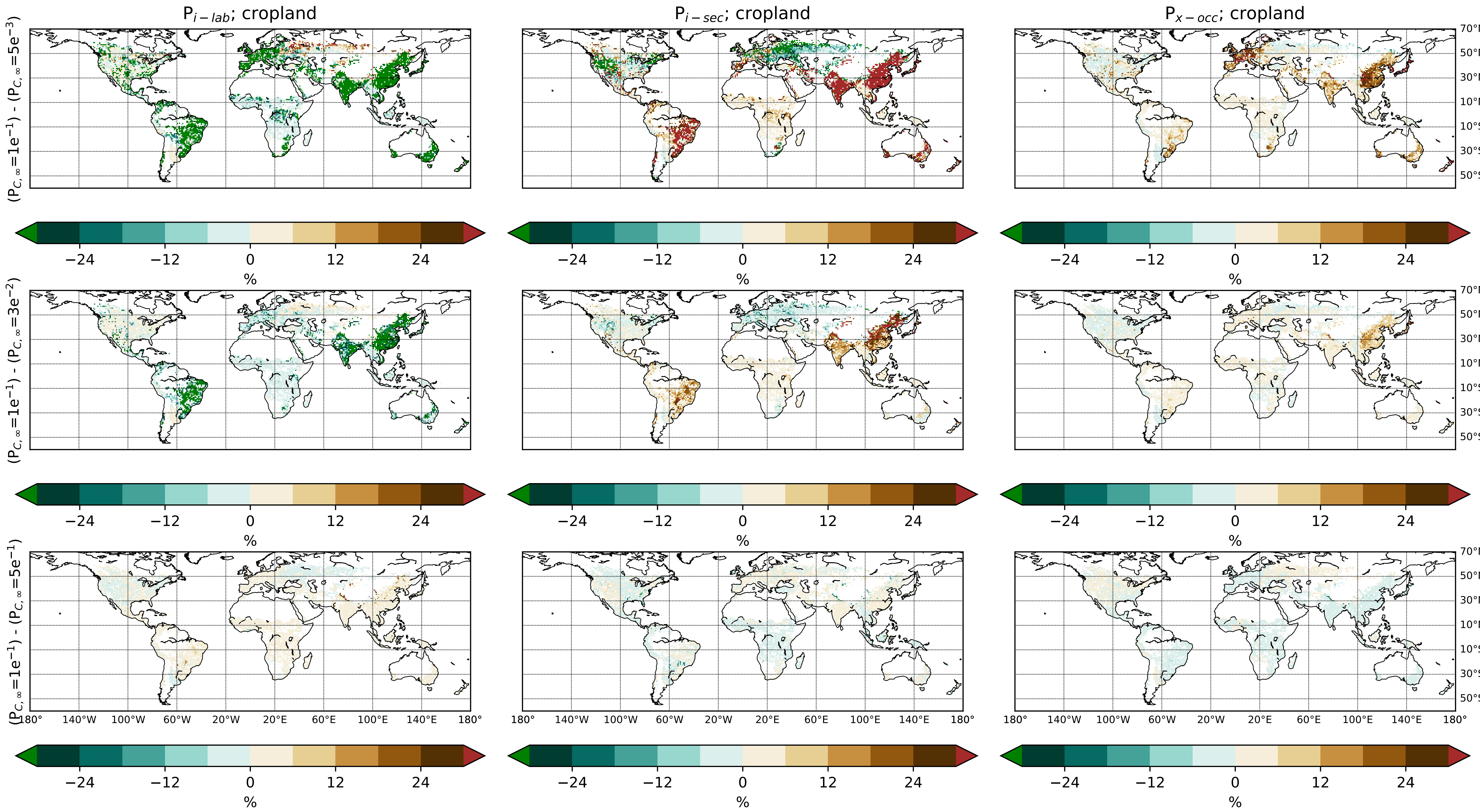

P$_{i-lab}$; cropland
P$_{i-sec}$; cropland
P$_{x-occ}$; cropland
(P$_{C,\infty}$=1e^{-1}) - (P$_{C,\infty}$=5e^{-3})
(P$_{C,\infty}$=1e^{-1}) - (P$_{C,\infty}$=3e^{-2})
(P$_{C,\infty}$=1e^{-1}) - (P$_{C,\infty}$=5e^{-1})
70°N 50°N 30°N 10°N 10°S 30°S 50°S
180° 140°W 100°W 60°W 20°W 20°E 60°E 100°E 140°E 180°
−24 −12 0 12 24
%

Figure S11: Soil P distribution provided by RMQS after regrid at the half-degree resolution then change in unit. As few sites can be encompassed within each half-degree resolution grid-cell, we provided the mean, standard-deviation and number of sites. Standard-deviation is computed only if the number of sites is larger than or equal to 5. While a detection limit for P-Olsen measurement was given RMQS, we did not excluded the sites below this threshold to prevent bias in our grid-cell averages. Data are representative to the years 2002-2009.

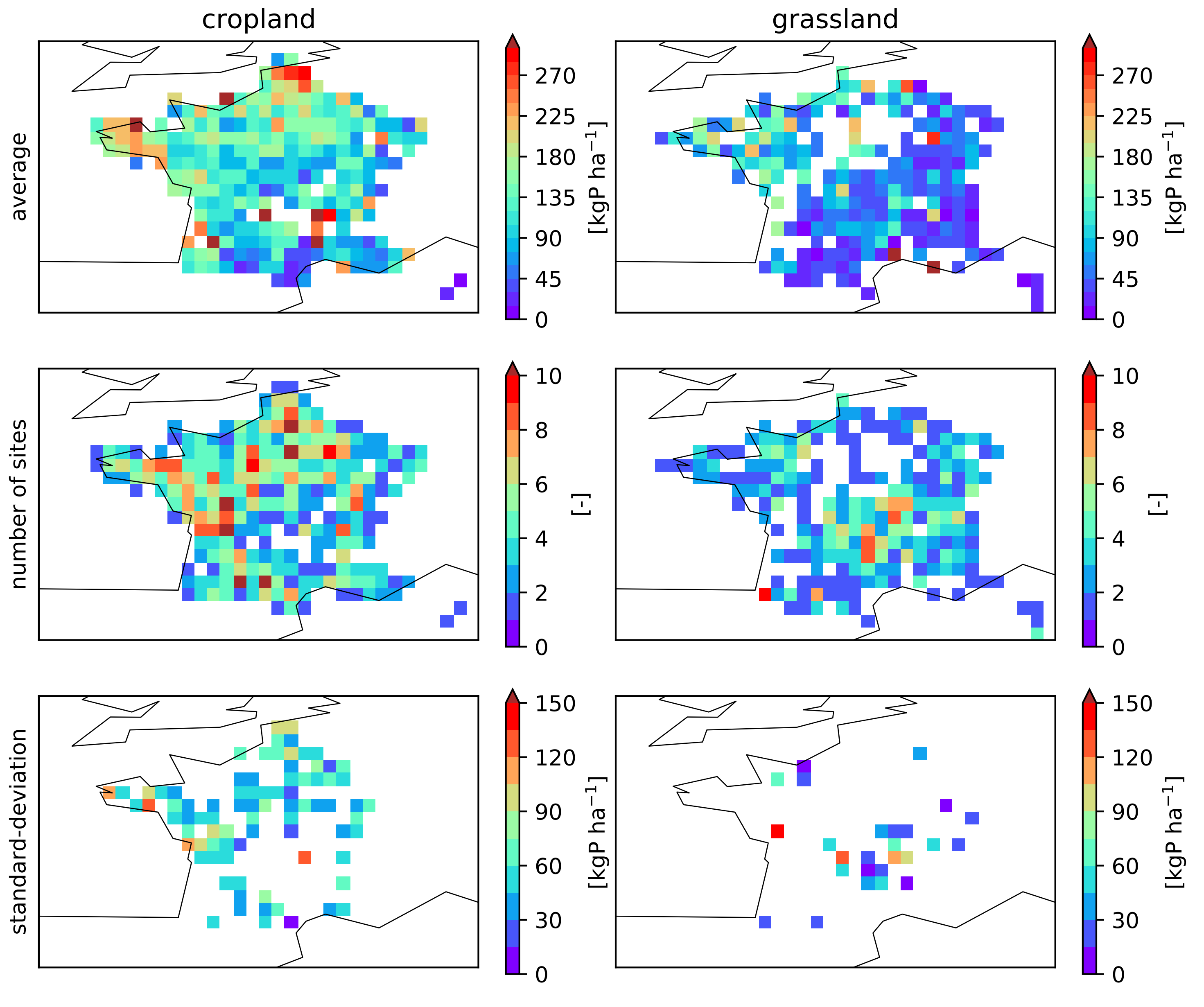
cropland
grassland
average
number of sites
standard-deviation
0
45
90
135
180
225
270
[kgP ha^{-1}]
0
2
4
6
8
10
[-]
0
30
60
90
120
150
[kgP ha^{-1}]

Figure S12: Soil P distribution provided by LUCAS after regrid at the half-degree resolution then change in unit. As few sites can be encompassed within each half-degree resolution grid-cell, we provided the mean, standard-deviation and number of sites. Standard-deviation is computed only if the number of sites is larger than or equal to 5. While a detection limit for P-Olsen measurement was given LUCAS, we did not excluded the sites below this threshold to prevent bias in our grid-cell averages. Data are representative to the year 2015.

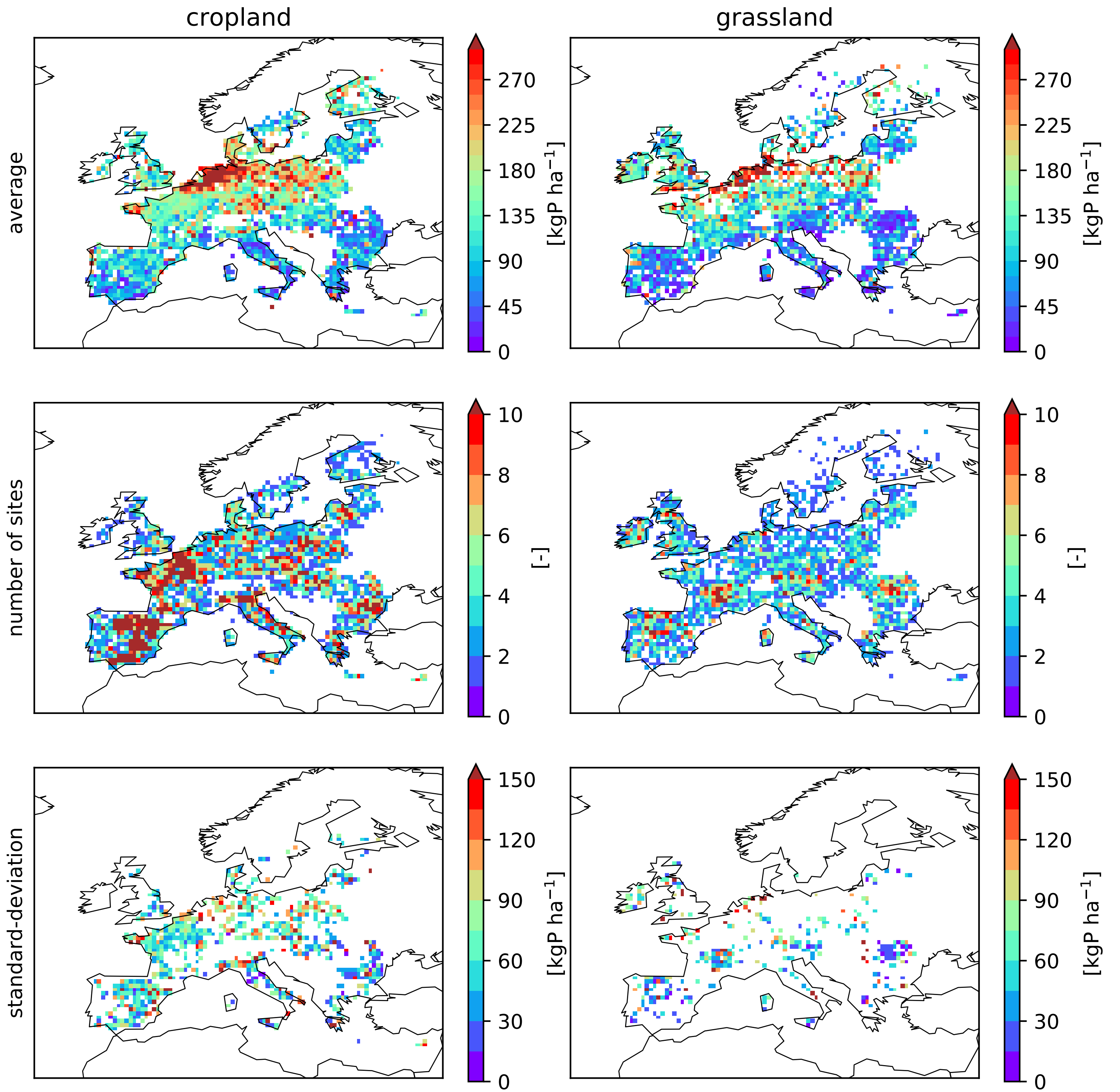

cropland
grassland
average
number of sites
standard-deviation
[kgP ha^{-1}]
[-]
0
45
90
135
180
225
270
0
2
4
6
8
10
0
30
60
90
120
150

Figure S13: Soil P distribution provided by the STS dataset after change in unit. Cropland vs grassland were not distinguished in STS and values are provided at state (for USA) or province (for Canada) scale.

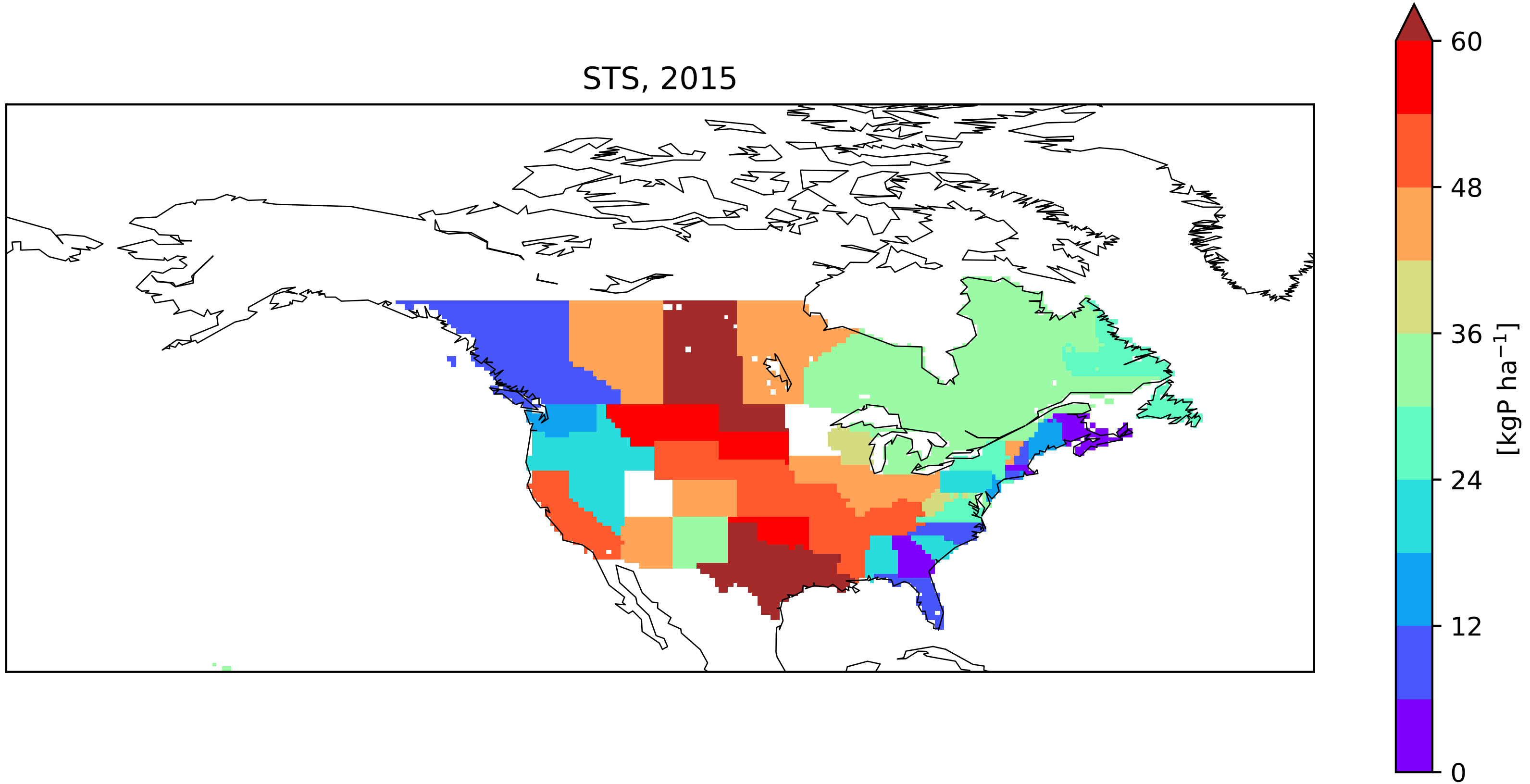

STS, 2015
60
48
36
24
12
0
[kgP ha^{-1}]

Figure S14: Treatment of $P_{i\text{-}lab}$ simulated with GPASOIL-v1.1 to allow the comparison to state/province distribution given by STS. Steps are plotted successively from the top to the bottom. Original simulations with GPASOIL-v1.1 are plotted in the 2 top panels (cropland, grassland). State/province average without distinction between cropland and grassland was plotted in 3rd panel. Finally, the decile distribution at states/province scale without distinction between cropland and grassland was plotted in bottom panel and was used for the comparison to STS in Fig.14. We excluded from the comparison the states/provinces for which our simulation does not provide 75% of the land in farm for the considered states/province.

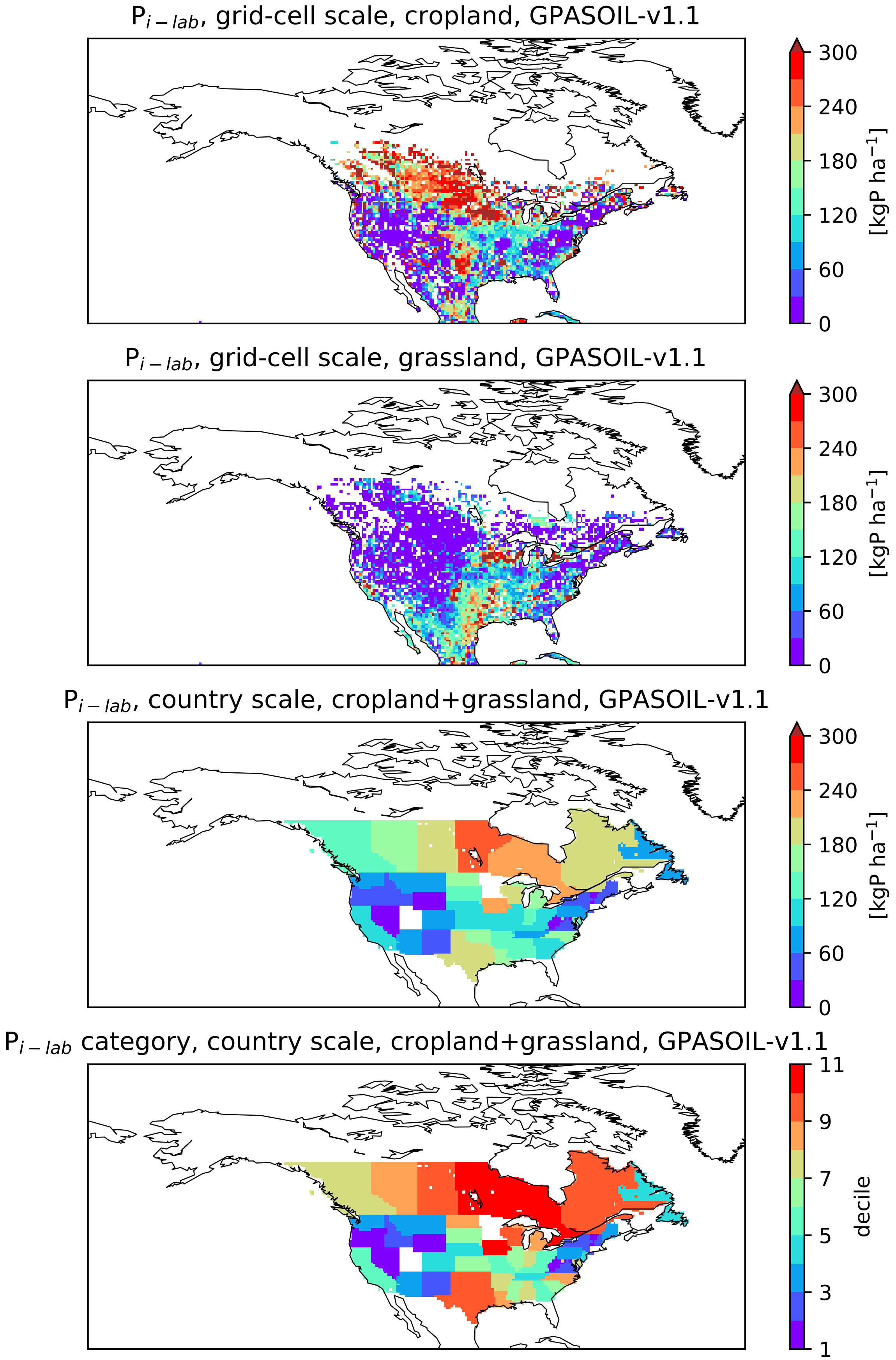

P$_{i-lab}$, grid-cell scale, cropland, GPASOIL-v1.1
P$_{i-lab}$, grid-cell scale, grassland, GPASOIL-v1.1
P$_{i-lab}$, country scale, cropland+grassland, GPASOIL-v1.1
P$_{i-lab}$ category, country scale, cropland+grassland, GPASOIL-v1.1
[kgP ha^{-1}]
0
60
120
180
240
300
decile
1
3
5
7
9
11

Figure S15: Same as Fig.13 but focusing on France only (we keep LUCAS data only if they characterized french sites).

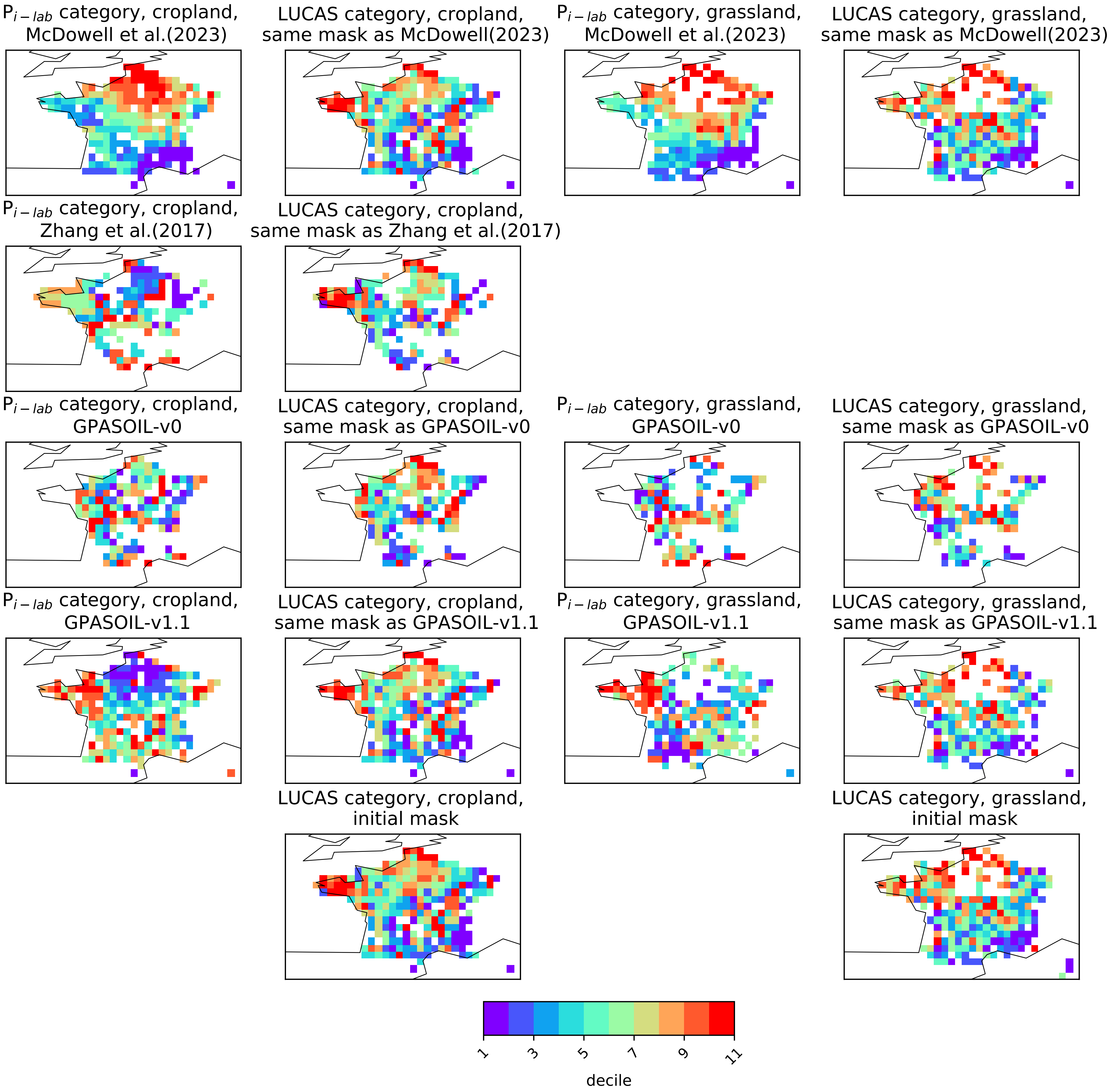
P$_{i-lab}$ category, cropland, McDowell et al.(2023)
LUCAS category, cropland, same mask as McDowell(2023)
P$_{i-lab}$ category, grassland, McDowell et al.(2023)
LUCAS category, grassland, same mask as McDowell(2023)
P$_{i-lab}$ category, cropland, Zhang et al.(2017)
LUCAS category, cropland, same mask as Zhang et al.(2017)
P$_{i-lab}$ category, cropland, GPASOIL-v0
LUCAS category, cropland, same mask as GPASOIL-v0
P$_{i-lab}$ category, grassland, GPASOIL-v0
LUCAS category, grassland, same mask as GPASOIL-v0
P$_{i-lab}$ category, cropland, GPASOIL-v1.1
LUCAS category, cropland, same mask as GPASOIL-v1.1
P$_{i-lab}$ category, grassland, GPASOIL-v1.1
LUCAS category, grassland, same mask as GPASOIL-v1.1
LUCAS category, cropland, initial mask
LUCAS category, grassland, initial mask
1
3
5
7
9
11
decile

Figure S16: Comparison between Soilgrids 2.0, RMQS and LUCAS for few soil properties over France. These soil properties were involved in the parameterizations of the soil P dynamic model (Table 10). In our approach, Soilgrids 2.0 (available at the global scale) was used to provide the values of these variables (SPRO driver) while RMQS and LUCAS, that also provide soil P measurements, were used for the evaluation of the soil P pools simulated.

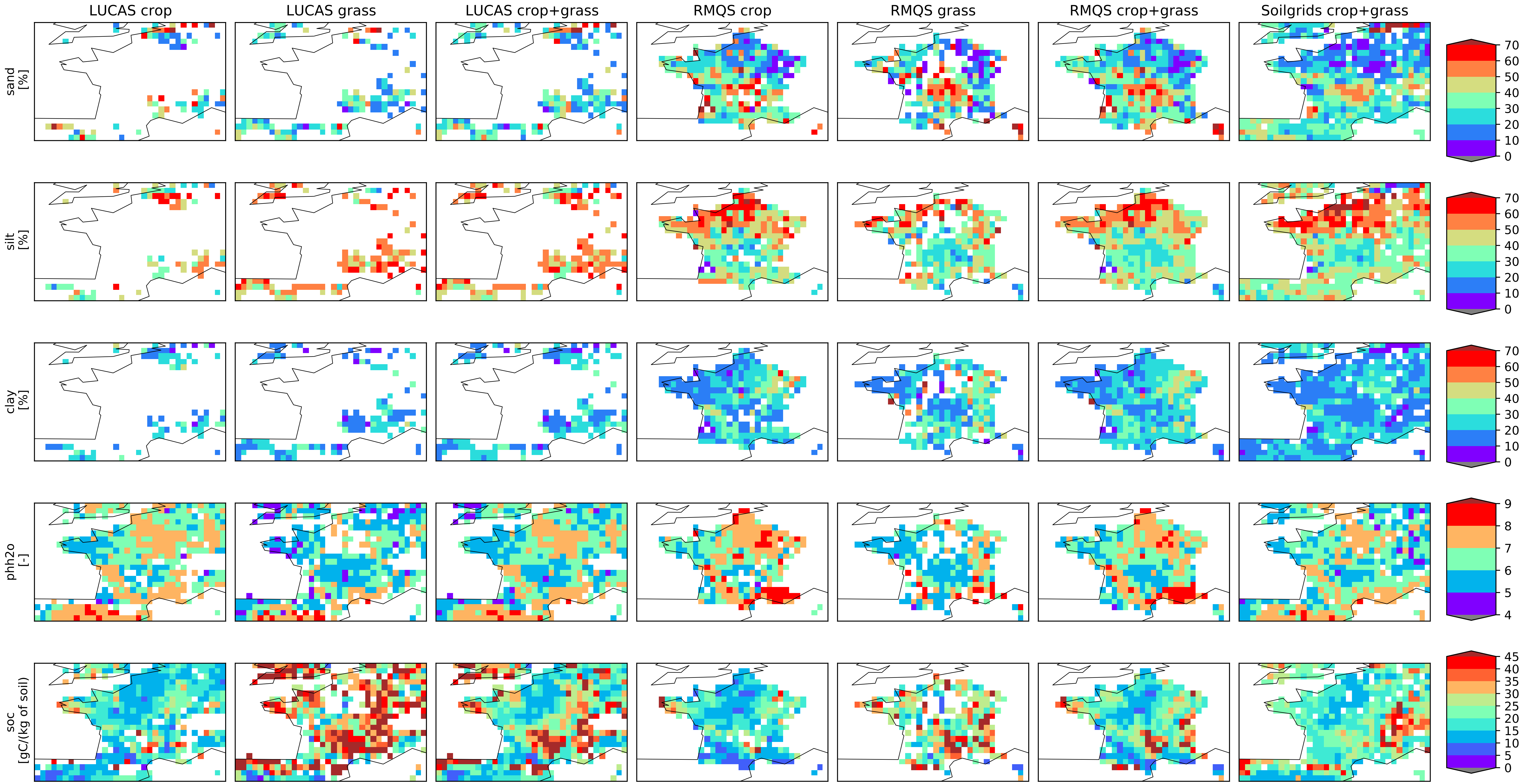

LUCAS crop
LUCAS grass
LUCAS crop+grass
RMQS crop
RMQS grass
RMQS crop+grass
Soilgrids crop+grass
sand [%]
silt [%]
clay [%]
phh2o [-]
soc [gC/(kg of soil)]
0 10 20 30 40 50 60 70
4 5 6 7 8 9
0 5 10 15 20 25 30 35 40 45

Figure S17: Strategy to attribute a value to a grid-cell belonging to a country w in 2000 for the year t before the beginning of the existence of this country (or after the end of its existence) based on country-scale information of countries x_1, x_2, x, z_1, z_2, or z that do not exist in 2000. In the figure, w, w_1 or w_2 are countries that exist in 2000 and not before their year of creation (t_{start}) or after their year of end (t_{end}). Before t_{sart}, these countries are replaced/split/aggregated by/in/into x_1, x_2, x; or after t_{end}, by/in/into z_1, z_2, z. For instance, in the 1st line, w_1 and w_2 exist in 2000 and derive from the split of x at year $t_{start}(w_1)$. The treatment of the variable depends on its nature (the so-called "yield-type" variable, Y, or "area-type" variable, A).

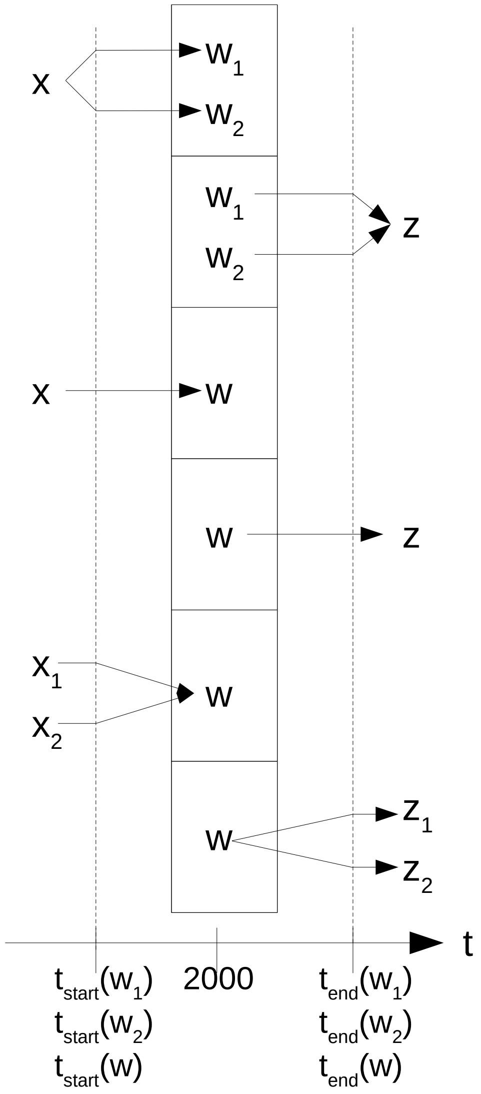

	« Yield-type » variable	« Area-type » variable
For any year $t<t_{start}(w_1)$	$Y(w_1,t)=Y(x,t)$	$A(w_1,t)=\frac{A(w_1,t_{start}(w_1))}{A(w_1,t_{start}(w_1))+A(w_2,t_{start}(w_2))}.A(x,t)$
For any year $t>t_{end}(w_1)$	$Y(w_1,t)=Y(z,t)$	$A(w_1,t)=\frac{A(w_1,t_{end}(w_1))}{A(w_1,t_{end}(w_1))+A(w_2,t_{end}(w_2))}.A(z,t)$
For any year $t<t_{start}(w)$	$Y(w,t)=Y(x,t)$	$A(w,t)=A(x,t)$
For any year $t>t_{end}(w)$	$Y(w,t)=Y(z,t)$	$A(w,t)=A(z,t)$
For any year $t<t_{start}(w)$	$Y(w,t)=\frac{Y(x_1,t).A(x_1,t)+Y(x_2,t).A(x_2)}{A(x_1,t)+A(x_2,t)}$	$A(w,t)=A(x_1,t)+A(x_2,t)$
For any year $t>t_{end}(w)$	$Y(w,t)=\frac{Y(z_1,t).A(z_1,t)+Y(z_2,t).A(z_2)}{A(z_1,t)+A(z_2,t)}$	$A(w,t)=A(z_1,t)+A(z_2,t)$
